# Assessment of inhalation toxicity of cigarette smoke and aerosols from flavor mixtures: 5‐week study in A/J mice

**DOI:** 10.1002/jat.4338

**Published:** 2022-06-08

**Authors:** Ee Tsin Wong, Karsta Luettich, Lydia Cammack, Chin Suan Chua, David Sciuscio, Celine Merg, Maica Corciulo, Romain Piault, Kumar Ashutosh, Cameron Smith, Patrice Leroy, Fabian Moine, Anneke Glabasnia, Pierrick Diana, Cecilia Chia, Ching Keong Tung, Nikolai Ivanov, Julia Hoeng, Manuel Peitsch, Kyeonghee Monica Lee, Patrick Vanscheeuwijck

**Affiliations:** ^1^ PMI R&D Philip Morris International Research Laboratories Pte Ltd Singapore; ^2^ PMI R&D Philip Morris Products S.A Neuchâtel Switzerland; ^3^ Altria Client Services LLC Richmond Virginia USA

**Keywords:** E‐cigarette, emphysema, flavor toolbox, harm reduction, inflammation, inhalation

## Abstract

Most flavors used in e‐liquids are generally recognized as safe for oral consumption, but their potential effects when inhaled are not well characterized. In vivo inhalation studies of flavor ingredients in e‐liquids are scarce. A structure‐based grouping approach was used to select 38 flavor group representatives (FGR) on the basis of known and in silico‐predicted toxicological data. These FGRs were combined to create prototype e‐liquid formulations and tested against cigarette smoke (CS) in a 5‐week inhalation study.

Female A/J mice were whole‐body exposed for 6 h/day, 5 days/week, for 5 weeks to air, mainstream CS, or aerosols from (1) test formulations containing propylene glycol (PG), vegetable glycerol (VG), nicotine (N; 2% w/w), and flavor (F) mixtures at low (4.6% w/w), medium (9.3% w/w), or high (18.6% w/w) concentration or (2) base formulation (PG/VG/N). Male A/J mice were exposed to air, PG/VG/N, or PG/VG/N/F‐high under the same exposure regimen. There were no significant mortality or in‐life clinical findings in the treatment groups, with only transient weight loss during the early exposure adaptation period. While exposure to flavor aerosols did not cause notable lung inflammation, it caused only minimal adaptive changes in the larynx and nasal epithelia. In contrast, exposure to CS resulted in lung inflammation and moderate‐to‐severe changes in the epithelia of the nose, larynx, and trachea. In summary, the study evaluates an approach for assessing the inhalation toxicity potential of flavor mixtures, thereby informing the selection of flavor exposure concentrations (up to 18.6%) for a future chronic inhalation study.

## Abstract 2

There are limited inhalation toxicology assessment data on flavor compounds in e‐liquids. A total of 38 flavor structural group representatives were selected and combined to create prototype e‐liquid formulations, which were tested against cigarette smoke in a 5‐week inhalation study. In contrast to cigarette smoke exposure, exposure to the flavor aerosols caused no notable lung inflammation and only minimal adaptive changes in the larynx and nasal epithelia. The selected flavor mixtures may be used in future chronic inhalation studies.

## 1 INTRODUCTION

Cigarette smoking is the leading cause of preventable disease and death in humans (Office of the Surgeon General, 2014, 2016). The public health strategy for reducing smoking‐related harm has been focused on preventing initiation and promoting cessation of smoking (Office of the Surgeon General, 2010, 2014; Royal College of Physicians, 2016). Some smokers are interested and quit successfully, while others continue to smoke (Babb et al., 2017; Berg et al., 2015; Hughes et al., 2004). The United States (US) Food and Drug Administration (FDA) has proposed via the Tobacco Control Act, a regulatory approach for promoting harm reduction for tobacco use based on the fact that tobacco and nicotine products present a continuum of risk, ranging from nicotine‐replacement therapy products at the low end to cigarettes at the high end of the risk continuum (Gottlieb & Zeller, 2017). Providing substantially less harmful nicotine alternatives or reduced‐risk products (RRP) and enabling complete switching to less harmful alternatives are promising approaches for achieving tobacco harm reduction (Glynn et al., 2021; McNeill et al., 2018) in individuals who continue to smoke cigarettes.

Electronic cigarettes (e‐cigarettes) or e‐vapor products are increasingly recognized as an alternative to cigarettes, because they may in theory pose lower risk than smoking does and have the potential to aid in tobacco harm reduction (Gottlieb & Zeller, 2017; Hartmann‐Boyce et al., 2020; McNeill et al., 2018). E‐cigarettes include a wide variety of electronically powered devices that are used to heat an e‐cigarette formulation or e‐liquid, which typically contain flavors, with or without nicotine, diluted in a propylene glycol‐ (PG) and/or vegetable glycerol (VG)‐based solution (Brown & Cheng, 2014). While e‐vapor products have the potential to be substantially less toxic than cigarettes, they are not free from risk (Callahan‐Lyon, 2014; National Academies of Sciences, 2018; Orr, 2014). Additionally, the role and potential health risks of flavors draw significant attention because some flavor ingredients may contribute to inhalation toxicity and many have limited available toxicological information for inhalation exposure (Higham et al., 2016; Hua et al., 2019; Kaur et al., 2018). There are a large number of flavored e‐cigarette brands and flavor ingredients used in e‐cigarette products (Cao et al., 2021; Kmietowicz, 2014; Zhu et al., 2014). Therefore, toxicological data as well as the long‐term health impact of inhaling flavors and their potential byproducts are required (Erythropel, Davis, et al., 2019; Erythropel, Jabba, et al., 2019).

Although aerosols from e‐cigarettes generally have significantly reduced levels and numbers of toxicants and carcinogens relative to cigarette smoke (CS), and switching from smoking to e‐cigarette use results in reduced levels of carcinogen/toxicant uptake biomarkers (Cahn & Siegel, 2011; Hatsukami et al., 2020), there are reports of flavors leading to toxic carbonyl and carcinogen production (Farsalinos et al., 2017; Kaur et al., 2018; Khaldoyanidi et al., 2001; Kosmider et al., 2014; Pankow et al., 2017). E‐cigarette device settings and the e‐liquid chemical composition (Geiss et al., 2016; Kosmider et al., 2014; Laino et al., 2011) can influence the analytical and, possibly, toxicological outcomes of inhalation of flavors. Certain flavor compounds such as diacetyl, 2,3‐pentanedione, and benzaldehyde are well‐known for their respiratory tract toxicity (Kaur et al., 2018; Kosmider et al., 2016; Kreiss, 2007; Soussy et al., 2016). Furthermore, the temperature of the heating coil in the e‐cigarette device may reach up to 350°C (Schripp et al., 2013), a temperature at which PG and VG are known to undergo thermal degradation to generate carbonyl compounds (e.g., formaldehyde, acetaldehyde, and acrolein), which are known respiratory tract irritants and toxicants (Geiss et al., 2016; Kosmider et al., 2014; Laino et al., 2011). In the US, the FDA promulgated regulations requiring manufacturers of e‐vapor products to submit applications for their products to demonstrate that they are appropriate for the protection of public health prior to sale (FDA, 2020). Hence, further research on the toxicity of flavor ingredients in e‐vapor aerosols is required to make informed choices on the flavor ingredients and use levels in RRPs.

While inhalation studies have been valuable in assessing the long‐term impact of specific flavors or flavored e‐cigarette products (Ha et al., 2015; Olfert et al., 2018; Werley, Kirkpatrick, et al., 2016; Wong et al., 2021), it is not always feasible to test the large number of individual flavor ingredients or combinations of formulations (Zhu et al., 2014) because of the great amount of time and number of laboratory animals required for such testing. To comprehensively test the large number of diverse flavor compounds used in e‐cigarettes, we previously performed a 90‐day sub‐chronic inhalation study in rats by employing the “read‐across” and “flavor toolbox” concepts (Ho et al., 2020; Sciuscio et al., 2022). Flavors were allocated into structurally related groups (read‐across) on the basis of the Commission Regulation No. 1565/2000 grouping approach (European Commission, 2000). Expanding on this approach, an additional 245 flavor ingredients were classified according to their structural, toxicological, and metabolic properties (Sciuscio et al., 2022). This method of compound grouping was used by the European Food and Safety Authority, among others, to evaluate the toxicity potential of various flavor ingredients (Date et al., 2020; European Commission, 2000). The flavors were then ranked for known and in silico predicted toxicity, after which a flavor group representative (FGR) was selected for each group (total 38 FGRs) and mixtures of FGRs were created (to represent the “flavor toolbox”). The aim of the 5‐week study in A/J mice was to characterize the sub‐acute toxicity observed after repeated inhalation exposure to the aerosols of prototype flavored e‐liquid formulations containing FGRs in comparison to 3R4F reference CS. Aerosols were generated by heating the formulations by using a capillary aerosol generator (CAG) at a temperature (250°C) that is representative for many e‐cigarette devices on the market (Geiss et al., 2016). We evaluated selected Organisation for Economic Co‐operation and Development (OECD) toxicological endpoints focused on the general health condition of the mice and histopathological findings in respiratory tract organs (OECD, 2018b) as well as biomarkers of exposure in urine. The results provide insights into the sub‐acute toxicity potential and concentration ranges of flavor ingredients for subsequent long‐term inhalation studies.

## 2 MATERIALS AND METHODS

### 2.1 Study design and endpoints

A total of 87 male and 174 nulliparous, non‐pregnant female A/J mice were randomly allocated to 9 experimental groups (six female and three male groups) on the basis of body weight, sex, and treatment by using a Provantis v10.2 (Instem, Staffordshire, UK) randomization sequence (Table S1; see supporting information). Each group was further subdivided on the basis of dissection endpoints (histopathology or inflammatory markers in bronchoalveolar lavage fluid [BALF]). Female A/J mice were whole‐body exposed for 6 h per day, 5 days per week, for 5 weeks to one of the following: filtered air (sham); aerosol from PG and VG with nicotine (N); aerosol from PG, VG, and N with flavors (F) at one of three concentrations—low (L, 4.6%), medium (M, 9.3%), or high (H, 18.6%); or 3R4F CS. To align closely with the nicotine and total particulate matter (TPM) content of past A/J inhalation studies involving CS (Stinn, Berges, et al., 2013; Wong et al., 2020), the target nicotine concentration in the test atmospheres of the 3R4F, PG/VG/N, and PG/VG/N/F groups was 15.0 μg/L. Because previous inhalation studies have shown that female A/J mice are more sensitive to the toxicological effects of CS and have lower spontaneous mortality rates than their male counterparts (Stinn, Berges, et al., 2013), CS exposure was omitted in male mice; only PG/VG/N and PG/VG/N/F‐H exposure were included for male mice for investigating the sex independence/dependence of the sub‐acute toxicity related to e‐vapor aerosol exposure.

### 2.2 Generation and characterization of test atmospheres

The reference cigarette, 3R4F, was purchased from the University of Kentucky (Kentucky, 2003). The cigarettes were conditioned in accordance with ISO standard 3402 (ISO3402, 1999) before mainstream CS generation as previously described (Wong et al., 2016). CS was generated by using one 30‐port rotary smoking machine (type PMRL‐G, SM2000; Burghart Messtechnik GmbH, Wedel, Germany) according to the Health Canada Intensive Smoking Protocol (Health Canada, 1999) with puff volume 55 mL, puff frequency one puff every 30 s, and ventilation block.

The 38 FGRs from different chemical structure categories were selected on the basis of known and in silico‐predicted toxicological data (Sciuscio et al., 2022). Of the 38 FGRs, 37 flavor ingredients were first prepared in the form of preblended flavors (a total of 6 preblends) from MANE (Jakarta, Indonesia). The preblends 1a, 1b, 1c, 2, 3, and 4 were mixed sequentially before addition of 2‐methyl‐butyric acid (CAS, 116‐53‐0, Merck Pte Ltd, Singapore), nicotine, ethanol, PG, VG, and water at defined mass compositions (Tables S2 and S3; see supporting information). Aerosols from the PG/VG/N and PG/VG/N/F formulations were generated using the CAG as previously described (Szostak et al., 2020). The CAG was used to generate e‐vapor aerosols for studying not only the toxicological impact of the flavor mixtures but also the potential degradation of the flavor mixtures as well as their reaction byproducts due to heating. Although the impact of the actual e‐cigarette device setup and puffing regimens on toxicological outcomes are not within the scope of this study, we selected a CAG temperatures (245–255°C) that lie within the typical operating range of the heated coil during e‐cigarette puffing (Geiss et al., 2016; Talih et al., 2019). Furthermore, the e‐vapor aerosol from the CAG was similar to the aerosol from a comparable e‐cigarette in terms of aerosol/particle size distribution and representative constituents (e.g., carbonyls) (Oldham et al., 2018; Werley, Miller, et al., 2016). The CAG generates a condensation aerosol when its output is mixed with compressed dry air flow. In this study, the concentrated aerosols produced by the CAG were further diluted (see supporting information Table S15) with filtered air to achieve the target nicotine concentration (15 μg/L) in the test atmosphere and then delivered to the exposure chambers. For the sham group, filtered fresh air was used for exposure.

The aerosol/particle size distribution (mass median aerodynamic diameter [MMAD] and geometric standard deviation [GSD]) as well as the concentrations of TPM, carbon monoxide (CO), nicotine, PG, VG, six selected flavor compounds of which each is a representative of a preblend (citronellol, eugenyl acetate, 2‐methoxy‐4‐methylphenol, ethyl maltol, triethyl citrate, and methyl anthranilate), and carbonyls (formaldehyde, acetaldehyde, acrolein, crotonaldehyde, and propionaldehyde) were determined in the test atmosphere as previously reported (Szostak et al., 2020; Wong et al., 2016) and with modifications (see supporting information). CO was only measured in the sham and 3R4F groups, as it was absent in e‐vapor aerosols (Flora et al., 2016). Additional details on the analytical methods are included in the supporting information.

### 2.3 Animals and treatment

The test facility (in Singapore) is licensed by the National Parks/Animal & Veterinary Service and accredited by the Association for Assessment and Accreditation of Laboratory Animal Care International. The care and use of the animals were in accordance with the National Advisory Committee for Laboratory Animal Research guidelines set forth in 2004 (NACLAR, 2004). The study was approved by the Institutional Animal Care and Use Committee (IACUC).

The mice, 7–9 weeks old at arrival, were obtained from The Jackson Laboratory (Bar Harbor, ME, USA). They were acclimatized to the facility for 11 days prior to the start of exposure (9–11 weeks old at the start of exposure). The 5‐week exposures included a 9‐day exposure adaptation phase, during which the exposure time of 1 h on day 1 was extended in increments of 0.5 to 1.0 h per day to the final 6 h per day on day 9. Animal husbandry procedures and in‐life monitoring of body weight (twice per week) and clinical conditions (daily) were conducted as previously described (Wong et al., 2020).

### 2.4 Biomonitoring

Urine produced during the 6‐h exposure period and 18‐h post‐exposure period were collected, combined (24‐h urine in total), and stored at ≤ − 70°C until analysis. Quantification of total nicotine metabolites (trans‐3′‐hydroxycotinine, norcotinine, cotinine, nicotine‐*N′*‐oxide, and nornicotine) was performed at Analytisch‐biologisches Forschungslabor GmbH (Planegg, Germany) by liquid chromatography–tandem mass spectrometry (LC–MS/MS) after 1,3‐diethyl‐2‐thiobarbituric acid derivatization (Rustemeier et al., 1993). Urine samples from the female sham and the PG/VG/N/F‐H groups were analyzed by Selvita Services Sp. z o.o. (Krakow, Poland) for selected flavor metabolites (eugenol and ethyl vanillic acid) by LC with triple quadrupole mass spectrometer detection (see supporting information for details).

### 2.5 Necropsy, organ weight, and histopathological evaluation

Terminal dissection was performed after the 5‐week exposure period. The mice were exposed up to the day before the scheduled dissection and were not fasted prior to dissection. The mice were anesthetized with 100 mg/kg pentobarbital (Jurox, Rutherford, NSW, Australia) via intraperitoneal injection and then exsanguinated. Terminal body weights were recorded following exsanguination. The adrenal glands, brain, heart, kidney, larynx with trachea, lungs, liver with gallbladder, ovary, spleen, testis, thymus, and uterus with cervix were removed, and their weights were recorded. Relative organ weights were derived relative to the terminal body weights. Respiratory tract organs (nose, larynx, trachea, and lungs) were dissected and fixed in 4% (w/v) formaldehyde or 10% neutral buffered formalin for approximately 48 h. The lungs were instilled with fixative via the trachea at 20‐cm H_2_O pressure before being placed in a fixative. All collected respiratory tract organs were processed into paraffin blocks. Sectioning of the nose (four transverse levels), larynx (two transverse levels), trachea (transverse section at the thyroid gland and longitudinal section at the main bifurcation), and left lung (sections at the main bronchus) were performed in accordance with OECD guideline 125 (OECD, 2010). The tissue sections were stained with hematoxylin–eosin (H&E; Sigma–Aldrich Pte Ltd, Singapore) and/or Alcian blue–periodic acid–Schiff stain (Merck Pte Ltd, Singapore; Sigma–Aldrich Pte Ltd). Histopathological evaluation was performed by Histovia GmbH (Overath, Germany) in a blinded manner. The type of findings, their severity (scored 0 to 5, with 0 indicating findings within normal limits; 1, minimal changes; 2, mild changes; 3, moderate changes; 4, marked changes; and 5, severe changes), and their incidences were recorded. All non‐respiratory tract organs (OECD, 2018b) were fixed, embedded in paraffin blocks, but no histopathology assessments were performed.

### 2.6 Lung lavage and analysis

BALF was collected as described previously (Boué et al., 2013). Additionally, recovered BALF was calculated as the difference in tube weight (and converting 1 g to 1 mL) before and after the collection of BALF. Gelatinolytic metalloproteinase (MMP) activity was measured in cell‐free BALF using the EnzChek™ test kit (Thermo Fisher, Waltham, MA, USA). Free lung cell (FLC) counts were determined using flow cytometry analysis based on the ratio of the fixed number of fluorescent beads in TruCount™ tubes (BD Biosciences, San Jose, CA, USA) to the number of FLCs and multiplied by the total volume of recovered BALF as previously described (Lietz et al., 2013). Differential FLC counts were determined by flow cytometry as previously described (Wong et al., 2020) and with modifications to include anti‐mouse, fluorochrome‐conjugated antibodies specific for CD64 (X54‐5/7.1; Biolegend, San Diego, CA, USA), CD24 (M1/69; Biolegend), and CD11b (M1/70; BD Biosciences) to distinguish alveolar macrophages, alveolar dendritic cells, and interstitial macrophages (Misharin et al., 2013). Multi‐analyte profiling was performed by using a multiplexed bead array (MCYTOMAG‐70K‐PMX‐25; Milliplex®, EMD Millipore Corp., Schwalbach, Germany) for measuring the following analytes: colony‐stimulating factor (CSF)‐2 and 3; interferon‐γ (IFN‐γ); interleukin (IL)‐1α, IL‐1β, IL‐2, IL‐4, IL‐5, IL‐6, IL‐7, IL‐9, IL‐10, IL‐12p40, IL‐12p70, IL‐13, IL‐15, and IL‐17; chemokine (C‐X‐C motif) ligand (CXCL)‐1, CXCL‐2, and CXCL‐10; chemokine (C‐C motif) ligand (CCL)‐2, CCL‐3, CCL‐4, and CCL‐5; and tumor necrosis factor‐α. Lactate dehydrogenase activity and total protein content in BALF were determined with the UniCel DxC 600i system (Beckman Coulter, Brea, CA, USA).

### 2.7 Hematology and clinical chemistry analysis

Whole blood was collected from pentobarbital‐anesthetized mice via the retro‐orbital venous sinus. The full blood count of ethylenediaminetetraacetic acid blood was determined using the Sysmex XT2000i system (Sysmex Canada Inc., Mississauga, Canada). Selected serum clinical chemistry parameters were analyzed with the UniCel DxC 600i system (Beckman Coulter, Brea, CA, USA). Relative leukocyte counts were expressed as counts relative to total leukocyte counts.

### 2.8 Statistical evaluation

Pairwise comparisons of the 3R4F, PG/VG/N, and PG/VG/N/F groups with the sham group, PG/VG/N/F groups with the 3R4F group, and PG/VG/N group with the PG/VG/N/F group were performed for each sex and endpoint separately. Because of the small group sizes, the data on flavor biomarkers in urine samples were not analyzed with inferential statistics. Inferential statistical analyses for binary, ordinal, and continuous data were performed using the Fisher exact, Wilcoxon rank sum, and Student's two‐sample *t* test, respectively. Continuous variables whose distribution showed an obvious departure from the Gaussian distribution were transformed prior to testing for group differences. All tests were two sided, with an alpha level of 0.05. The *p* values were not adjusted for multiple comparisons.

### 2.9 Supporting Information and Data Availability

Supporting information contains additional details on materials and methods, tables, and figures. Datasets, additional data visualizations, and detailed protocols are available on the INTERVALS platform at https://doi.org/10.26126/intervals.lurrz2.1.

## 3 RESULTS

### 3.1 Characterization of the test atmosphere

Daily monitoring of test atmosphere nicotine, PG, VG, and TPM concentrations indicated stable aerosol generation and delivery to the inhalation chambers, with mean aerosol concentrations consistent with the mass composition of the inhalation formulations and nicotine concentrations within ±10% of the target concentrations (see supporting information Table S6). The six representative flavor compounds quantified in the exposure chambers indicated successful aerosolization and transfer of flavor compounds from the e‐liquids to the test atmosphere, with approximately twofold differences between the low, medium, and high flavor concentration groups and closely matching concentrations between the male and female PG/VG/N/F‐H group chambers (Table 1).

**TABLE 1:** Flavor concentrations in the exposure chambers

		Selected flavors in the aerosol (μg/L)					
Group	Sex	2‐Methoxy‐4‐methylphenol	D‐L Citronellol	Ethyl maltol	Methyl anthranilate	Eugenyl acetate	Triethyl citrate
PG/VG/N	Male	<LOD	<LOD	<LOD	<LOD	<LOD	<LOD
	Female	<LOD	<LOD	<LOD	<LOD	<LOD	<LOD
PG/VG/N/F‐L	Female	2.52 ± 0.22 (3)	0.38 ± 0.05 (3)	1.87 ± 1.36 (3)	0.49 ± 0.02 (3)	1.71 ± 0.06 (3)	0.40 ± 0.13 (3)
PG/VG/N/F‐M	Female	5.20 ± 0.13 (3)	0.80 ± 0.10 (3)	4.56 ± 1.10 (3)	0.92 ± 0.08 (3)	3.44 ± 0.46 (3)	0.78 ± 0.10 (3)
PG/VG/N/F‐H	Male	10.35 ± 0.68 (3)	1.65 ± 0.05 (3)	9.20 ± 3.74 (3)	1.71 ± 0.04 (3)	6.80 ± 0.52 (3)	1.61 ± 0.44 (3)
	Female	9.47 ± 0.66 (3)	1.55 ± 0.03 (3)	9.72 ± 1.91 (3)	1.45 ± 0.14 (3)	5.99 ± 0.55 (3)	1.61 ± 0.32 (3)

Results shown are means ± standard deviation. The number of independent measurements are shown in parentheses. LODs were 0.07–0.08 μg/L, 0.07–0.08 μg/L, 0.14–0.15 μg/L, 0.10–0.11 μg/L, 0.06 μg/L, and 0.08 μg/L for 2‐methoxy‐4‐methylphenol, citronellol, ethyl maltol, methyl anthranilate, eugenyl acetate, and triethyl citrate, respectively. PG, propylene glycol; VG, vegetable glycerol; N, nicotine; F, flavors; LOD, lower limit of detection.

Aerosol characterization confirmed that the concentrations of formaldehyde, acetaldehyde, acrolein, propionaldehyde, and crotonaldehyde in the e‐vapor (PG/VG/N and PG/VG/N/F) aerosols were substantially lower than those in CS at equal nicotine concentrations (see supporting information Table S6). In the e‐vapor aerosol‐exposed groups, acetaldehyde and crotonaldehyde concentrations were higher in the flavor‐containing aerosol (PG/VG/N/F) than in the PG/VG/N aerosol. Under the acidic 2,4‐dinitrophenylhydrazine (DNPH) capturing condition, 1,1‐diethoxyethane (acetal)—one of the flavors in the aerosol—may undergo acid‐catalyzed hydrolysis to form acetaldehyde, which, in turn, reacts with DNPH to form the analytically targeted hydrazone derivative. To test this hypothesis, flavor formulations containing nicotine were prepared with and without addition of 1,1‐diethoxyethane. As shown in supporting information Table S7, the level of acetaldehyde measured in the aerosol increased as the concentration of the added flavor 1,1‐diethoxyethane increased, across the low, medium, and high flavor concentration formulations, while the acetaldehyde level in the aerosol without 1,1‐diethoxyethane was slightly higher than that in the aerosol of the PG/VG formulation (no flavors). Analysis of aerosol/particle size distributions indicated similar respirability, with MMAD of approximately 1 μm and GSD within 1.34 to 1.63, in the PG/VG/N, PG/VG/N/F, and 3R4F groups (see supporting information Table S8 and Figure S1). The MMADs were within the specifications defined for uptake and lung deposition (Asgharian et al., 2014; OECD, 2018a).

### 3.2 In‐life observations

**FIGURE 1:**
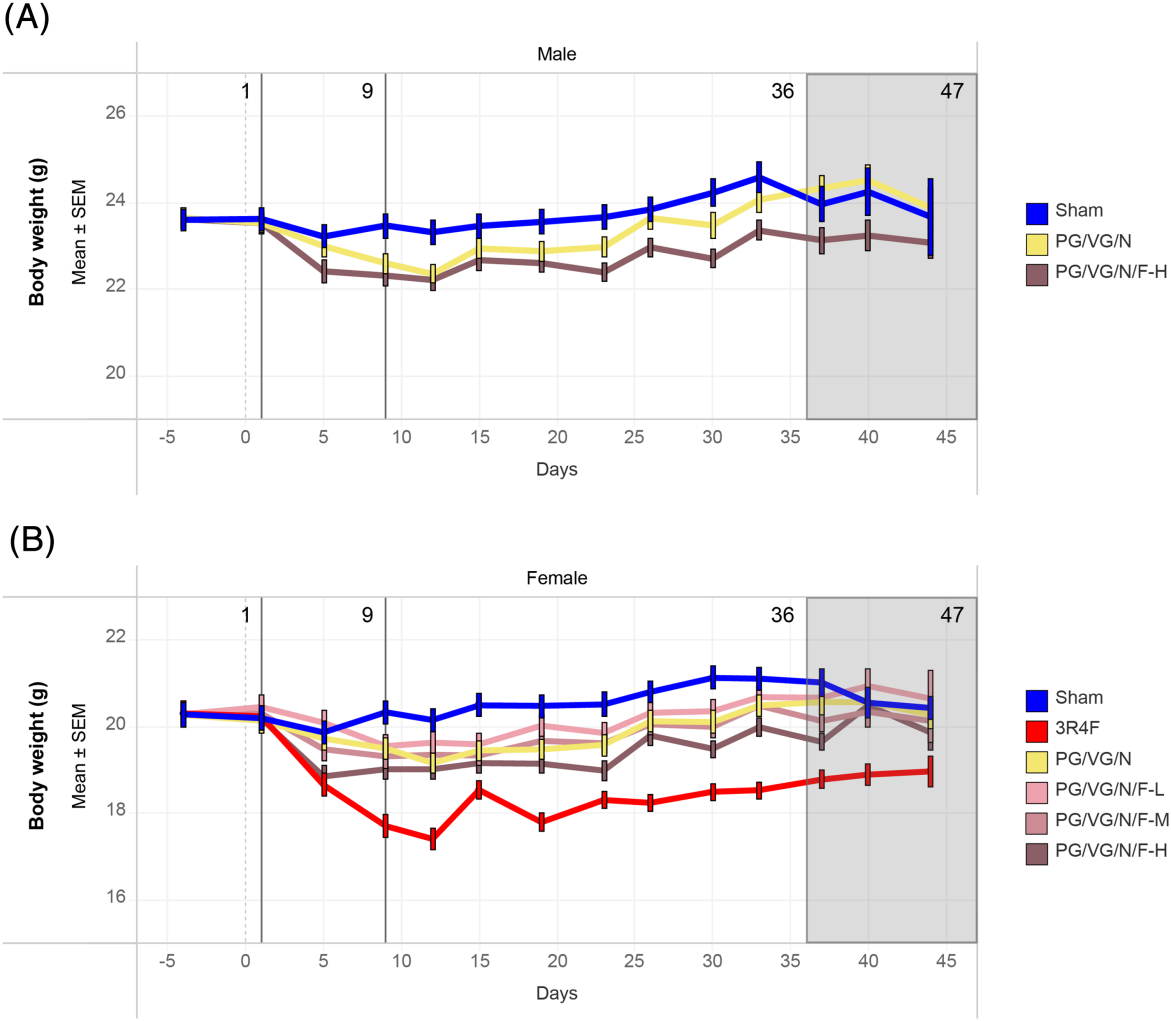
Body weight progressionThe average body weight measurements across the study period are shown for the (A) male and (B) female groups. As annotated above each graph, day 1 was the start of the exposure period; day 9 was the end of the acclimatization period; and days 36 to 47 were staggered scheduled dissection days. PG, propylene glycol; VG, vegetable glycerol; N, nicotine; F, flavors; L, low; M, medium; H, high; SEM, standard error of the mean

A transient, exposure‐related decrease in body weight was noted during the first 2 weeks of exposure acclimation, although the weight loss during this period was minimal and less pronounced in e‐vapor aerosol‐exposed male and female mice than in CS‐exposed mice. After the exposure adaptation period, animals in all groups gained body weight over time until dissection (Figure 1). By the end of week 5, the body weights of PG/VG/N/F‐H aerosol‐exposed (high flavor with nicotine) groups were slightly lower than those of the sham (male and female) and male PG/VG/N (nicotine without flavor) groups (see supporting information Figure S2). Body weights were lower in the CS group than those of the sham and PG/VG/N/F aerosol‐exposed groups. The e‐vapor aerosol and CS test atmospheres were, in general, tolerated by the mice in that they did not show severe or acute toxicity (moribundity or early death). A few incidents of transient mild‐to‐moderate tremors as well as reduced activity/exploration were noted in the PG/VG/N/F‐H aerosol‐exposed and CS groups (see supporting information Table S9). Single incidences of transient reduced grip strength and mild hyperventilation were observed in the male and female PG/VG/N/F groups, respectively. Several mice from the CS group and a few mice from the e‐vapor aerosol groups exhibited increased activity/exploration after exposure.

### 3.3 Biomonitoring

**FIGURE 2:**
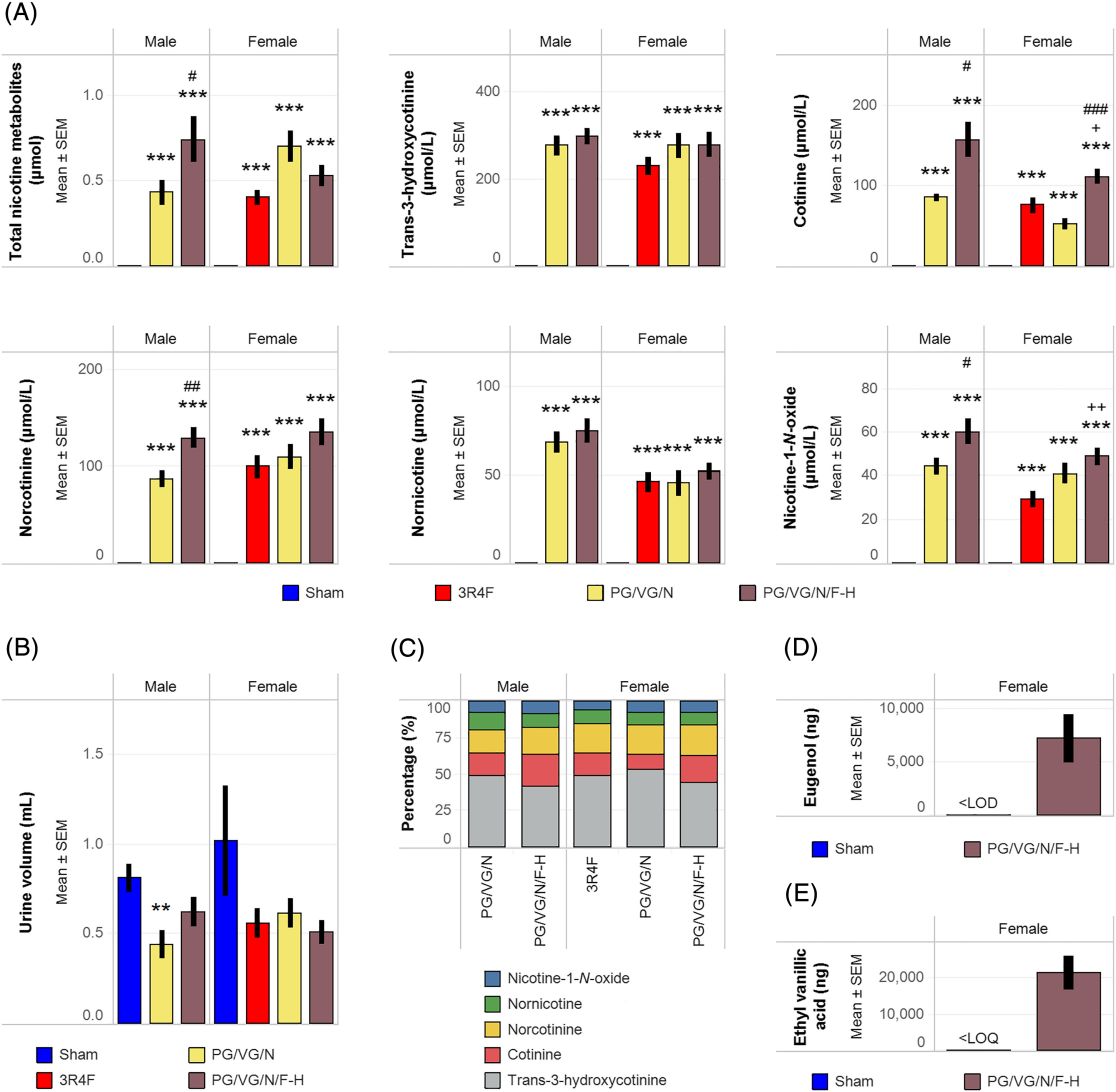
Quantification of biomarkers of exposure in urineUrinary biomarkers of exposure in 24‐h urine samples are presented as (A) total levels and concentrations of five nicotine metabolites, (B) total urine volume, (C) proportions of five nicotine metabolites relative to the sum of all nicotine metabolites, (D) total levels of eugenol, and (E) total levels of ethyl vanillic acid. Nicotine metabolites were quantified from eight mice per group. Eugenol and ethyl vanillic acid were quantified from three mice per group, and inferential statistics were not performed. ** and *** represent statistically significant differences between the treatment and sham groups at *p* ≤ 0.01 and *p* ≤ 0.001, respectively. + and ++ represent statistically significant differences between the PG/VG/N/F and 3R4F groups at *p* ≤ 0.05 and *p* ≤ 0.01, respectively. #, ##, and ### represent statistically significant differences between the PG/VG/N/F and PG/VG/N groups at *p* ≤ 0.05, *p* ≤ 0.01, and *p* ≤ 0.001, respectively. PG, propylene glycol; VG, vegetable glycerol; N, nicotine; F, flavors; L, low; M, medium; H, high; LOD, limit of detection; LOQ, limit of quantification; LOD, limit of detection; SEM, standard error of the mean

Consistent with the nicotine concentrations in CS and e‐vapor aerosol, the levels of total urinary nicotine metabolites were high only in the CS‐ and PG/VG/N and PG/VG/N/F‐H aerosol‐exposed groups (Figure 2A). The lower total urinary nicotine metabolite levels in the male PG/VG/N group (nicotine alone) compared with those in the female PG/VG/N and male PG/VG/N/F‐H (high flavor with nicotine) groups might have been because of the slightly lower urinary output in the former (Figure 2B). The lower urine output in the male PG/VG/N group may be due to inter‐animal variability and small group size. The relative proportions of the five nicotine metabolites reflected the accumulation of each nicotine metabolite (most abundant: trans‐3‐hydroxycotinine) in the urine of the exposed animals (Figure 2C). Consistent with the composition of the inhalation formulations (see supporting information Tables S2 and S3), the biomarkers of flavor compound exposure (eugenol and ethyl vanillic acid) were present in the urine samples of PG/VG/N/F‐aerosol exposed mice (flavor with nicotine) but not in those of sham‐exposed mice (Figure 2D and 2E).

### 3.4 Effects of e‐vapor exposure on lung inflammation

**FIGURE 3:**
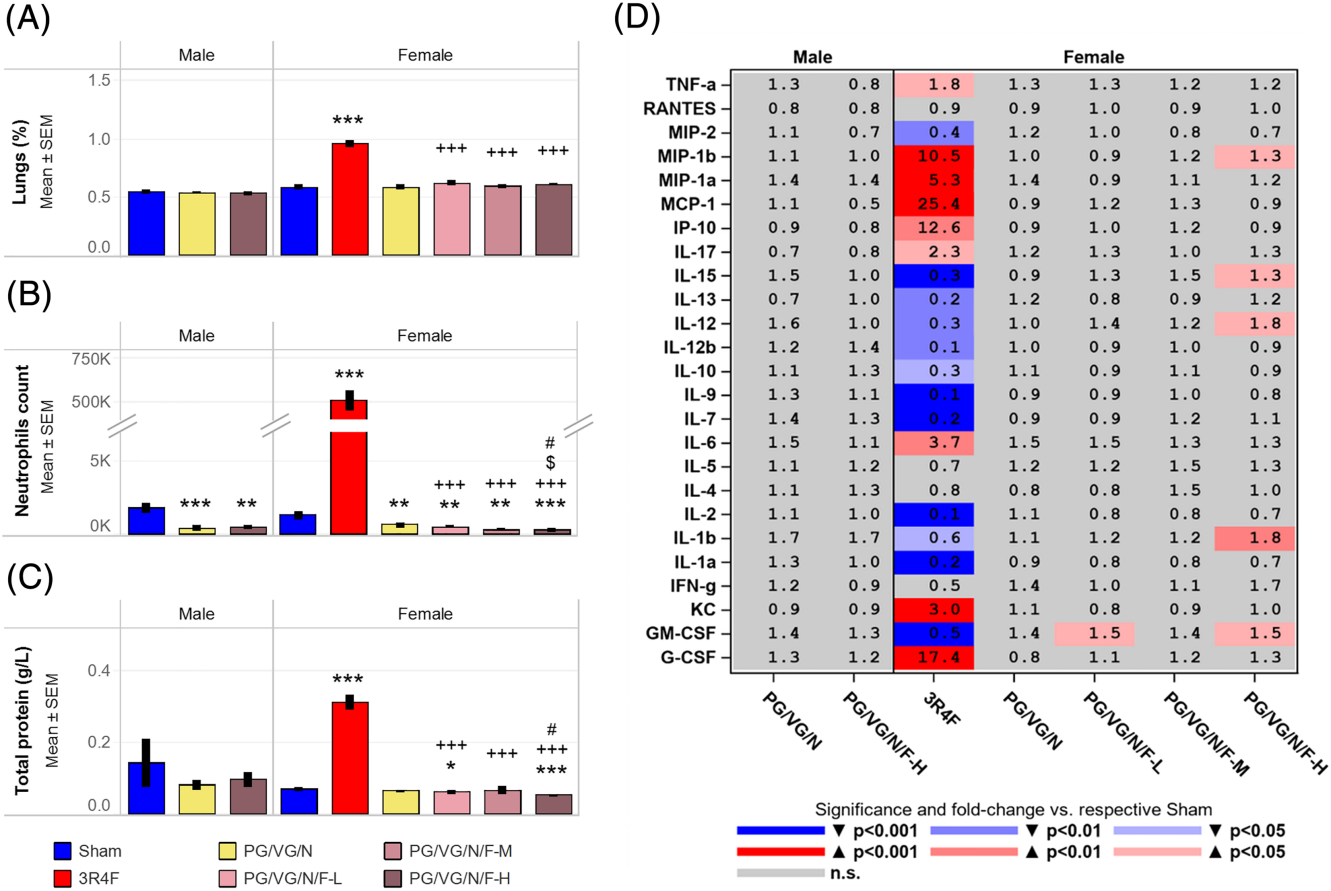
Assessment of lung inflammationData shown are (A) relative lung weights, (B) neutrophil counts in BALF, (C) total protein concentration in BALF, and (D) fold changes in BALF inflammatory mediator levels relative to the concentrations in the sham groups. Data were derived from at least 10 mice per group. **, and *** represent statistically significant differences between the treatment and sham groups at *p* ≤ 0.01, and *p* ≤ 0.001, respectively. +++ represents statistically significant differences between the PG/VG/N/F and 3R4F groups at *p* ≤ 0.001. # represents statistically significant differences between the PG/VG/N/F and PG/VG/N groups at *p* ≤ 0.05. $ represents statistically significant differences between the high flavor and low flavor groups at *p* ≤ 0.05. PG, propylene glycol; VG, vegetable glycerol; N, nicotine; F, flavors; L, low; M, medium; H, high; SEM, standard error of the mean

The female PG/VG/N (nicotine alone) and male PG/VG/N/F‐H (flavor with nicotine) groups showed marginally lower absolute lung weights (see supporting information Figure S5) than the sham groups. The absolute organ weight differences among the e‐vapor aerosol‐exposed groups were likely related to body weight effects, because the lung weights were not different when normalized to body weight (Figure 3A). In contrast to the e‐vapor aerosol‐exposed groups, the 3R4F group showed increased lung weights (absolute and relative to body weight), which were higher in the CS group than in the PG/VG/N/F groups (flavor with nicotine).

Approximately 4 mL of BALF was recovered on average in all treatment groups (see supporting information Figure S3A). The e‐vapor aerosol‐exposed mice showed lower neutrophil counts in BALF (Figure 3B) when compared to the sham group, potentially associated with their slightly lower FLC counts. Subtle changes in dendritic and T cell counts, as well as total protein concentration, and lactate dehydrogenase activity in the BALF samples were also noted in the e‐vapor aerosol‐exposed groups (Figure 3C; see supporting information Figure S3). When normalized to the total protein concentration in BALF, the concentrations of MIP‐1β, IL‐15, IL‐12, IL‐1β, and GM‐CSF in the female PG/VG/N/F‐H (high flavor with nicotine) group were slightly higher than in the sham group (Figure 3D). In contrast, the CS group consistently showed lung inflammation, with significantly higher total and differential leukocyte counts, MMP activity, and inflammatory mediator concentrations than the sham and PG/VG/N/F (flavor with nicotine) groups (Figure 3; see supporting information Figure S3 and Table S13). Of the 25 analytes measured in BALF, several (MIP‐2, IL‐15, IL‐13, IL‐12, IL‐12b, IL‐10, IL‐7, and IL‐9) were lower in normalized concentrations in the 3R4F group than in the sham group (Figure 3D). Further, lactate dehydrogenase activity, total MMP, and total protein concentrations were significantly higher in the CS group, but not in the e‐vapor aerosol groups, in comparison to the sham group (Figure 3; see supporting information Figure S3).

The absence of obvious lung inflammation was further confirmed by histopathological evaluation (see supporting information Figure S4A,B). The PG/VG/N/F‐L (low flavor with nicotine) group showed less infiltrate of unpigmented macrophages in the lungs (Table 2; see supporting information Figure S4A,B) compared to the sham group, which was considered incidental and within the normal range of variation for the physiological background of this mouse strain (Bogue et al., 2007). Increased intra‐alveolar inflammation as reflected by increased infiltrates of lymphocytes, neutrophilic granulocytes, unpigmented, and pigmented macrophages, as well as increased interstitial inflammation (perivascular) was observed in the CS group, but not in the e‐vapor aerosol‐exposed groups, relative to the sham group (Table 2). The overall data demonstrate increased concentrations of inflammatory mediators and lung inflammation only in the 3R4F CS group. The lung inflammation observed in the CS group is consistent with the findings of previous inhalation studies in mice (Phillips et al., 2016; Phillips, Veljkovic, et al., 2015; Stinn, Buettner, et al., 2013).

**TABLE 2:** Histopathology assessment of the lungs

	Male			Female					
Parameter	Sham	PG/VG/N	PG/VG/N/F‐H	Sham	3R4F	PG/VG/N	PG/VG/N/F‐L	PG/VG/N/F‐M	PG/VG/N/F‐H
Alveolar epithelium, degeneration	0.09 ± 0.09 (11)	0.00 ± 0.00 (12)	0.00 ± 0.00 (11)	0.00 ± 0.00 (11)	0.36 ± 0.20 (11)	0.00 ± 0.00 (11)	0.00 ± 0.00 (11)	0.00 ± 0.00 (11)	0.00 ± 0.00 (11)
Alveolar epithelium, hyperplasia	0.00 ± 0.00 (11)	0.00 ± 0.00 (12)	0.00 ± 0.00 (11)	0.00 ± 0.00 (11)	0.18 ± 0.12 (11)	0.00 ± 0.00 (11)	0.00 ± 0.00 (11)	0.00 ± 0.00 (11)	0.00 ± 0.00 (11)
Alveolar epithelium, nodular hyperplasia	0.09 ± 0.09 (11)	0.00 ± 0.00 (12)	0.00 ± 0.00 (11)	0.00 ± 0.00 (11)	0.00 ± 0.00 (11)	0.00 ± 0.00 (11)	0.00 ± 0.00 (11)	0.00 ± 0.00 (11)	0.00 ± 0.00 (11)
Alveolar interstitium, thickened and cell‐rich	0.00 ± 0.00 (11)	0.08 ± 0.08 (12)	0.00 ± 0.00 (11)	0.00 ± 0.00 (11)	0.00 ± 0.00 (11)	0.00 ± 0.00 (11)	0.00 ± 0.00 (11)	0.00 ± 0.00 (11)	0.00 ± 0.00 (11)
Alveolar interstitium/sub‐pleural, lymphocytic cell aggregates	0.18 ± 0.12 (11)	0.00 ± 0.00 (12)	0.09 ± 0.09 (11)	0.18 ± 0.12 (11)	0.55 ± 0.25 (11)	0.09 ± 0.09 (11)	0.00 ± 0.00 (11)^b^	0.27 ± 0.19 (11)	0.45 ± 0.16 (11)^c^
Alveolar lumen, hemorrhage	0.09 ± 0.09 (11)	0.00 ± 0.00 (12)	0.00 ± 0.00 (11)	0.00 ± 0.00 (11)	0.00 ± 0.00 (11)	0.00 ± 0.00 (11)	0.00 ± 0.00 (11)	0.09 ± 0.09 (11)	0.00 ± 0.00 (11)
Alveolar lumen, lymphocytes/plasma cells	0.00 ± 0.00 (11)	0.00 ± 0.00 (12)	0.00 ± 0.00 (11)	0.00 ± 0.00 (11)	0.82 ± 0.18 (11)^a^	0.00 ± 0.00 (11)	0.00 ± 0.00 (11)^b^	0.09 ± 0.09 (11)^b^	0.09 ± 0.09 (11)^b^
Alveolar lumen, neutrophilic granulocytes	0.00 ± 0.00 (11)	0.00 ± 0.00 (12)	0.00 ± 0.00 (11)	0.00 ± 0.00 (11)	0.55 ± 0.25 (11)^a^	0.00 ± 0.00 (11)	0.00 ± 0.00 (11)^b^	0.00 ± 0.00 (11)^b^	0.00 ± 0.00 (11)^b^
Alveolar lumen, transudate/exudate	0.00 ± 0.00 (11)	0.00 ± 0.00 (12)	0.00 ± 0.00 (11)	0.00 ± 0.00 (11)	0.82 ± 0.26 (11)^a^	0.18 ± 0.12 (11)	0.09 ± 0.09 (11)^b^	0.00 ± 0.00 (11)^b^	0.09 ± 0.09 (11)^b^
Alveolar lumen, unpigmented macrophages	0.55 ± 0.21 (11)	0.58 ± 0.26 (12)	0.55 ± 0.16 (11)	0.73 ± 0.19 (11)	2.64 ± 0.24 (11)^a^	0.36 ± 0.20 (11)	0.18 ± 0.12 (11)^a^ ^,^ ^b^	0.55 ± 0.25 (11)^b^	0.55 ± 0.21 (11)^b^
Alveolar lumen, yellow pigmented macrophages	0.00 ± 0.00 (11)	0.00 ± 0.00 (12)	0.00 ± 0.00 (11)	0.00 ± 0.00 (11)	1.91 ± 0.09 (11)^a^	0.00 ± 0.00 (11)	0.00 ± 0.00 (11)^b^	0.00 ± 0.00 (11)^b^	0.09 ± 0.09 (11)^b^
Blood vessels, congestion	0.00 ± 0.00 (11)	0.17 ± 0.17 (12)	0.00 ± 0.00 (11)	0.00 ± 0.00 (11)	0.00 ± 0.00 (11)	0.00 ± 0.00 (11)	0.00 ± 0.00 (11)	0.00 ± 0.00 (11)	0.00 ± 0.00 (11)
Emphysema	0.09 ± 0.09 (11)	0.08 ± 0.08 (12)	0.45 ± 0.21 (11)	0.09 ± 0.09 (11)	0.73 ± 0.24 (11)^a^	0.09 ± 0.09 (11)	0.00 ± 0.00 (11)^b^	0.09 ± 0.09 (11)^b^	0.09 ± 0.09 (11)^b^
Main bronchus, goblet cell hyperplasia	0.09 ± 0.09 (11)	0.17 ± 0.11 (12)	0.00 ± 0.00 (10)	0.09 ± 0.09 (11)	0.00 ± 0.00 (11)	0.00 ± 0.00 (11)	0.27 ± 0.14 (11)	0.30 ± 0.21 (10)	0.36 ± 0.20 (11)
Peri‐vascular, mono‐nuclear inflammatory cells	0.00 ± 0.00 (11)	0.25 ± 0.13 (12)	0.18 ± 0.12 (11)	0.09 ± 0.09 (11)	1.36 ± 0.34 (11)^a^	0.18 ± 0.12 (11)	0.09 ± 0.09 (11)^b^	0.36 ± 0.20 (11)^b^	0.27 ± 0.14 (11)^b^

Data from left lungs are severity scores, reported as mean ± standard error of the mean. The number of individual animal measurements is shown in parentheses.

^a^ Represent statistically significant differences *p* ≤ 0.05 between the treatment and sham groups.

^b^ Represent statistically significant differences *p* ≤ 0.05 between the PG/VG/N/F and 3R4F.

^c^ Represent statistically significant differences *p* ≤ 0.05 between the high and low flavor.

PG, propylene glycol; VG, vegetable glycerol; N, nicotine; F, flavors; H, high; M, medium; L, low.

### 3.5 Impact of e‐vapor aerosol exposure on upper respiratory tract organs

Exposure to e‐vapor aerosols induced adaptive changes (e.g., hyperplasia [nose and larynx] and squamous epithelial metaplasia [larynx]) in the epithelia of the upper respiratory tract organs (Table 3; see supporting information Figure [Link], [Link]). However, the nasal and laryngeal epithelial changes in the e‐vapor aerosol‐exposed groups were consistently and significantly less severe than those in the CS group (Table 3). All PG/VG/N/F (flavor with nicotine) groups and the female PG/VG/N (nicotine alone) group showed epithelial hyperplasia at the base of the epiglottis. All PG/VG/N/F (flavor with nicotine) groups also showed squamous epithelial metaplasia at the base of the epiglottis. Some male and female PG/VG/N/F (flavor with nicotine) groups also showed epithelial hyperplasia and squamous epithelial metaplasia at the floor of the larynx. Overall, the severity of these findings at the larynx was flavor concentration dependent and greater than in the PG/VG/N (nicotine alone) group. Epithelial hyperplasia at the vocal cords was observed in the male PG/VG/N/F (flavor with nicotine) group. Reserve cell hyperplasia at nose level 1 was observed in the male PG/VG/N/F (flavor with nicotine) group; in female mice, the finding was more severe in the PG/VG/N/F (flavor with nicotine) group than in the PG/VG/N (nicotine alone) group (Table 3). No noticeable effects on the trachea in the e‐vapor aerosol‐exposed mice were observed. Consistent with the more pronounced hyperplasia of the larynx epithelia, the 3R4F, but not the e‐vapor aerosol‐exposed groups, showed increased larynx weights (both absolute and relative to body weight) relative to the sham group (see supporting information Figures S5 and S6).

**TABLE 3:** Histopathology assessment of the nose, larynx, and trachea

	Male			Female					
Parameter	Sham	PG/VG/N	PG/VG/N/F‐H	Sham	3R4F	PG/VG/N	PG/VG/N/F‐L	PG/VG/N/F‐M	PG/VG/N/F‐H
*Nose level 1*									
Respiratory epithelium, cornification	0.00 ± 0.00 (11)	0.00 ± 0.00 (12)	0.00 ± 0.00 (11)	0.00 ± 0.00 (11)	0.18 ± 0.12 (11)	0.00 ± 0.00 (11)	0.00 ± 0.00 (11)	0.00 ± 0.00 (11)	0.00 ± 0.00 (11)
Respiratory epithelium, degeneration	0.00 ± 0.00 (11)	0.00 ± 0.00 (12)	0.00 ± 0.00 (11)	0.00 ± 0.00 (11)	0.18 ± 0.18 (11)	0.00 ± 0.00 (11)	0.00 ± 0.00 (11)	0.00 ± 0.00 (11)	0.00 ± 0.00 (11)
Respiratory epithelium, eosinophilic globules	1.09 ± 0.28 (11)	0.08 ± 0.08 (12)^a^	0.18 ± 0.18 (11)^a^	0.64 ± 0.28 (11)	0.27 ± 0.14 (11)	0.00 ± 0.00 (11)^a^	0.18 ± 0.18 (11)	0.18 ± 0.12 (11)	0.55 ± 0.28 (11)
Respiratory epithelium, hyperplasia	0.00 ± 0.00 (11)	0.08 ± 0.08 (12)	0.36 ± 0.15 (11)^a^	0.18 ± 0.12 (11)	3.09 ± 0.09 (11)^a^	0.09 ± 0.09 (11)	0.18 ± 0.12 (11)^a^	0.45 ± 0.16 (11)^b^	0.55 ± 0.16 (11)^b,c^
Respiratory epithelium, lamina propria, inflammatory cell infiltration	0.00 ± 0.00 (11)	0.00 ± 0.00 (12)	0.09 ± 0.09 (11)	0.09 ± 0.09 (11)	0.00 ± 0.00 (11)	0.00 ± 0.00 (11)	0.00 ± 0.00 (11)	0.00 ± 0.00 (11)	0.00 ± 0.00 (11)
Respiratory epithelium, squamous epithelial metaplasia	0.00 ± 0.00 (11)	0.00 ± 0.00 (12)	0.00 ± 0.00 (11)	0.00 ± 0.00 (11)	3.18 ± 0.18 (11)^a^	0.00 ± 0.00 (11)	0.00 ± 0.00 (11)^b^	0.00 ± 0.00 (11)^b^	0.00 ± 0.00 (11)^b^
Respiratory region, lumen, amorphous eosinophilic material	0.00 ± 0.00 (11)	0.00 ± 0.00 (12)	0.00 ± 0.00 (11)	0.00 ± 0.00 (11)	0.27 ± 0.14 (11)	0.00 ± 0.00 (11)	0.00 ± 0.00 (11)	0.00 ± 0.00 (11)	0.00 ± 0.00 (11)
Respiratory region, septum, goblet cell hyperplasia	0.00 ± 0.00 (11)	0.08 ± 0.08 (12)	0.00 ± 0.00 (11)	0.00 ± 0.00 (11)	0.00 ± 0.00 (11)	0.09 ± 0.09 (11)	0.00 ± 0.00 (11)	0.00 ± 0.00 (11)	0.00 ± 0.00 (11)
*Nose level 2*									
Olfactory epithelium, atrophy	0.00 ± 0.00 (11)	0.00 ± 0.00 (12)	0.00 ± 0.00 (11)	0.00 ± 0.00 (11)	1.73 ± 0.41 (11)^a^	0.00 ± 0.00 (11)	0.00 ± 0.00 (11)^b^	0.00 ± 0.00 (11)^b^	0.00 ± 0.00 (11)^b^
Olfactory epithelium, lamina propria, loss of nerve bundles	0.00 ± 0.00 (11)	0.00 ± 0.00 (12)	0.00 ± 0.00 (11)	0.00 ± 0.00 (11)	0.91 ± 0.39 (11)^a^	0.00 ± 0.00 (11)	0.00 ± 0.00 (11)^b^	0.00 ± 0.00 (11)^b^	0.00 ± 0.00 (11)^b^
Olfactory epithelium, squamous epithelial metaplasia	0.00 ± 0.00 (11)	0.00 ± 0.00 (12)	0.00 ± 0.00 (11)	0.00 ± 0.00 (11)	0.45 ± 0.31 (11)	0.00 ± 0.00 (11)	0.00 ± 0.00 (11)	0.00 ± 0.00 (11)	0.00 ± 0.00 (11)
Olfactory region, lumen, red blood cells	0.00 ± 0.00 (11)	0.00 ± 0.00 (12)	0.00 ± 0.00 (11)	0.00 ± 0.00 (11)	0.09 ± 0.09 (11)	0.00 ± 0.00 (11)	0.00 ± 0.00 (11)	0.00 ± 0.00 (11)	0.00 ± 0.00 (11)
Respiratory epithelium, eosinophilic globules	0.55 ± 0.21 (11)	0.00 ± 0.00 (12)^a^	0.18 ± 0.18 (11)	0.18 ± 0.18 (11)	0.09 ± 0.09 (11)	0.00 ± 0.00 (11)	0.09 ± 0.09 (11)	0.09 ± 0.09 (11)	0.09 ± 0.09 (11)
Respiratory epithelium, hyperplasia	0.00 ± 0.00 (11)	0.00 ± 0.00 (12)	0.00 ± 0.00 (11)	0.00 ± 0.00 (11)	1.55 ± 0.21 (11)^a^	0.00 ± 0.00 (11)	0.00 ± 0.00 (11)^b^	0.00 ± 0.00 (11)^b^	0.00 ± 0.00 (11)^b^
Respiratory epithelium, squamous epithelial metaplasia	0.00 ± 0.00 (11)	0.00 ± 0.00 (12)	0.00 ± 0.00 (11)	0.00 ± 0.00 (11)	1.45 ± 0.21 (11)^a^	0.00 ± 0.00 (11)	0.00 ± 0.00 (11)^b^	0.00 ± 0.00 (11)^b^	0.00 ± 0.00 (11)^b^
Respiratory region, amorphous eosinophilic material	0.00 ± 0.00 (11)	0.00 ± 0.00 (12)	0.00 ± 0.00 (11)	0.00 ± 0.00 (11)	0.27 ± 0.14 (11)	0.00 ± 0.00 (11)	0.00 ± 0.00 (11)	0.00 ± 0.00 (11)	0.00 ± 0.00 (11)
*Nose level 3*									
Olfactory epithelium, atrophy	0.00 ± 0.00 (11)	0.00 ± 0.00 (12)	0.00 ± 0.00 (11)	0.00 ± 0.00 (11)	1.18 ± 0.33 (11)^a^	0.00 ± 0.00 (11)	0.00 ± 0.00 (11)^b^	0.00 ± 0.00 (10)^b^	0.00 ± 0.00 (11)^b^
Olfactory epithelium, lamina propria, loss of nerve bundles	0.00 ± 0.00 (11)	0.00 ± 0.00 (12)	0.00 ± 0.00 (11)	0.00 ± 0.00 (11)	0.36 ± 0.20 (11)	0.00 ± 0.00 (11)	0.00 ± 0.00 (11)	0.00 ± 0.00 (10)	0.00 ± 0.00 (11)
Olfactory region, lumen, amorphous eosinophilic material	0.00 ± 0.00 (11)	0.00 ± 0.00 (12)	0.00 ± 0.00 (11)	0.00 ± 0.00 (11)	1.55 ± 0.28 (11)^a^	0.00 ± 0.00 (11)	0.00 ± 0.00 (11)^b^	0.00 ± 0.00 (10)^b^	0.00 ± 0.00 (11)^b^
*Nose level 4*									
Olfactory epithelium, atrophy	0.00 ± 0.00 (11)	0.00 ± 0.00 (12)	0.00 ± 0.00 (11)	0.00 ± 0.00 (10)	0.20 ± 0.20 (10)	0.00 ± 0.00 (11)	0.00 ± 0.00 (11)	0.00 ± 0.00 (11)	0.00 ± 0.00 (11)
Olfactory epithelium, eosinophilic globules	0.00 ± 0.00 (11)	0.00 ± 0.00 (12)	0.00 ± 0.00 (11)	0.00 ± 0.00 (10)	0.00 ± 0.00 (10)	0.00 ± 0.00 (11)	0.00 ± 0.00 (11)	0.00 ± 0.00 (11)	0.00 ± 0.00 (11)
Olfactory epithelium, lamina propria, loss of nerve bundles	0.00 ± 0.00 (11)	0.00 ± 0.00 (12)	0.00 ± 0.00 (11)	0.00 ± 0.00 (10)	0.10 ± 0.10 (10)	0.00 ± 0.00 (11)	0.00 ± 0.00 (11)	0.00 ± 0.00 (11)	0.00 ± 0.00 (11)
Olfactory region, lumen, amorphous eosinophilic material	0.00 ± 0.00 (11)	0.00 ± 0.00 (12)	0.00 ± 0.00 (11)	0.00 ± 0.00 (10)	1.40 ± 0.31 (10)^a^	0.00 ± 0.00 (11)	0.00 ± 0.00 (11)^b^	0.00 ± 0.00 (11)^b^	0.00 ± 0.00 (11)^b^
*Larynx, base of epiglottis*									
Epithelium, cornification	0.00 ± 0.00 (11)	0.00 ± 0.00 (11)	0.09 ± 0.09 (11)	0.00 ± 0.00 (11)	4.91 ± 0.09 (11)^a^	0.00 ± 0.00 (10)	0.00 ± 0.00 (11)^b^	0.00 ± 0.00 (11)^b^	0.09 ± 0.09 (11)^b^
Epithelium, hyperplasia	0.91 ± 0.21 (11)	1.36 ± 0.20 (11)	2.45 ± 0.25 (11)^a^, ^d,e^	0.55 ± 0.16 (11)	5.00 ± 0.00 (11)^a^	1.10 ± 0.10 (10)^a^	1.18 ± 0.12 (11)^a^, ^b,c^	1.45 ± 0.16 (11)^a^, ^b,c^	2.45 ± 0.21 (11)^a^, ^b,c^, ^d,e^
Epithelium, papillary hyperplasia/folding	0.00 ± 0.00 (11)	0.00 ± 0.00 (11)	0.00 ± 0.00 (11)	0.00 ± 0.00 (11)	0.27 ± 0.27 (11)	0.00 ± 0.00 (10)	0.00 ± 0.00 (11)	0.00 ± 0.00 (11)	0.00 ± 0.00 (11)
Epithelium, squamous epithelial metaplasia	0.36 ± 0.15 (11)	0.55 ± 0.21 (11)	2.27 ± 0.24 (11)^a^, ^d,e^	0.18 ± 0.18 (11)	5.00 ± 0.00 (11)^a^	0.20 ± 0.13 (10)	0.64 ± 0.15 (11)^a^, ^b,c^	1.27 ± 0.19 (11)^a^, ^,b,c^	1.91 ± 0.25 (11)^a^, ^b,c^, ^d,e^
Lumen, foreign material	0.00 ± 0.00 (11)	0.00 ± 0.00 (11)	0.00 ± 0.00 (11)	0.09 ± 0.09 (11)	0.00 ± 0.00 (11)	0.10 ± 0.10 (10)	0.00 ± 0.00 (11)	0.00 ± 0.00 (11)	0.09 ± 0.09 (11)
Subepithelial gland/duct, epithelial hyperplasia	0.00 ± 0.00 (11)	0.00 ± 0.00 (11)	0.00 ± 0.00 (11)	0.00 ± 0.00 (11)	2.18 ± 0.26 (11)^a^	0.00 ± 0.00 (10)	0.00 ± 0.00 (11)^b^	0.00 ± 0.00 (11)^b^	0.00 ± 0.00 (11)^b^
*Larynx, ventral depression*									
Epithelium, cornification	0.00 ± 0.00 (11)	0.00 ± 0.00 (11)	0.00 ± 0.00 (10)	0.00 ± 0.00 (11)	2.73 ± 0.69 (11)^a^	0.00 ± 0.00 (10)	0.00 ± 0.00 (10)^b^	0.00 ± 0.00 (11)^b^	0.00 ± 0.00 (11)^b^
Epithelium, squamous epithelial metaplasia	0.00 ± 0.00 (11)	0.00 ± 0.00 (11)	0.00 ± 0.00 (10)	0.00 ± 0.00 (11)	4.45 ± 0.16 (11)^a^	0.00 ± 0.00 (10)	0.00 ± 0.00 (10)^b^	0.00 ± 0.00 (11)^b^	0.00 ± 0.00 (11)^b^
Lumen, foreign material	0.00 ± 0.00 (11)	0.00 ± 0.00 (11)	0.00 ± 0.00 (10)	0.00 ± 0.00 (11)	1.00 ± 0.38 (11)^a^	0.00 ± 0.00 (10)	0.20 ± 0.13 (10)	0.00 ± 0.00 (11)^b^	0.00 ± 0.00 (11)^b^
*Larynx, vocal cord*									
Epithelium, cornification	0.00 ± 0.00 (11)	0.00 ± 0.00 (11)	0.00 ± 0.00 (10)	0.00 ± 0.00 (11)	3.89 ± 0.59 (9)^a^	0.00 ± 0.00 (10)	0.00 ± 0.00 (10)^b^	0.00 ± 0.00 (10)^b^	0.00 ± 0.00 (11)^b^
Epithelium, hyperplasia	0.55 ± 0.16 (11)	0.91 ± 0.25 (11)	1.50 ± 0.27 (10)^a^	1.00 ± 0.19 (11)	4.67 ± 0.24 (9)^a^	0.50 ± 0.17 (10)	0.90 ± 0.28 (10)^b^	1.00 ± 0.30 (10)^b^	1.45 ± 0.25 (11)^,b,c^
*Larynx, floor*									
Epithelium, cornification	0.00 ± 0.00 (11)	0.00 ± 0.00 (11)	0.00 ± 0.00 (10)	0.00 ± 0.00 (11)	4.7 ± 0.3 (10)^a^	0.00 ± 0.00 (10)	0.00 ± 0.00 (10) +	0.00 ± 0.00 (11)^b^	0.00 ± 0.00 (11)^b^
Epithelium, hyperplasia	1.00 ± 0.27 (11)	1.09 ± 0.21 (11)	1.90 ± 0.23 (10)^a^, ^b,c^	0.55 ± 0.21 (11)	4.90 ± 0.10 (10)^a^	0.60 ± 0.22 (10)	1.40 ± 0.22 (10)^a^, ^,b,c^	0.91 ± 0.16 (11)^b^	1.64 ± 0.20 (11)^a^, ^b,c^, ^d,e^
Epithelium, squamous epithelial metaplasia	0.27 ± 0.14 (11)	0.09 ± 0.09 (11)	0.50 ± 0.22 (10)	0.00 ± 0.00 (11)	4.90 ± 0.10 (10)^a^	0.00 ± 0.00 (10)	0.10 ± 0.10 (10)^b^	0.45 ± 0.16 (11)^a^, ^,b,c^	1.00 ± 0.23 (11)^a^, ^b,c^, ^d,e^
Lumen, foreign material	0.00 ± 0.00 (11)	0.00 ± 0.00 (11)	0.00 ± 0.00 (10)	0.00 ± 0.00 (11)	0.00 ± 0.00 (10)	0.00 ± 0.00 (10)	0.00 ± 0.00 (10)	0.00 ± 0.00 (11)	0.09 ± 0.09 (11)
*Trachea‐longitudinal section*									
Carina, reserve cell hyperplasia	0.00 ± 0.00 (11)	0.00 ± 0.00 (11)	0.00 ± 0.00 (9)	0.00 ± 0.00 (9)	0.44 ± 0.18 (9)^a^	0.00 ± 0.00 (11)	0.00 ± 0.00 (10)^b^	0.11 ± 0.11 (9)	0.00 ± 0.00 (9)^b^
Carina, squamous epithelial metaplasia	0.00 ± 0.00 (11)	0.00 ± 0.00 (11)	0.00 ± 0.00 (9)	0.00 ± 0.00 (9)	0.67 ± 0.24 (9)^a^	0.00 ± 0.00 (11)	0.00 ± 0.00 (10)^b^	0.11 ± 0.11 (9)	0.00 ± 0.00 (9)^b^
Epithelium, goblet cell hyperplasia	0.00 ± 0.00 (11)	0.00 ± 0.00 (11)	0.00 ± 0.00 (9)	0.33 ± 0.17 (9)	0.00 ± 0.00 (9)	0.00 ± 0.00 (11)^a^	0.00 ± 0.00 (10)	0.00 ± 0.00 (8)	0.11 ± 0.11 (9)
*Trachea‐transverse section*									
Epithelium, hyperplasia	0.00 ± 0.00 (11)	0.09 ± 0.09 (11)	0.00 ± 0.00 (11)	0.09 ± 0.09 (11)	0.00 ± 0.00 (11)	0.00 ± 0.00 (11)	0.00 ± 0.00 (11)	0.00 ± 0.00 (11)	0.00 ± 0.00 (11)
Epithelium, squamous epithelial metaplasia	0.00 ± 0.00 (11)	0.00 ± 0.00 (11)	0.00 ± 0.00 (11)	0.09 ± 0.09 (11)	0.00 ± 0.00 (11)	0.00 ± 0.00 (11)	0.00 ± 0.00 (11)	0.00 ± 0.00 (11)	0.00 ± 0.00 (11)

Data shown are severity scores, reported as mean ± standard error of the mean. The number of individual animal measurements is shown in parentheses.

^a^ Represent statistically significant differences at least *p* ≤ 0.05 between the treatment and sham groups.

^b,c^ Represent statistically significant differences at least *p* ≤ 0.05 between the PG/VG/N/F and 3R4F and the PG/VG/N/F and PG/VG/N groups, respectively.

^d,e^ Represent statistically significant differences at least *p* ≤ 0.05 between the high and low flavor and high and medium flavor groups, respectively.

PG, propylene glycol; VG, vegetable glycerol; N, nicotine; F, flavors; H, high; M, medium; L, low.

### 3.6 Impact of e‐vapor aerosol exposure on spleen, thymus, and adrenal gland

**FIGURE 4:**
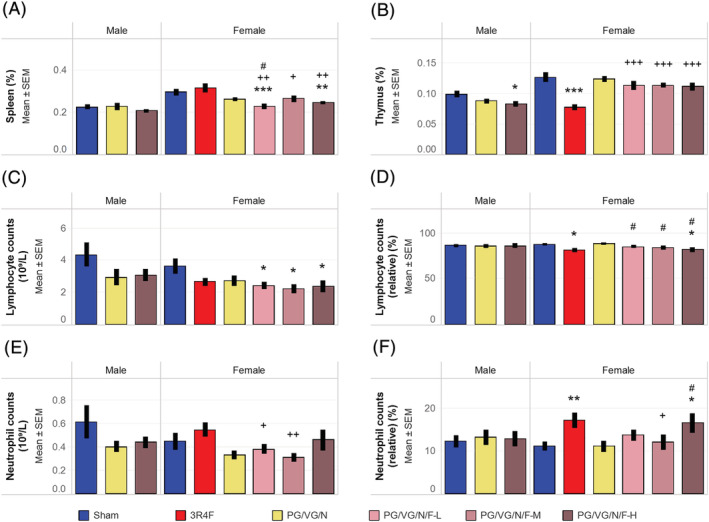
Organ weights and differential leukocyte countsResults shown are (A) relative spleen weights, (B) relative thymus weights, (C) absolute lymphocyte counts, (D) relative lymphocyte counts, (E) absolute neutrophil counts, and (F) relative neutrophil counts. Relative organ weights were from at least 10 mice per group. The average absolute and relative cell counts were from 6 to 10 mice per group. Relative counts are presented relative to the total leukocyte counts. *, **, and *** represent statistically significant differences between the treatment and sham groups at *p* ≤ 0.05, *p* ≤ 0.01, and *p* ≤ 0.001, respectively. +, ++, and +++ represent statistically significant differences between the PG/VG/N/F and 3R4F groups at *p* ≤ 0.05, *p* ≤ 0.01, and *p* ≤ 0.001, respectively. # represents statistically significant differences between the PG/VG/N/F and PG/VG/N groups at *p* ≤ 0.05. PG, propylene glycol; VG, vegetable glycerol; N, nicotine; F, flavors; L, low; M, medium; H, high; SEM, standard error of the mean

The female PG/VG/N/F‐L and PG/VG/N/F‐H (flavor with nicotine) groups showed lower relative spleen weights than the sham group (Figure 4). The female PG/VG/N (nicotine alone) and male PG/VG/N/F‐H groups (see supporting information Figure S5) showed marginally lower absolute spleen weights than the sham groups, but these differences were not significant when the organ weights were normalized to body weight. The absolute and relative spleen weights of the CS group were not significantly different from those of the sham group. The relative spleen weights were lower in all female PG/VG/N/F groups (flavor with nicotine) than in the CS group.

The male PG/VG/N/F‐H (flavor with nicotine) and CS groups showed lower absolute and relative thymus weights than the sham groups (Figure 4; see supporting information Figure S5). The absolute thymus weights were marginally lower in the male PG/VG/N and female PG/VG/N/F‐H groups than in the sham groups, but the differences were not statistically significant when the organ weights were normalized to body weight. The absolute and relative thymus weights were lower in the CS group than in the PG/VG/N/F groups (flavor with nicotine).

No treatment‐related changes in relative adrenal gland weight were noted (see supporting information Figure S6). The lower adrenal gland weights in the female PG/VG/N/F‐H group (high flavor with nicotine) (see supporting information Figure S5) were likely due to body weight effects, as these differences were no longer observed when normalized to body weights (see supporting information Figure S6). The relative adrenal gland weights were marginally (but not significantly) lower in the CS group than in the sham group.

The female PG/VG/N/F (flavor with nicotine) groups had lower absolute lymphocyte counts than the sham group (Figure 4). The male e‐vapor aerosol‐exposed groups as well as the female CS and PG/VG/N (nicotine alone) groups showed marginal reductions in absolute lymphocyte counts; however, these differences were not statistically significant. The corresponding relative lymphocyte counts were lower in the CS and female PG/VG/N/F‐H (high flavor with nicotine) groups than the sham groups. No differences were noted in absolute lymphocyte counts between the CS and PG/VG/N/F (flavor with nicotine) groups or between the PG/VG/N/F (flavor with nicotine) and PG/VG/N (nicotine alone) groups.

The female CS and PG/VG/N/F‐H (high flavor with nicotine) groups showed marginally higher relative neutrophil counts, but there were no statistically significant changes in their absolute neutrophil counts when compared to the sham group (Figure 4). While the absolute neutrophil counts were lower in the female PG/VG/N/F‐L and PG/VG/N/F‐M (low and medium flavor with nicotine) groups compared to the CS group, they were not statistically significantly different from those in the sham group. There were no obvious CS‐ or e‐vapor aerosol‐related changes in the total leukocyte or platelet counts (see supporting information Table S11).

The erythrocyte counts and red blood cell indices in the e‐vapor aerosol‐exposed groups did not change or only changed subtly (see supporting information Table S10 and Figure S7). The results showed marginally lower mean corpuscular hemoglobin concentrations in the male PG/VG/N (nicotine alone) and PG/VG/N/F‐H (flavor with nicotine) groups as well as lower absolute and relative reticulocyte counts in the female PG/VG/N/F‐L and PG/VG/N/F‐M (low and medium flavor with nicotine) groups than in the respective sham groups. These changes were within the published reference ranges (Bogue et al., 2007). Only the CS group showed higher erythrocyte counts and increased red blood cell indices than the sham groups. The lower relative reticulocyte count in the CS group was likely associated with the increase in total erythrocyte count, consistent with published data and linked to high CO exposure (Phillips et al., 2016; Phillips, Veljkovic, et al., 2015; Wong et al., 2020).

### 3.7 Impact of e‐vapor aerosol exposure on other organ weights

Only the PG/VG/N/F‐L group (low flavor with nicotine) showed lower absolute and relative (to body weight) ovary weights than the sham group (see supporting information Figures S5 and S6). No statistically significant differences in ovary weights were noted between the CS and PG/VG/N/F groups (flavor with nicotine). The marginally lower absolute ovary weights in the CS, PG/VG/N (nicotine alone), and PG/VG/N/F‐M (medium flavor with nicotine) groups were probably related to body weight effects, because the differences were not significant when the organ weights were normalized to body weight. Only the PG/VG/N/F‐L group (low flavor with nicotine) showed lower absolute and relative (to body weight) uterus weights. There was no flavor concentration‐dependent effect on uterus weight relative to the sham group (Figures S5 and S6). The increase in relative (to body weight) kidney, liver, heart, and testis weights without a corresponding increase in the absolute organ weights in the CS or e‐vapor aerosol‐exposed groups is likely secondary to the differences in body weight (see supporting information Figure S6). There were no treatment‐related changes in the organ weights of the seminal vesicle or epididymis (see supporting information Figures S5 and S6).

### 3.8 Serum clinical chemistry analysis

**FIGURE 5:**
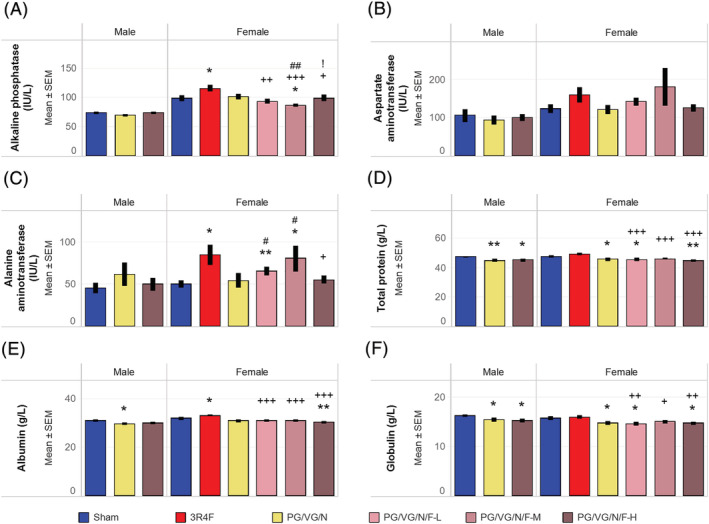
Serum clinical chemistry parameters Results of quantification of serum analytes representative of liver function are shown for (A) alkaline phosphatase activity, (B) aspartate aminotransferase activity, (C) alanine aminotransferase activity, (D) total protein concentration, (E) albumin concentration, and (F) globulin concentration. The average analyte concentrations were from 10 to 11 mice per group. * and ** represent statistically significant differences between the treatment and sham groups at *p* ≤ 0.05 and p ≤ 0.01, respectively. +, ++, and +++ represent statistically significant differences between the PG/VG/N/F and 3R4F groups at *p* ≤ 0.05, *p* ≤ 0.01, and *p* ≤ 0.001, respectively. # and ## represent statistically significant differences between the PG/VG/N/F and PG/VG/N groups at *p* ≤ 0.05 and *p* ≤ 0.01, respectively. ! represents statistically significant differences between the high flavor and medium flavor groups at *p* ≤ 0.05. PG, propylene glycol; VG, vegetable glycerol; N, nicotine; F, flavors; H, high; M, medium; L, low; SEM, standard error of the mean

The activities of alanine and aspartate aminotransferases (not statistically significant) were slightly higher in the female PG/VG/N/F‐L and PG/VG/N/F‐M (low and medium flavor with nicotine) groups (Figure 5). There were subtle changes in alanine aminotransferase and alkaline phosphatase activities in the PG/VG/N/F‐L and PG/VG/N/F‐M (low and medium flavor with nicotine) groups and in serum protein concentrations in the PG/VG/N (nicotine alone) and PG/VG/N/F (flavor with nicotine) groups without any concentration‐dependent changes and within the published ranges (Bogue et al., 2007). In contrast, albumin concentration as well as alkaline phosphatase and alanine aminotransferase activities were higher in the CS group than in the sham or PG/VG/N/F (flavor with nicotine) groups (Figure 5). Neither the e‐vapor aerosol exposure groups nor the CS groups showed any obvious exposure‐related change relative in glucose, total cholesterol, triglyceride, or total bilirubin concentrations (see supporting information Table S12).

## 4 DISCUSSION

This 5‐week inhalation study demonstrates the sub‐acute responses in A/J mice upon exposure to flavor‐containing and non‐flavor‐containing PG/VG/N aerosols in comparison to the responses observed in sham or CS exposure. The CS and PG/VG/N aerosols were matched by nicotine concentration (15 μg/L) in the test atmosphere. On the basis of the study exposure regimen and a body surface area conversion factor of 12.3 (CDER, 2005; Reagan‐Shaw et al., 2008), the estimated delivered dose was 6.5 mg/kg or 129.2 μg nicotine per day, which corresponds to a human equivalent nicotine dose of about 31.6 mg/day. This estimated human equivalent nicotine dose approximates that of 31 cigarettes or ~1.5 packs of cigarettes smoked per day. The estimated human equivalent nicotine dose approximates to the lower limit of nicotine content and consumption rates in humans. Due to the wide range of electronically powered devices and e‐cigarette formulations (Brown & Cheng, 2014) that may impact nicotine delivery and uptake (Davis & Curvali, 1999; Shao & Friedman, 2020), the extrapolations to human nicotine consumption are limited to few citations in the literature. For examples, previous studies have described an estimated daily consumption of 5.1 mL of an e‐liquid containing 7.2–10.3 mg nicotine/mL in a Greek population (Diamantopoulou et al., 2019), 3.35 mL/day of e‐liquids containing ≤20.0 mg nicotine/mL in the European Union (Action on Smoking and Heath, 2021), an average 18.3 mg nicotine equivalent uptake from a 5% JUUL pod (nicotine salt) (Prochaska et al., 2021), and 9 mL/day of e‐liquids containing an average of 3.3 mg nicotine/mL in e‐vapor products with adjustable power (Diamantopoulou et al., 2019; Smets et al., 2019). On the basis of the flavor concentrations used in the PG/VG/N/F‐H (high flavor exposure) groups, the estimated human equivalent doses for the six analyzed flavors range from 3.4 to 19.9 mg/day (see supporting information Table S15). Ethyl maltol is frequently detected and at a wide range of concentrations in commercial e‐liquids (1 mg/mL to >10 mg/mL) (Hua et al., 2019; Omaiye et al., 2019; Tierney et al., 2015). At this range, and on the basis of an estimated daily consumption of 5.1 mL of e‐liquid, the estimated human equivalent dose tested for ethyl maltol approximates to the lower half of the actual human consumption rate. Methyl anthranilate is less frequently present and at lower concentrations in commercial e‐liquids (approximately 0.1 mg/mL) (Hua et al., 2019). At this concentration and at 5.1‐mL daily consumption rate, the estimated human equivalent dose tested for methyl anthranilate exceeds the estimated human consumption rate.

We have previously demonstrated the concept of using flavor representatives for assessing the toxicity of flavors in an inhalation study in a 90‐day OECD study (Ho et al., 2020). In the current study, we included a greater number of individual flavors (a total of 245 flavors), grouped them on the basis of structural similarity, and then selected a FGR (38 FGRs in total) with the most severe toxicological profile from each structural group (Sciuscio et al., 2020, 2022). The selected concentrations of individual flavor chemicals were similar to or higher than those typically used in commercial e‐liquids (Behar et al., 2018; Hua et al., 2019; Omaiye et al., 2019). In addition, the total flavor load (18.6%) of the e‐liquids used in this study was over twofold higher compared to the previous study (8.8% w/v) (Ho et al., 2020). This increase widens the exposure scenario for e‐liquid flavor testing. The formulations were heated with a device‐agnostic aerosol generator (CAG), which was within the temperature range of the aerosolization process of many e‐cigarette devices (Geiss et al., 2016; Talih et al., 2019; Werley, Miller, et al., 2016), so that any potential thermally generated byproducts of toxicological concern could be assessed. To demonstrate the comparability of using a CAG and e‐cigarette devices in toxicological tests, we previously showed similar major constituent concentrations, selected carbonyls, and aerosol/particle size distributions between aerosols generated by a CAG and the commercial cig‐a‐like electronic nicotine delivery system products (Werley, Miller, et al., 2016; Zhang et al., 2021).

The aerosol/particle sizes can differ among various e‐cigarette products and are affected by the puffing regimen, power setting, and coil resistance of the device (Floyd et al., 2018; Mikheev et al., 2016). The aerosol/particle size distribution of the flavor and non‐flavor containing e‐vapor aerosols produced in this study was uniform and closely matched the aerosol size (0.9–1.2 μm) from cartridge based e‐cigarettes (Oldham et al., 2018; Werley, Miller, et al., 2016). Due to the mass captured on the cascade impactor, the submicron particles (96–175 nm) reported in selected e‐cigarette aerosols (Mikheev et al., 2016) were either absent or present in minute amounts in the e‐vapor aerosols of this study. Overall, the aerosol/particle sizes in the test atmosphere were within the expected and respirable ranges for efficient lung deposition in rodents (Asgharian et al., 2014; OECD, 2018a). The presence of e‐vapor constituents in the vapor phase is analyte specific due to the unique vapor pressure of each analyte (Pankow et al., 2018). At the measured TPM concentrations, the TPM collection efficiency was estimated to be 55–70% (Table S15). Hence, between 30 and 45% of the generated aerosol mass was either deposited or lost in the aerosol generation and exposure apparatus or was in the vapor phase and not collected on the Cambridge filter. Consistent with the anticipated higher TPM loss during particle size measurement (Kwon et al., 2003; Marple et al., 1991; Mitchell et al., 1988), the TPM yield captured by the cascade impactor was approximately 20–25% of the nominal TPM concentrations (Table S16). Hence, approximately 75–80% of the generated aerosol was either deposited or lost, not measured quantitatively or was in the vapor phase. Therefore, based on a conservative estimate, approximately 75–80% of the generated aerosol was in the vapor phase. The efficiency and dosimetry of vapor uptake at the respiratory tract organs is influenced by the blood/tissue solubility and reactivity of the individual constituents (Asgharian et al., 2012; Dahl et al., 1991; Morris & Hubbs, 2008). Given these variables, the particulate phase with MMAD <2 μm is the main phase to consider for lung delivery and lung pathology, while vapor phase constituents may exhibit variable effects due to loss through exhalation or deposition in multiple locations of the respiratory tract organs.

The potential impact of the various device setups and puffing regimens of different e‐cigarette devices on aerosol composition and toxicological outcomes are beyond the scope of this range‐finding study. In general, higher power settings as well as higher puffing duration and frequencies are associated with high heating coil temperature and, hence, more carbonyl and toxicant production. Under extreme high voltage conditions during the vaping process, “dry puffing” resulted in significant levels of toxicants (e.g., carbonyls) (Margham et al., 2016; Thomson & Lewis, 2015) and nanoparticles (11–25 nm) (Mikheev et al., 2016). Alternate products of formaldehyde (e.g., formaldehyde hemiacetal) may also be formed during operation of e‐cigarette devices at high power (Jensen et al., 2015). High levels of carbonyls and formation of formaldehyde hemiacetal and nanoparticles are not likely to be present in CAG‐generated e‐vapor aerosols because a constant temperature of 250°C and flow of liquid formulation were maintained during aerosol generation. This is also supported by the very low concentrations of formaldehyde and other carbonyls detected in e‐vapor aerosols compared to CS.

The concentrations of carbonyls measured in 3R4F CS were higher than those in the PG/VG/N aerosols at matching nicotine concentrations, which was consistent with previous descriptions of CS chemistry (Roemer et al., 2012) and e‐vapor aerosols produced by the CAG (Szostak et al., 2020; Werley, Miller, et al., 2016). The presence of small concentrations of formaldehyde in the PG/VG/N and PG/VG/N/F aerosols confirmed the production of low levels of the carbonyl compounds as a result of thermal degradation of glycols and glycerol during aerosol generation (Kosmider et al., 2014; Laino et al., 2011). The higher‐than‐expected concentrations of acetaldehyde and crotonaldehyde in the PG/VG/N/F (flavor‐containing) e‐vapor aerosols were likely due to an artifact of the capturing method, as the method would not differentiate the acetaldehyde that was formed from the hydrolyzed added flavor ingredient 1,1‐diethoxyethane from that directly formed by thermal degradation of the carrier (Zhang, 2019. The higher crotonaldehyde concentration in the PG/VG/N/F (flavor‐containing) aerosol than in the PG/VG/N (non‐flavor) e‐vapor aerosol is due to the formation of crotonaldehyde from acetaldehyde by aldol condensation (Conklin et al., 2018).

In this study, e‐vapor and smoke aerosols were consistently generated and delivered to the inhalation chambers. On the basis of the flavor concentrations in the aerosol of the PG/VG/N/F‐H (high flavor) exposure groups, the yields (representation of the transfer and trapping efficiencies) of the flavor ingredients were approximately 55%, 64%, 53%, 60%, 47%, and 67% for 2‐methoxy‐4‐methylphenol, citronellol, ethyl maltol, methyl anthranilate, eugenyl acetate, and triethyl citrate, respectively, in comparison to theoretical (considering 100% transfer/yield) aerosol concentrations. For the other detected flavors, the yields (trapping + transfer efficiencies) were 12%–87% (Table S17 and Zhang, 2019). Aerosolization or transfer efficiencies for the flavors were ranging 58–100% (Table S17). The aerosol yields obtained in this study were within the expected efficiency of aerosolization and the extent of deposition loss in the exposure system (Table S17 and Zhang, 2019). The yields of these flavors are similar to those of nicotine and TPM, which were approximately 57% and 55%, respectively (Asgharian et al., 2014; OECD, 2018a).

The recovery yields of total nicotine metabolites and flavor metabolites in urine in the present study are consistent with the presence of nicotine and/or flavor ingredients in the respective CS and PG/VG/N aerosols, confirming acceptable exposure and aerosol uptake levels. Mice in the male PG/VG/N/F (flavor with nicotine) group (but not those in the female group) excreted slightly higher levels of total urine nicotine metabolites than the mice in the PG/VG/N (nicotine alone) group, which was likely incidental, given the observed higher urine output in the male PG/VG/N/F (flavor with nicotine) group. While the amount of total nicotine metabolites in the female PG/VG/N/F (flavor with nicotine) group was slightly lower than that in the PG/VG/N (nicotine alone) group, the difference was not statistically significant and may be the result of the slightly lower urine output in the female PG/VG/N/F group. Therefore, it is likely that the high flavor content did not affect the overall nicotine uptake in the high PG/VG/N/F (flavor with nicotine) groups.

In this study, no acute or severe toxicity or significant weight loss was observed in PG/VG/N (non‐flavor) aerosol‐exposed mice. The tremor and weight loss observed during acclimatization to the aerosol exposure were transient and are within the expected responses to nicotine‐containing aerosols (Chowdhury, 1990; Phillips, Esposito, et al., 2015; Wong et al., 2020). The PG/VG/N/F (flavor with nicotine) groups showed a slightly higher frequency of post‐exposure tremor (in male and female mice) and lower body weight (male) than the PG/VG/N (nicotine alone) group. As tremors were frequently observed during the aerosol adaptation period and only a few mice were impacted by this finding, we could not rule out the impact of nicotine or flavor on the respiratory response of the mice, nicotine metabolism, and/or stress responses during the early phase of the study.

Animal handling and nicotine exposure are known to affect various stress regulation systems, resulting in multiple effects, including changes in leukocyte counts (Everds et al., 2013; Faraday et al., 2005; Matta et al., 1998; Reiche et al., 2004; Rhodes et al., 2001; Sopori, 2002). The effects of nicotine and flavored e‐vapor aerosol exposure on lymphocyte counts and spleen and thymus weights are likely secondary responses to stress. Similar changes have been observed in previous CS and nicotine‐exposure studies (Oviedo et al., 2016; Phillips et al., 2017; Phillips, Veljkovic, et al., 2015; Wong et al., 2016, 2020), which were likely attributable to increased stress hormone levels (Stinn, Buettner, et al., 2013; Sundar et al., 2014). Flavor ingredients did not impact general stress responses in previous inhalation studies (Ho et al., 2020; Kumar et al., 2021; Lee et al., 2018; Szostak et al., 2020). The animal strain, flavor composition, and (very high) flavor concentrations used in this study may have an impact on the biological outcomes. Therefore, we cannot rule out the effect of nicotine or flavor ingredients on general stress responses for a subsequent long‐term inhalation study.

Other organ weight changes seen in this study—including the higher adrenal gland weights in the female sham mice than in the male sham mice—are consistent with sex‐dependent growth effects on the organs (Biedermann et al., 2014). The lower adrenal gland weight observed in the CS group was consistent with the findings of a previous studies (Kumar et al., 2021; Wong et al., 2020), even though no histopathological findings of biological significance were found. Marginal reductions in ovary weights were previously observed in response to chronic exposure to CS and aerosol of a heated tobacco product (Wong et al., 2020). Although CS and nicotine exposure were shown to negatively affect the maturation and/or number of follicles in the ovaries (Iranloye & Bolarinwa, 2009; Tuttle et al., 2009), no histopathological changes were found to correlate in A/J mice (Wong et al., 2020). While minor flavor‐ or nicotine‐dependent changes in ovary weight were noted in this sub‐acute exposure study, any potential pathological impact from ovary weight changes due to chronic flavor and/or nicotine exposure requires further histopathological evaluation. The increased alanine aminotransferase activity (female mice only) and lower serum protein concentrations we observed in the PG/VG/N/F (flavor with nicotine) groups were subtle and consistent with the findings of previous nicotine exposure studies (Ho et al., 2020; Phillips et al., 2017).

The presence of high levels of flavors may contribute to irritation and inflammation of respiratory airways (Bengalli et al., 2017; Erythropel, Jabba, et al., 2019; Gerloff et al., 2017; Leigh et al., 2016). Components of e‐vapor aerosols, such as saline (Phillips, Esposito, et al., 2015), PG (Werley et al., 2011), and VG (Renne et al., 1992), can also induce mild adaptive changes in upper respiratory tract organs. In this study, there was minimal lung inflammation (on the basis of BALF analysis and histopathological evaluation of the lungs) in response to nicotine and flavor e‐vapor aerosol exposure. The observed marginally lower BALF neutrophil and FLC counts did not have corresponding lower blood neutrophil counts in the e‐vapor aerosol‐exposed groups. Potential technical variation in BALF recovery could explain the lower cell and protein/analyte recovery in the female PG/VG/N/F (nicotine with flavor) group. The minimal lung inflammation in the e‐vapor groups is supported by the results of other flavor‐containing e‐vapor aerosol exposure studies (Ho et al., 2020; Larcombe et al., 2017; Olfert et al., 2018; Werley, Kirkpatrick, et al., 2016; Wong et al., 2021). The robustness of the increased total and differential leukocyte counts present in BALF supporting increased lung inflammation was substantiated by the corresponding changes in the concentration of inflammatory mediators, total protein content, and increased histopathology severity scores. The higher total and differential FLC counts in the CS group are also consistent with the findings of previous inhalation studies in mice (Phillips et al., 2016; Phillips, Veljkovic, et al., 2015; Stinn, Buettner, et al., 2013). The absence of lung inflammation in the present 5‐week study indicated that the tested e‐liquid constituents and thermal treatment played a minimal role in contributing to lung inflammation and lung injury. While emphysema was minimal only in the CS group, the impact of e‐vapor aerosol exposure on emphysematous and lung function changes will be explored in future sub‐chronic exposure studies.

With regard to irritation in the upper respiratory tract, we observed a minimal exposure effect in the nasal epithelia of the PG/VG/N/F (flavor with nicotine) e‐vapor aerosol group. The PG/VG/N (nicotine alone) and PG/VG/N/F (flavor with nicotine) groups did not show most of the adaptive laryngeal changes typical of CS exposure. Where some effects were observed, the adaptive changes were minimal to mild in the PG/VG/N/F (flavor with nicotine) groups, although there was a concentration‐dependence trend to these changes. The changes in the larynx were, in general, more severe in the PG/VG/N/F (flavor) groups than in the PG/VG/N (non‐flavor) groups, but they were consistently less severe than the changes in the CS group. This is consistent with the mild irritation effects of nicotine and/or flavors in the aerosol (Ho et al., 2020; Phillips et al., 2017) and has also been reported in response to PG (Werley et al., 2011) and VG (Renne et al., 1992) exposure. The larynx is the most sensitive site for detecting the irritation‐based effects of inhaled aerosol particles in the respiratory tract (Burger et al., 1989; Osimitz et al., 2007). Because we tested a greater number of flavor compounds at higher concentrations than in the study by Ho et al. (2020) (8.8% w/v), the mild irritation effects observed here are within the limits of expectation in the inhalation study. The high flavor concentration tested in the present study did not cause severe sub‐acute toxicity or respiratory tract irritation/inflammation and, therefore, was considered suitable for use in future chronic inhalation studies in A/J mice.

This study has several limitations. First, the 5‐week exposure only allows immediate and sub‐acute responses to be evaluated. While lung inflammation was readily observed following CS exposure, any potential impact on emphysema, lung tumor development, and progression of adaptive changes in the upper respiratory tract organs will require longer exposure (Wong et al., 2020). However, the absence of severe toxicity following nicotine and flavor inhalation exposure suggests a potentially lower impact of nicotine and flavor exposure compared with CS exposure. Therefore, the current exposure regimen can be used in a future chronic inhalation study. Second, due to anticipated nicotine responses in past studies (Oviedo et al., 2016; Phillips et al., 2017; Phillips, Veljkovic, et al., 2015; Wong et al., 2016, 2020), the flavor alone group was omitted. From the results of exposure‐related stress and larynx histopathology assessments in the nicotine and flavor (PG/VG/N/F) groups, the possibility of toxicity due to flavor alone or synergistic effects with nicotine will also be explored in a future study.

## 5 CONCLUSION

Based on read‐across and flavor toolbox concepts, a total of 38 FGRs were selected from a wider set of 245 flavor ingredients and assessed in this 5‐week study in A/J mice to characterize the subacute toxicity after repeated inhalation exposure to the flavor aerosols. The PG/VG/N/F (with flavor mixture) test atmosphere was well tolerated by the mice. In contrast to CS exposure, exposure to flavor and non‐flavor PG/VG/N aerosols did not cause lung inflammation. Most of the CS exposure‐related effects on respiratory tract organs were not seen with flavor or non‐flavor PG/VG/N aerosol exposure. When observed, the changes in nasal and laryngeal epithelia were significantly less severe in the flavor and non‐flavor PG/VG/N groups than in the CS group. The adaptive changes observed in the larynx were, in general, more severe in the flavor PG/VG/N groups than in the non‐flavor PG/VG/N groups, which suggests a low level of irritation due to nicotine and/or flavors in the aerosols. Typical nicotine exposure‐ and stress‐related responses such as lower lymphocyte counts and thymus weight were observed in both the CS‐ and flavor PG/VG/N aerosol‐exposed groups. The lower spleen weights seen in the flavor PG/VG/N groups than in the non‐flavor PG/VG/N groups suggest exposure‐related stress due to nicotine and/or flavors in the aerosols. The high flavor concentration (18.6%) did not cause severe toxicity upon delivery via the inhalation route and can be considered suitable for use in the future chronic inhalation studies in A/J mice. The concept of the read‐across and chemical toolbox approach may be broadly applied to the toxicological testing of other chemical compounds.

## CONFLICT OF INTEREST

The flavor groups representatives (FGR) used in this study were developed on the basis of individual flavor chemicals used at PMI and ALCS. All authors are (or were) employees of PMI or ALCS or worked with PMI under contractual agreement.

## Supporting information


**Figure S1** Plot of aerosol/particle size distribution

**Figure S2** Terminal body weight

**Figure S3** Results of BALF analysis

**Figure S4** Representative histology images of the lung, larynx and nose sections

**Figure S4** Supporting information

**Figure S4** Supporting information

**Figure S5** Absolute organ weights

**Figure S6** Organ weights relative to bodyweight.

**Figure S7** Red blood cell parameters

**Table S1** Experimental groups and study endpoints.
**Table S2** Mass compositions of the PG/VG/N and PG/VG/N/F inhalation formulations
**Table S3** Ingredients of the flavor preblends
**Table S4** Characterization of the inhalation formulations
**Table S5** Results of pH measurement as well as microbial and endotoxin content in the inhalation formulations
**Table S6** Nicotine, TPM, PG, VG, and carbonyl concentrations in the exposure chambers
**Table S7** Investigation of the contribution of 1,1‐diethoxyethane to acetaldehyde detection
**Table S8** Aerosol/particle size distribution
**Table S9** Clinical observations post exposure
**Table S10** Erythrocyte count and red blood cell indices in whole blood
**Table S11** Leukocyte counts in whole blood
**Table S12** Serum clinical chemistry results
**Table S13** Analysis of BALF analytes
**Table S14** Estimation of delivered dose and human equivalent dose
**Table S15** Aerosol TPM yield
**Table S16** TPM yield captured at PIXE impactor
**Table S17** Aerosol trapping and transfer rates for flavor compounds

## Data Availability

Datasets, additional data visualizations and detailed protocols are available on the INTERVALS™ platform at https://doi.org/10.26126/intervals.lurrz2.1.
