# Supplementary figures and images for: Assessment of inhalation toxicity of cigarette smoke and aerosols from flavor mixtures: 5‐week study in A/J mice
Source: J Appl Toxicol. 2022 Jun 8;42(10):1701–22. doi: 10.1002/jat.4338 (PMC9545811; doi:10.1002/jat.4338)

a PG/VG/N e-vapor aerosol

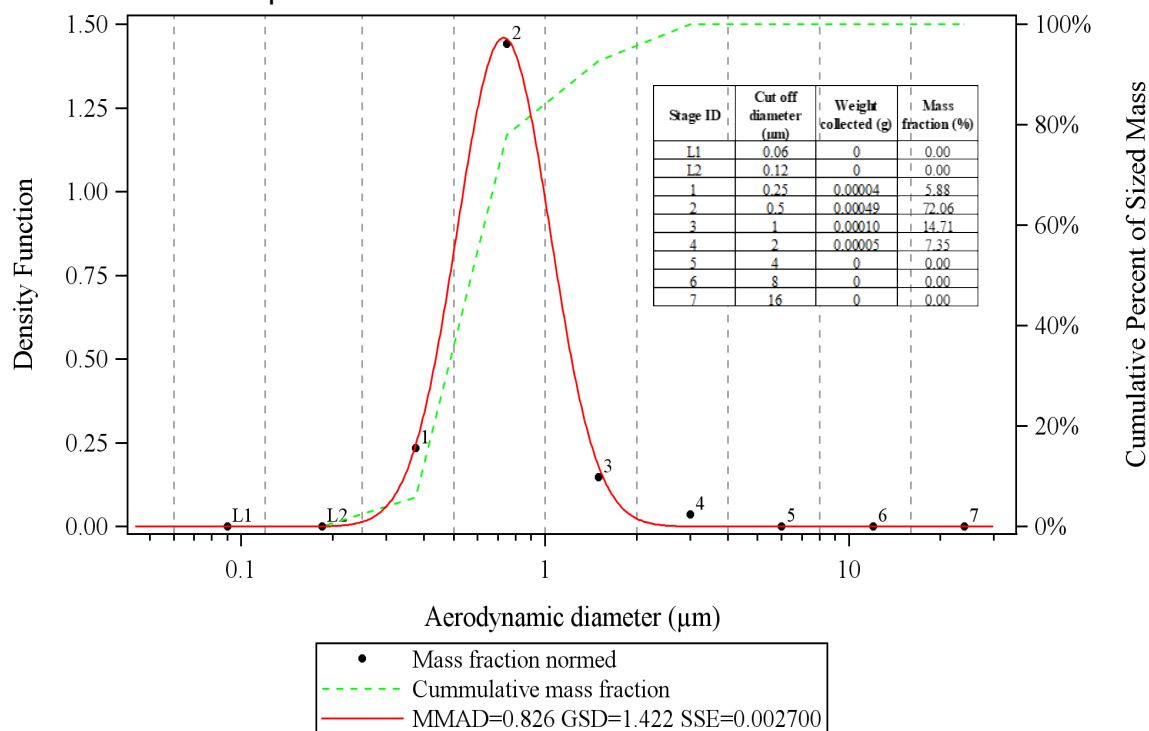

b PG/VG/N/F-H e-vapor aerosol

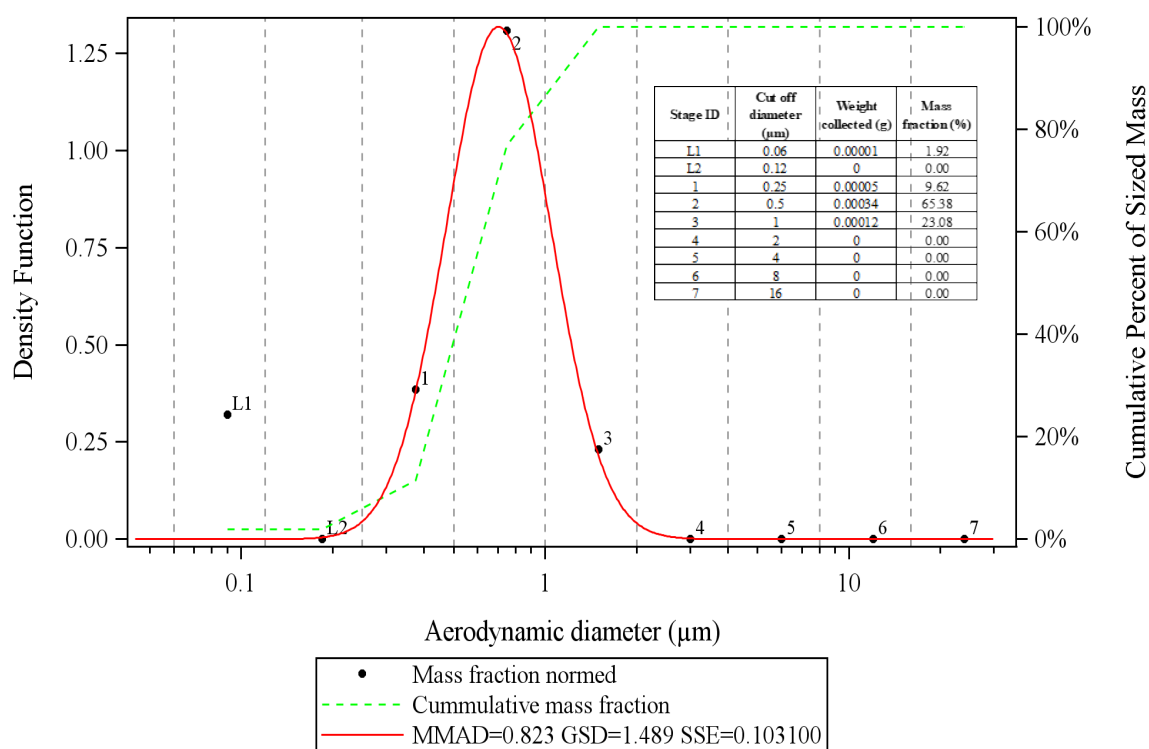

Supplement: Supplementary file 1 — Figure S1 Plot of aerosol/particle size distribution [file JAT-42-1701-s010.pdf]

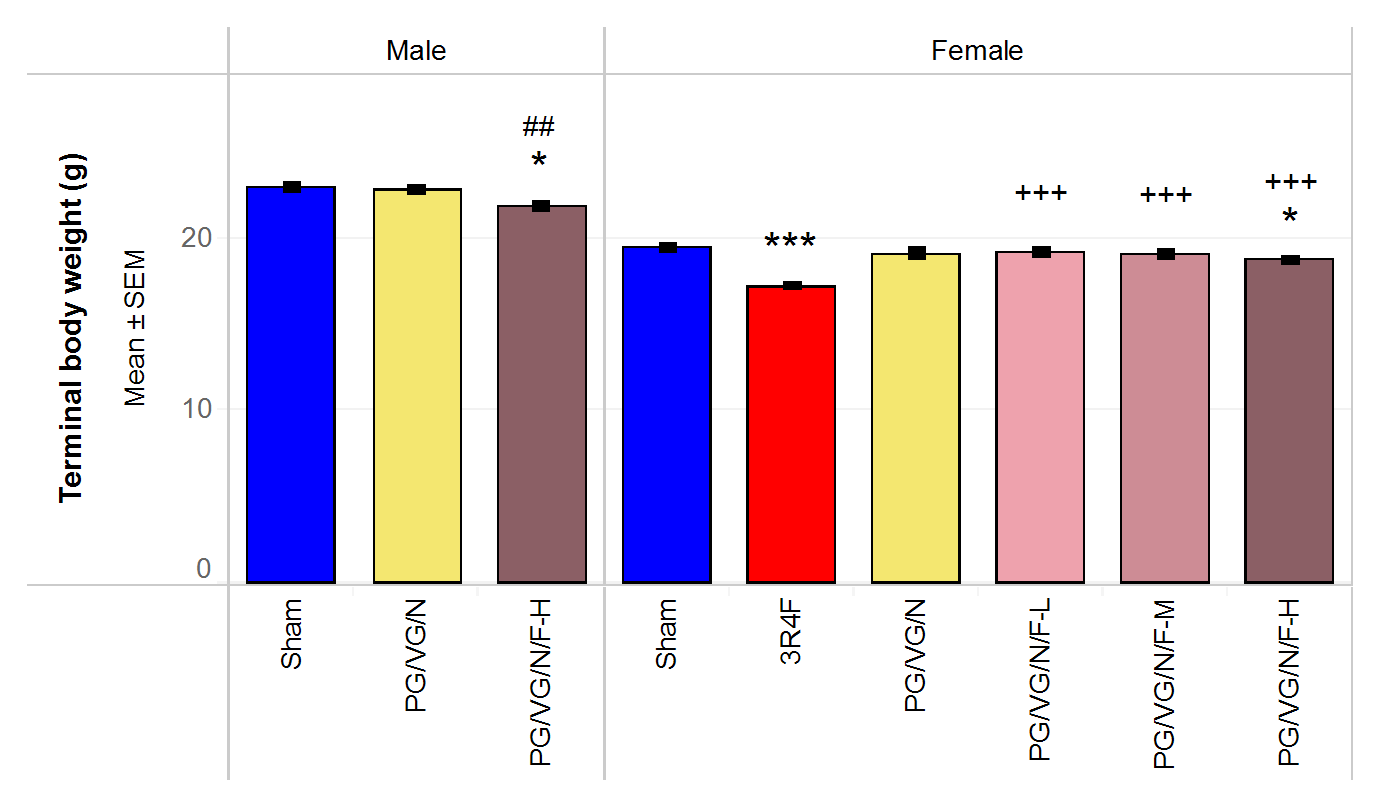

Supplement: Supplementary file 2 — Figure S2 Terminal body weight [file JAT-42-1701-s004.tif]

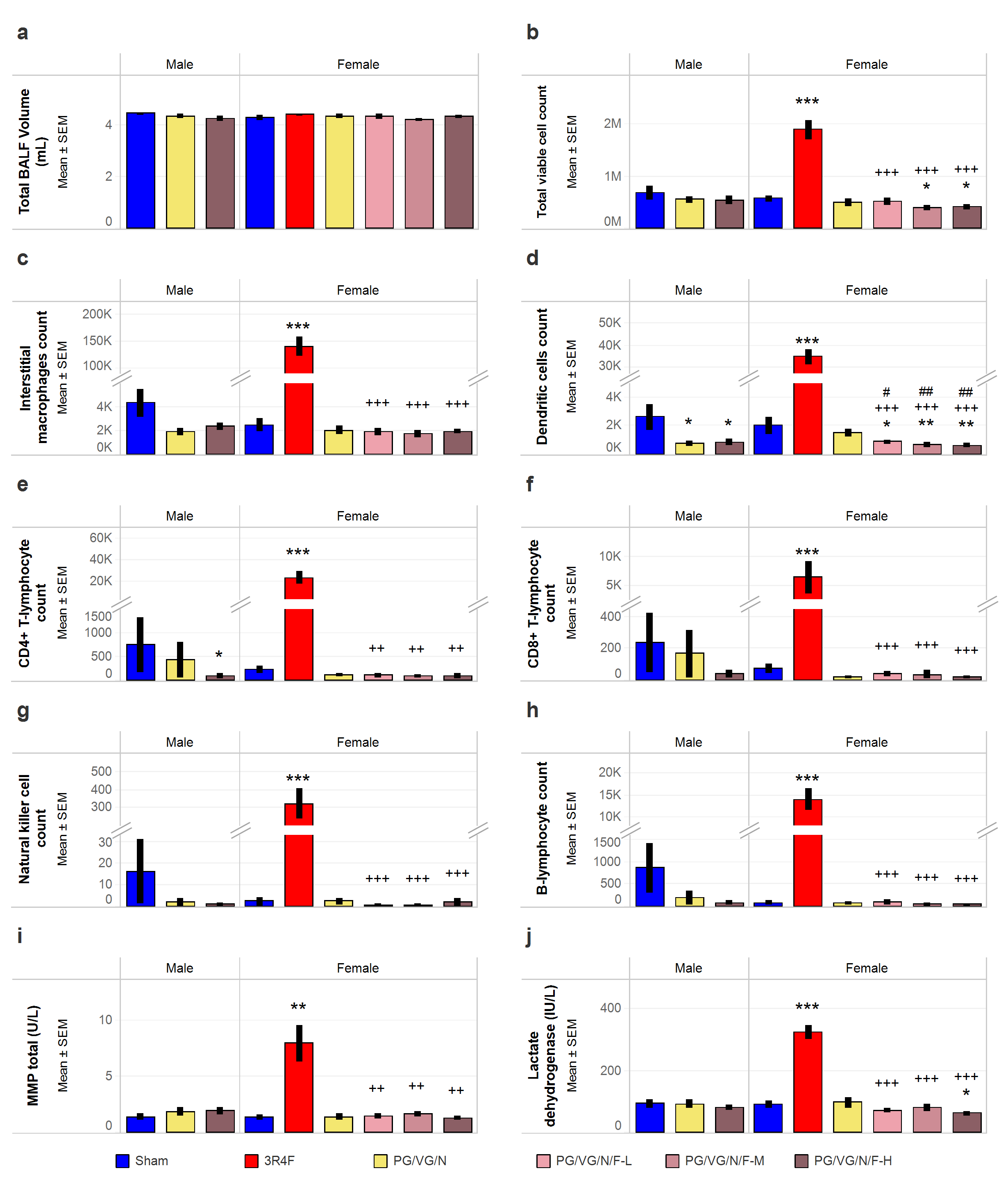

Supplement: Supplementary file 3 — Figure S3 Results of BALF analysis [file JAT-42-1701-s006.tif]

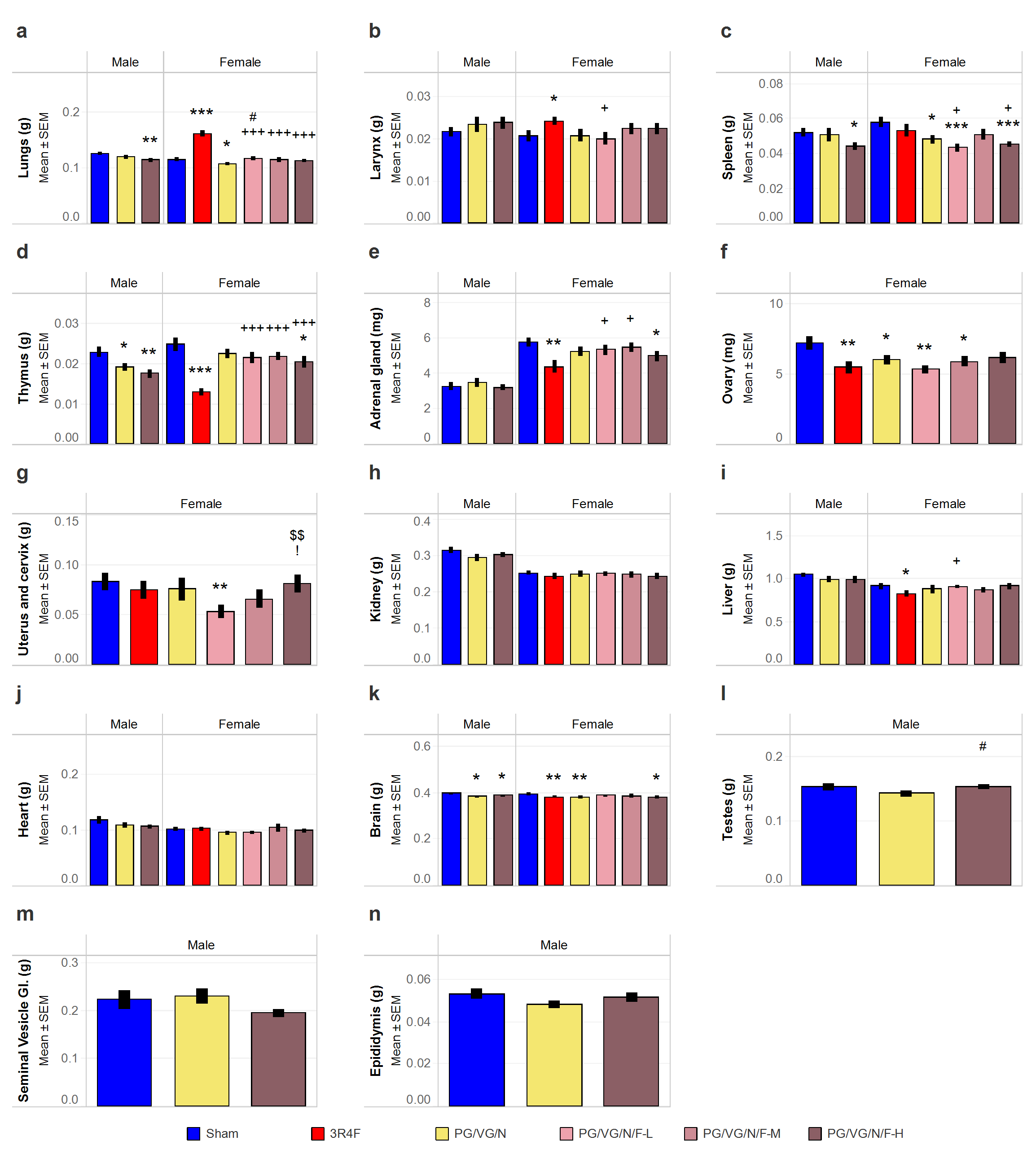

Supplement: Supplementary file 7 — Figure S5 Absolute organ weights [file JAT-42-1701-s008.tif]

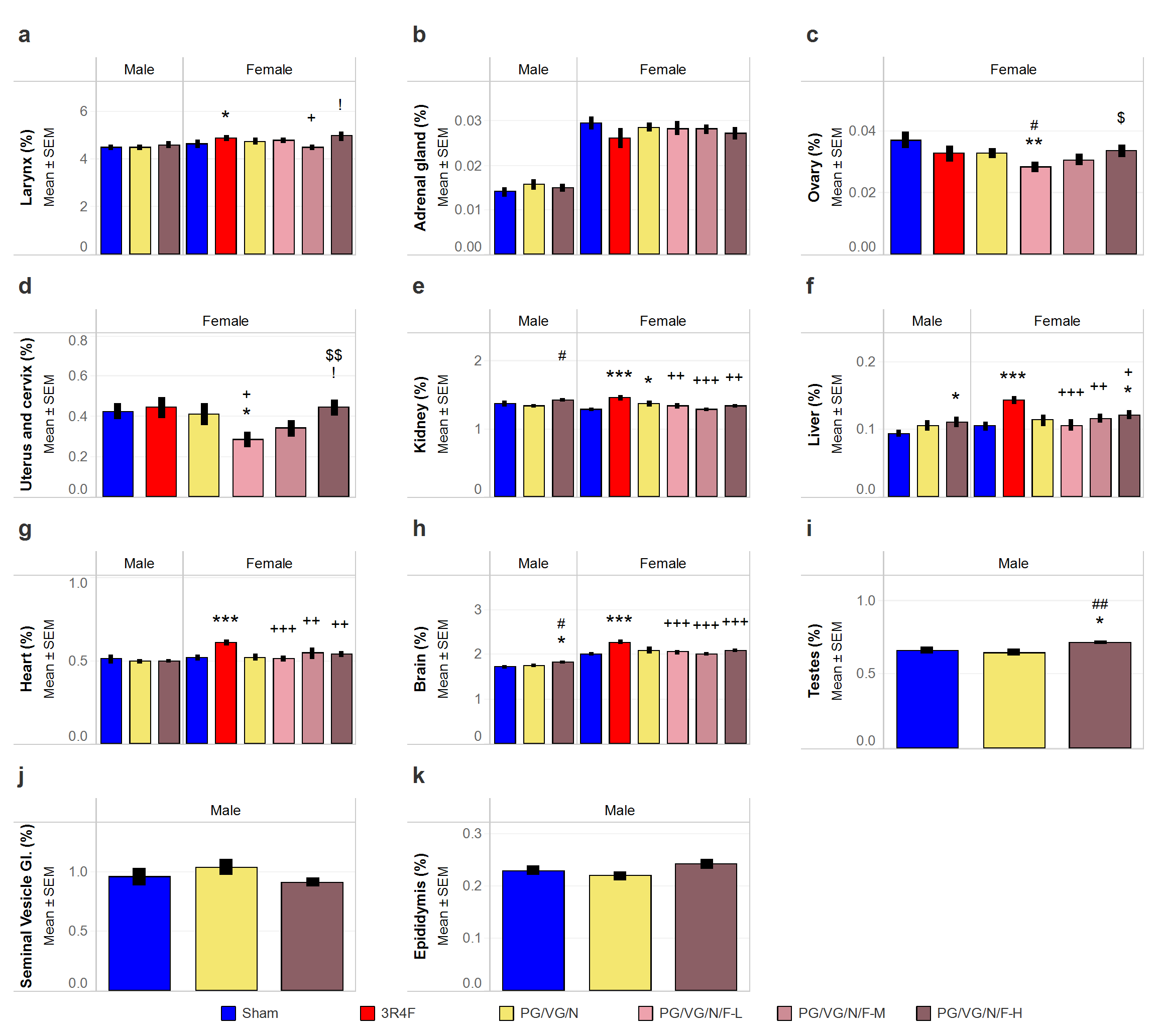

Supplement: Supplementary file 8 — Figure S6 Organ weights relative to bodyweight. [file JAT-42-1701-s003.tif]

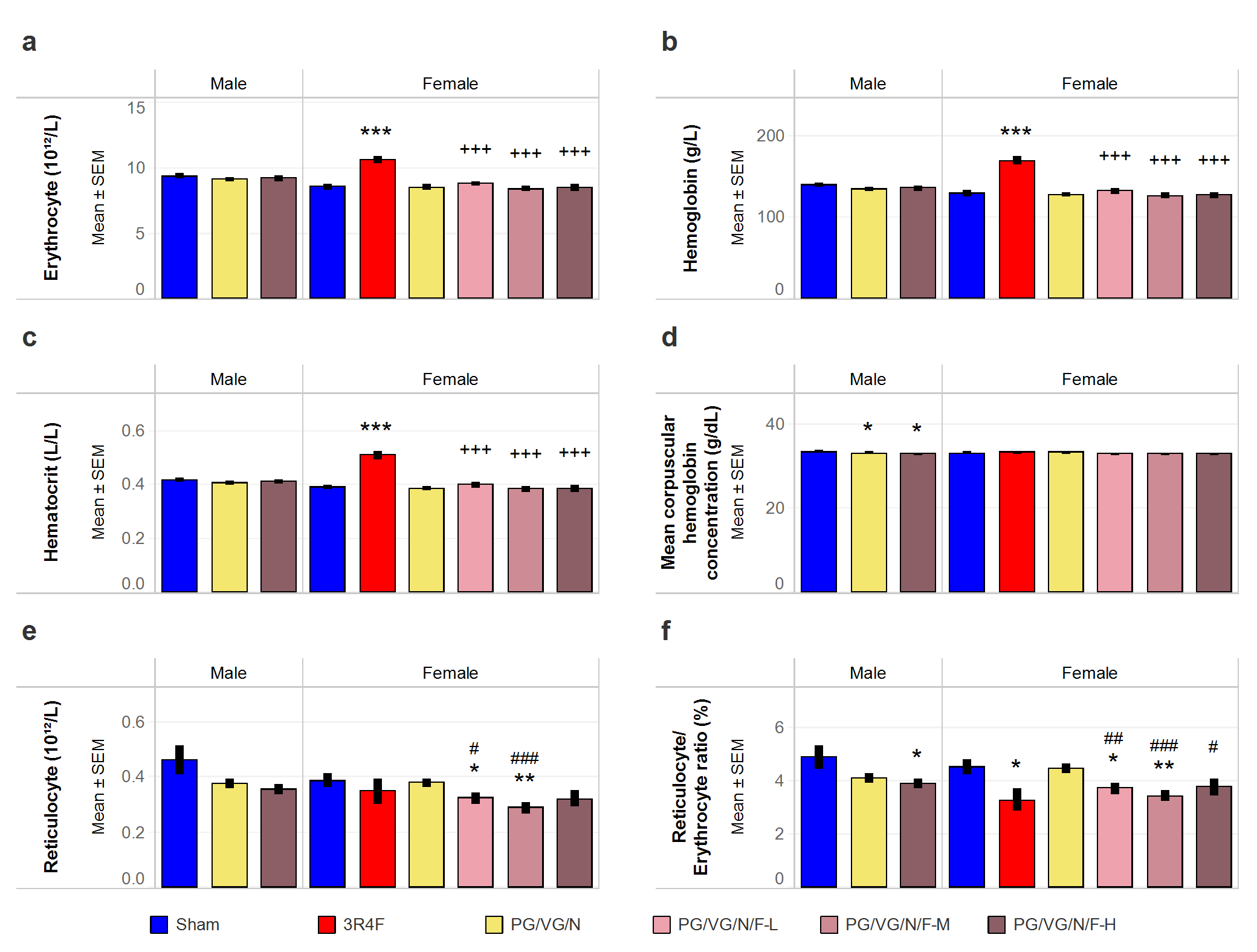

Supplement: Supplementary file 9 — Figure S7 Red blood cell parameters [file JAT-42-1701-s009.tif]
