# Supplementary material for: Assessment of inhalation toxicity of cigarette smoke and aerosols from flavor mixtures: 5‐week study in A/J mice
Source: J Appl Toxicol. 2022 Jun 8;42(10):1701–22. doi: 10.1002/jat.4338 (PMC9545811; doi:10.1002/jat.4338)
Supplement: Supplementary file 10 — Table S1 Experimental groups and study endpoints. Table S2 Mass compositions of the PG/VG/N and PG/VG/N/F inhalation formulations Table S3 Ingredients of the flavor preblends Table S4 Characterization of the inhalation formulations Table S5 Results of pH measurement as well as microbial and endotoxin content in the inhalation formulations Table S6 Nicotine, TPM, PG, VG, and carbonyl concentrations in the exposure chambers Table S7 Investigation of the contribution of 1,1‐diethoxyethane to acetaldehyde detection Table S8 Aerosol/particle size distribution Table S9 Clinical observations post exposure Table S10 Erythrocyte count and red blood cell indices in whole blood Table S11 Leukocyte counts in whole blood Table S12 Serum clinical chemistry results Table S13 Analysis of BALF analytes Table S14 Estimation of delivered dose and human equivalent dose Table S15 Aerosol TPM yield Table S16 TPM yield captured at PIXE impactor Table S17 Aerosol trapping and transfer rates for flavor compounds [file JAT-42-1701-s007.docx]

**Inhalation toxicity assessment of aerosols from flavor mixtures and cigarette smoke: A 5-week study in A/J mice**

Wong ET^1*^, Luettich K^2^, Cammack L^1^, Chua C S^1^, Sciuscio D^2^, Merg C^2^, Corciulo M^2^, Piault R^2^, Kumar A^3^, Smith C^3^, Leroy P^2^, Moine, F^2^, Anneke Glabasnia^2^, Pierrick Diana^2^, Chia Cecilia^1^, Tung Ching Keong^1^, Ivanov N^2^, Hoeng J^2^, Peitsch, M^2^, Lee K M^3^, Vanscheeuwijck P^2^

^1^*PMI R&D, Philip Morris International Research Laboratories Pte Ltd, Singapore*

^2^*PMI R&D, Philip Morris Products S.A., Neuchâtel, Switzerland*

*^3^Altria Client Services LLC, Richmond, VA, UA*

*Corresponding author: Ee Tsin Wong (email: eetsin.wong@pmi.com)

**Supporting Information – Material and Methods**

**Study design**

Table S1 Experimental groups and study endpoints.

|  |  |  | Number of animals allocated | | |
| --- | --- | --- | --- | --- | --- |
| Sex | Group name/exposure | Group code | Histopathology | BALF | Total |
| Female | Sham | 101 | 11 | 10 | 21 |
| Female | 3R4F | 201 | 11 | 10 | 21 |
| Female | PG/VG/N | 501 | 11 | 10 | 21 |
| Female | PG/VG/N/F-L | 601 | 11 | 10 | 21 |
| Female | PG/VG/N/F-M | 701 | 11 | 10 | 21 |
| Female | PG/VG/N/F-H | 801 | 11 | 10 | 21 |
| Male | Sham | 101 | 11 | 10 | 21 |
| Male | PG/VG/N | 501 | 11 | 10 | 21 |
| Male | PG/VG/N/F-H | 801 | 11 | 10 | 21 |

PG, propylene glycol; VG, vegetable glycerol; N, nicotine; F, flavors; H, high; M, medium; L, low; BALF, bronchoalveolar lavage fluid.

**Preparation of the test and reference item inhalation formulations**

The e-liquid formulations (or inhalation formulations) were prepared by mixing PG, VG, nicotine, and flavors ([Sciuscio et al., Accepted](#_ENREF_6)) at defined mass compositions (Table S2). PG, VG, and nicotine were of United States Pharmacopeia (USP) standard (Sigma-Aldrich Pte Ltd, Singapore). The flavor mixtures were provided in the form of concentrated blended flavors (a total of 6 preblends) composed of food-grade flavor compounds by MANE (Jakarta, Indonesia), and mixed in defined mass compositions (Table S3) to the final formulations. In relation to the high flavor formulation, preblends 1a, 1b, 1c, 2, 3 and 4 were prepared as concentrates of 20x, 10x, 5x, 10x and 20x respectively. Accuracy of the preparation was based on the certificate of analysis for preblends and weighing records for formulations during preparation. The preblends and inhalation formulations were stored away from light at a controlled temperature of 2–8℃. The preblends were used within 35 days from the manufacturing date ([Smith, 2019](#_ENREF_8)). The flavor-containing inhalation formulations were used within 3 days of preparation, while the non-flavor formulation was used within 21 days of preparation.

Table S2 Mass compositions of the PG/VG/N and PG/VG/N/F inhalation formulations

|  | Component (g/100 g) | | | | | |
| --- | --- | --- | --- | --- | --- | --- |
| Inhalation formulation | PG | VG | Nicotine | Water | Ethanol | Flavor |
| PG/VG/N | 71.7 | 17.9 | 2.0 | 5.8 | 2.5 | 0.0 |
| PG/VG/N/F High | 56.9 | 14.2 | 2.0 | 5.8 | 2.5 | 18.6 |
| PG/VG/N/F Med | 64.3 | 16.1 | 2.0 | 5.8 | 2.5 | 9.3 |
| PG/VG/N/F Low | 68.0 | 17.0 | 2.0 | 5.8 | 2.5 | 4.6 |

PG, propylene glycol; VG, vegetable glycerin; N, nicotine; F, flavor; Med, medium.

Table S3 Ingredients of the flavor preblends

| **Preblend** | **Component** | **CAS number** | **Proportion in High flavor formulation (%, w/w)** |
| --- | --- | --- | --- |
| 1a | Propylene glycol | 57-55-6 | 5.0 |
|  | *para*-Cymene | 99-87-6 |  |
|  | 1-Penten-3-one | 1629-58-9 |  |
|  | Isopulegol | 89-79-2 |  |
|  | Isobutyraldehyde | 78-84-2 |  |
|  | Citronellol, D-L- | 106-22-9 |  |
|  | Ethyl lactate | 97-64-3 |  |
|  | *cis*-3-Hexenol | 928-96-1 |  |
|  | Acetal | 105-57-7 |  |
|  | 2-Methyl-4-phenyl-2-butanol | 103-5-9 |  |
| 1b | Ethanol | 64-17-5 | 10.0 |
|  | Ambrox (cetalox^®^) | 3738-00-9 |  |
|  | *para*-Dimethoxybenzene | 150-78-7 |  |
|  | Propylene glycol | 57-55-6 |  |
|  | *alpha*-Damascone | 43052-87-5 |  |
|  | Ethyl-2-methylbutyrate | 7452-79-1 |  |
|  | Acetanisole | 100-06-1 |  |
|  | Eugenyl acetate | 93-28-7 |  |
| 1c | Ethanol | 64-17-5 | 20.0 |
|  | *alpha*-Pinene | 80-56-8 |  |
|  | *delta*-Nonalactone | 3301-94-8 |  |
|  | Isoamyl alcohol | 123-51-3 |  |
|  | Benzyl alcohol | 100-51-6 |  |
|  | 2-Methoxy-4-methylphenol | 93-51-6 |  |
|  | Ethyl vanillin | 121-32-4 |  |
|  | Propylene glycol | 57-55-6 |  |
| 2 | Ethanol | 64-17-5 | 10.0 |
|  | Methyl cinnamate | 103-26-4 |  |
|  | Propylene glycol | 57-55-6 |  |
|  | Sihydroactinidiolide | 15356-74-8 |  |
|  | Furaneol | 3658-77-3 |  |
|  | Ethyl maltol | 11/8/4940 |  |
|  | (*e*,*z*)-2,6-Nonadienal | 557-48-2 |  |
|  | Piperitone | 89-81-6 |  |
|  | Ketoisophorone | 1125-21-9 |  |
| 3 | Propylene glycol | 57-55-6 | 5.0 |
|  | 3-Methyl-2,4-nonanedione | 113486-29-6 |  |
|  | Triethyl citrate | 77-93-0 |  |
| 4 | Propylene glycol | 57-55-6 | 5.0 |
|  | 2-Acetylpyrrole | 1072-83-9 |  |
|  | 2-Acetylthiazole | 24295-03-2 |  |
|  | 3-Ethylpyridine | 536-78-7 |  |
|  | *para*-Mentha-8-thiol-3-one | 38462-22-5 |  |
|  | Methyl anthranilate | 134-20-3 |  |
|  | 2,5-Dimethylpyrazine | 123-32-0 |  |

CAS, chemical abstracts service.

**Characterization of the test and reference item inhalation formulations**

PG, VG, nicotine, and one flavor representative of each preblend were analyzed once per 2 weeks for one preparation of each of the inhalation formulations. A total of six flavor compounds were analyzed: citronellol is the representative of preblend 1a, eugenyl acetate of preblend 1b, 2-methoxy-4-methylphenol of preblend 1c, ethyl maltol of preblend 2, triethyl citrate of preblend 3, and methyl anthranilate of preblend 4.. Analysis was performed by capillary gas chromatography (GC) (7890A/7890B series, Agilent Technologies, Santa Clara, CA, USA) with a DB-624UI column (Agilent Technologies) by using a flame ionization detector (FID) and *n*-heptadecane as internal standard. The microbial content in inhalation formulations was determined once per month by filtering the diluted solution (diluted 1:10 with phosphate buffered saline; Thermo Fisher Scientific, Waltham, MA, USA) through a 0.2-µm filter membrane (VWR, Radnor, PA, USA) before incubation on trypticase soy agar plates (bioMérieux, Marcy **l'Etoile**, France) at 32.5℃ (±2.5℃) for up to 3 days. The endotoxin content in inhalation formulations was determined by the bacterial endotoxins test method([USP41, 2018](#_ENREF_9)) by ALS Technichem (S) Pte. Ltd (Singapore). The pH of the inhalation formulation was measured once per 2 weeks by using a pH meter (Seven2Go^TM^ S2 pH meter; Mettler Toledo, Columbus, OH, USA) after dilution of 0.5 g into 25 mL of 1% sodium chloride solution (Sigma-Aldrich Pte Ltd). The results of these tests are shown in Supplementary File 2.

**Analysis of test atmospheres**

Total particulate matter (TPM) collected on Cambridge filter was quantified three times per exposure day, and carbon monoxide (CO) content was monitored continuously throughout the exposure ([Wong et al., 2020](#_ENREF_10)). The nominal concentrations of TPM for e-vapor aerosols were calculated based on CAG consumption rate (g/min) / total air dilution (L/min) x analyte content per gram of inhalation formulation (1 gram per gram). Absolute TPM yield was calculated based on the percentage of achieved TPM concentration divided by the nominal TPM concentration. Nicotine, PG, and VG concentrations in the test atmospheres were determined once per day for the sham groups and three times per exposure day for the 3R4F, PG/VG/N, and PG/VG/N/F groups. The concentrations of flavor compounds (citronellol, eugenyl acetate, 2-methoxy-4-methylphenol, ethyl maltol, triethyl citrate, and methyl anthranilate, each representative of one preblend) were determined once every 2 weeks. Aerosols were captured in 2-propanol-impregnated 3NT EXtrelut^®^ tubes (Merck Millipore, Burlington, MA, USA) and analyzed by GC-FID method as described for inhalation formulations. Carbonyl concentrations were determined once per study by reverse-phase high-performance liquid chromatography (HPLC; 1200 series, Agilent Technologies, CA, USA) with a Phenomenex Synergi column (Phenomenex, Torrance, CA, USA) and mass spectrometer detection (ABSciex QTRAP 4000) of the 2,4-dinitrophenylhydrazine (DNPH (from PanReac AppliChem, Castellar del Vallès, Barcelona, Spain) derivatives; d3 forms of individual carbonyl–DNPH derivatives (Toronto Research Chemicals, North York, Ontario, Canada) were used as internal standards. Using the DNPH capturing method, carbonyls in both liquid and vapor phases was trapped. The TPM concentration captured on the PIXE cascade impactor (for determining the particle size distribution) was calculated based on the total weight collected at a 1 L/min sampling flow and 2 minute sampling time. The TPM yield of the PIXE cascade impactor was calculated by expressing the captured TPM concentration as a percentage of the nominal TPM concentration.

**Determination of trapping and transfer efficiencies for flavor compounds**

Trapping efficiencies of the Extrelut®NT 3 cartridges were determined by spiking the Extrelut cartridges with flavor solutions (high concentration), followed by flushing the cartridges for 30 min with conditioned air (22± 2° C, 60 ± 5% RH) at a flow rate of 0.7L/min. The Extrelut tubes were extracted with 20 mL isopropanol before gas chromatography-mass spectrometry (GC–MS) quantification. Trapping efficiencies were the ratios of the amount of quantified analyte with the amount of flavors dissolved directly in isopropanol. Trapping+transfer efficiencies were determined by passing aerosols (high flavor concentration) generated from the CAG at 0.7L/min to the Extrelut®NT 3 cartridges and expressing the ratio of the amount of quantified analyte with the amount of flavors dissolved directly in isopropanol. The efficiencies obtained from replicate results were analyzed using the Bayesian modelling. Transfer or aerosolization efficiencies are derived as trapping/(trapping+transfer) efficiencies. Flavor compounds were analyzed using GC (Agilent 7890A, Agilent Technologies) equipped with a J&W DB-624 ultra-inert column (Agilent Technologies) and detected using a 7200A Q-TOF mass spectrometer system (Agilent Technologies) operating in full scan mode at *m/z* values ranging from 22–500 using positive electron ionization.

**Analysis of eugenol and ethyl vanillic acid in urine**

Eugenol and ethyl vanillic acid were quantified by LC–TSQ-MS detection by using an HPLC system (HPLC; Dionex UltiMate3000, ThermoFisher, Waltham, MA, USA) with a Kinetex EVO C18 100Å or Kinetex biphenyl column (Phenomenex, Torrance, CA, USA) and mass spectrometer detection (ThermoFisher TSQ Altis^TM^); guaiacol (Sigma) and resveratrol (Sigma), respectively, were used as internal standards.

**Supporting Information – Results**

**Characterization of the inhalation formulations**

Analyses of the prepared inhalation formulations indicated accurate preparation, with mean PG, VG, and nicotine concentrations within ±10% of the target concentrations in the PG/VG/N as well as low, medium, and high flavor-containing (PG/VG/N/F) formulations (Table S4). The average concentrations of the six representative flavor compounds in the inhalation formulations were within 20% of the target concentrations, except in case of methyl anthranilate in the low flavor formulation, where it was present at +25% of the expected concentration. Because of the analytical limitation of a coeluting peak from eugenyl acetate during GC-FID analysis, the concentrations of methyl anthranilate might have been overestimated. The limited sensitivity and specificity of the GC-FID method are expected to impact the accuracy of quantification in the low flavor formulation more than the medium and high flavor formulations. Methyl anthranilate concentrations in the medium and high flavor formulations would be better representatives for confirming accurate preparation of the preblends and formulations. In general, the analytical results confirmed that the compositions of the inhalation formulations were within acceptable concentration tolerance ranges. The average pH of the PG/VG/N inhalation formulation was 9.5 (Table S5). Increasing concentrations of flavor compounds in the test formulations resulted in lower pH values — pH 8.6 in the low flavor formulation, pH 8.3 in the medium flavor formulation, and pH 7.9 in the high flavor formulation. The pH of the formulations was maintained above 7.5, as inhalation of acidic aerosol (<pH 4.0), but not alkaline aerosol, can compromise lung function and induce airway hyperresponsiveness ([Allen et al., 2009](#_ENREF_1); [Eschenbacher et al., 1991](#_ENREF_3)). No microbes or endotoxins (<1.0 EU/mL) were detected in the prepared inhalation formulations (Table S5).

.

Table S4 Characterization of the inhalation formulations

|  | Measured concentration (g/100g) | | | | % relative to expected concentration | | | |
| --- | --- | --- | --- | --- | --- | --- | --- | --- |
| Analysis | PG/VG/N | PG/VG/N/F-L | PG/VG/N/F-M | PG/VG/N/F-H | PG/VG/N | PG/VG/N/F-L | PG/VG/N/F-M | PG/VG/N/F-H |
| Propylene glycol | 69.5±1.4 | 64.5±2.3 | 61.8±2.3 | 53.3±2.3 | 97% | 95% | 96% | 94% |
| Vegetable glycerin | 17.6±0.7 | 16.2±0.8 | 16.0±0.6 | 13.3±0.3 | 99% | 95% | 99% | 94% |
| Nicotine | 2.0±0.0 | 2.0±0.1 | 2.0±0.1 | 2.0±0.0 | 99% | 99% | 100% | 99% |
|  | Measured concentration (µg/g) | | | | % relative to expected concentration | | | |
| 2-Methoxy-4-methylphenol | <LOD | 3566±136 | 6837±167 | 13281±228 | NA | 110% | 106% | 103% |
| Citronellol, D-L | <LOD | 516±15 | 999±35 | 1945±70 | NA | 114% | 110% | 107% |
| Ethyl maltol | <LOD | 3559±412 | 6675±733 | 12487±937 | NA | 110% | 103% | 96% |
| Methyl Anthranilate | <LOD | 565±47 | 1033±72 | 1854±83 | NA | 125% | 114% | 102% |
| Eugenyl Acetate | <LOD | 2639±90 | 5144±189 | 10018±331 | NA | 110% | 107% | 105% |
| Triethyl Citrate | <LOD | 472±67 | 919±119 | 1779±205 | NA | 104% | 101% | 98% |

Results shown are average and standard deviation of 4 batches of PG/VG/N inhalation formulation and 8 batches each of the low, medium, and high flavor-containing inhalation formulations. PG, propylene glycol; VG, vegetable glycerin; N, nicotine; F, flavors; L, low; M, medium; H, high; LOD, lower limit of detection.

Table S5 Results of pH measurement as well as microbial and endotoxin content in the inhalation formulations

|  | **Inhalation formulations** | | | |
| --- | --- | --- | --- | --- |
|  | **PG/VG/N** | **PG/VG/N/F-L** | **PG/VG/N/F-M** | **PG/VG/N/F-H** |
| pH | 9.5±0.04 (4) | 8.6±0.04 (8) | 8.3±0.03 (8) | 7.9±0.02 (8) |
| Microbiology (CFU/mL) | 0 (4) | 0 (7) | 0 (7) | 0 (7) |
| Endotoxin (Eu/mL) | <1.0 (4) | <1.0 (8) | <1.0 (8) | <1.0 (8) |

pH values are average and standard deviation of the data. Microbiology and endotoxin results shown are average of the data. The number of independent batches measured are indicated in parentheses. PG, propylene glycol; VG, vegetable glycerin; N, nicotine; F, flavors; L, low; M, medium; H, high.

**Characterization of the Test Atmosphere**

Table S6 Nicotine, TPM, PG, VG, and carbonyl concentrations in the exposure chambers

|  |  | **Concentration achieved in the test atmosphere (µg/L)** | | | | | | | | |
| --- | --- | --- | --- | --- | --- | --- | --- | --- | --- | --- |
| **Group** | **Sex** | **Nicotine** | **TPM** | **PG** | **VG** | **Formaldehyde** | **Acetaldehyde** | **Acrolein** | **Propionaldehyde** | **Crotonaldehyde** |
| Sham | Male | <LOD | -3.4 ± 2.5 (40) | <LOD | <LOD | 0.021 | 0.007 | <LOD | 0.004 | <LOD |
|  | Female | <LOD | -4.2 ± 2.9 (40) | <LOD | <LOD | 0.014 | 0.007 | <LOD | 0.005 | <LOD |
| 3R4F | Female | 16.0 ± 1.6 (39) | 287.5 ± 14.3 (40) | <LOD | 25.1 ± 2.5 (39) | 0.428 | 22.015 | 1.7014 | 1.383 | 0.4313 |
| PG/VG/N | Male | 15.7 ± 2.4 (39) | 932.1 ± 122.7 (40) | 514.3 ± 81.2 (39) | 185.3 ± 19.9 (39) | 0.038 | 0.038 | <LOD | 0.004 | <LOD |
|  | Female | 15.4 ± 2.5 (39) | 902.7 ± 128.7 (40) | 505.1 ± 81.6 (39) | 181.8 ± 20.5 (39) | 0.025 | 0.034 | 0.0005 | 0.004 | <LOD |
| PG/VG/N/F-L | Female | 15.5 ± 2.0 (39) | 860.8 ± 95.6 (40) | 483.7 ± 61.6 (39) | 161.0 ± 13.8 (39) | <LOD | 0.418 | 0.0009 | 0.010 | 0.0003 |
| PG/VG/N/F-M | Female | 15.5 ± 1.5 (39) | 834.8 ± 80.9 (40) | 451.8 ± 40.5 (39) | 153.2 ± 14.4 (39) | 0.039 | 0.786 | 0.0007 | 0.018 | 0.0006 |
| PG/VG/N/F-H | Male | 15.7 ± 2.0 (39) | 757.6 ± 83.9 (40) | 397.5 ± 49.3 (39) | 136.3 ± 10.8 (39) | 0.025 | 1.414 | 0.0007 | 0.003 | 0.0040 |
|  | Female | 15.4 ± 2.3 (39) | 747.3 ± 83.4 (40) | 378.2 ± 52.9 (39) | 141.5 ± 12.0 (39) | 0.033 | 1.476 | 0.0007 | 0.006 | 0.0040 |
| LOD | | 0.14 | NA | 0.47 - 0.48 | 1.05 - 1.08 | 0.007 | 0.007 | 0.0004 | 0.001 | 0.0002 |

Results shown are means ± standard deviation. The number of average daily concentrations are shown in parentheses. The TPM concentration in the sham chamber has a negative value because of the removal of moisture during sampling under slight negative pressure, which caused a decrease in the filter weight after sampling. PG, propylene glycol; VG, vegetable glycerol; N, nicotine; F, flavors; TPM, total particulate matter; NA, not applicable. LOD, lower limit of detection.

Table S7 Investigation of the contribution of 1,1-diethoxyethane to acetaldehyde detection

|  | **PG/VG** | **PG/VG/N/F-LL(1/16)** | **PG/VG/N/F-L(1/4)** | **PG/VG/N/F-H** | **PG/VG/N/F-H  (excluding 1,1-diethoxyethane)** |
| --- | --- | --- | --- | --- | --- |
| Amount of 1,1-diethoxyethane in inhalation formulation (µg/g)* | 0 | 243 | 971 | 3890 | 0 |
| Acetaldehyde  (µg/g)^#^ | 8.34 ± 0.89 | 202 ± 8 | 631 ± 131 | 4580 ± 370 | 24.6 ± 4.8 |

*Based on the weight of added material during preparation of the formulation. The L(1/4) formulation contained 1/4^th^ the amount of flavors in the PG/VG/N/F-H formulation. The LL(1/16) formulation contained 1/16^th^ the amount of flavors in the PG/VG/N/F-H formulation. ^#^Based on collected aerosol mass. PG, propylene glycol; VG, vegetable glycerol; N, nicotine; F, flavors; L, low; H, high.

Table S8 Aerosol/particle size distribution

| **Group** | **MMAD (µm) range** | **GSD range** | **Number of measurements** |
| --- | --- | --- | --- |
| 3R4F | 0.80–0.88 | 1.34–1.41 | 2 |
| PG/VG/N | 0.64–0.88 | 1.42–1.51 | 4 |
| PG/VG/N/F-L | 0.77–0.81 | 1.55 | 2 |
| PG/VG/N/F-M | 0.83–1.01 | 1.57 | 2 |
| PG/VG/N/F-H | 0.74–0.92 | 1.49–1.63 | 4 |

Results shown are the minimum and maximum values from 2 to 4 independent measurements. For the PG/VG/N/F-L and PG/VG/N/F-M groups, the same GSD was obtained in 2 independent measurements. MMAD, mass median aerodynamic diameter; GSD, geometric standard deviation; PG, propylene glycol; VG, vegetable glycerol; N, nicotine; F, flavors; L, low; M, medium; H, high.

**In-life** **observations**

Table S9 Clinical observations post-exposure

|  | **Total incidence (n, male)** | | | **Total incidence (n, female)** | | | | | |
| --- | --- | --- | --- | --- | --- | --- | --- | --- | --- |
| **Clinical Observation** | **Sham** | **PG/VG/N** | **PG/VG/N/F-H** | **Sham** | **3R4F** | **PG/VG/N** | **PG/VG/N/F-L** | **PG/VG/N/F-M** | **PG/VG/N/F-H** |
| Tremor, mild | 0 | 0 | 1 | 0 | 1 | 0 | 1 | 1 | 4 |
| Tremor, moderate | 0 | 0 | 3 | 0 | 0 | 0 | 0 | 0 | 5 |
| Piloerection | 0 | 0 | 0 | 0 | 1 | 0 | 1 | 0 | 1 |
| Reduced activity/exploration | 0 | 0 | 1 | 0 | 1 | 0 | 0 | 1 | 4 |
| Increased activity/exploration | 1 | 14 | 10 | 0 | 45 | 14 | 16 | 6 | 4 |
| Loss of responsiveness | 0 | 0 | 0 | 0 | 0 | 1 | 0 | 0 | 1 |
| Reduced grip strength, moderate | 0 | 0 | 2 | 0 | 0 | 0 | 0 | 0 | 1 |
| Hyperventilation, mild | 0 | 0 | 0 | 0 | 0 | 0 | 0 | 1 | 0 |
| Hunched position and/or lethargy | 0 | 0 | 0 | 0 | 4 | 0 | 1 | 0 | 0 |
| Hair loss | 0 | 0 | 0 | 0 | 0 | 0 | 1 | 2 | 0 |
| Microphthalmos, eye | 0 | 0 | 0 | 1 | 0 | 0 | 0 | 0 | 0 |
| Cataract, eye | 0 | 0 | 0 | 1 | 0 | 0 | 0 | 0 | 0 |
| Loss of transparency, eye | 0 | 0 | 0 | 1 | 0 | 0 | 0 | 0 | 0 |

Data shown are the sums of all incidences (n) of daily clinical observations recorded during the 5-week period. When indicated in the table of affected groups, tremor was observed between weeks 1 and 4. Piloerection was observed in week 1 or 3. Reduced activity/exploration and hyperventilation were observed in week 1. Increased activity/exploration was observed throughout the study period. Loss of responsiveness was observed in a PG/VG/N/F-H female mouse at week 1 and in a PG/VG/N female mouse at week 5. Reduced grip strength was observed at week 1 and/or 2. Hunched position/lethargy was observed at week 2 and/or 3. Stable clinical conditions such as cataract, microphthalmos, and loss of transparency of the eye were counted once per animal per study. PG, propylene glycol; VG, vegetable glycerol; N, nicotine; F, flavors; L, low; M, medium; H, high.

Table S10 Erythrocyte count and red blood cell indices in whole blood

|  | **Male** | | | **Female** | | | | | |
| --- | --- | --- | --- | --- | --- | --- | --- | --- | --- |
| **Parameter** | **Sham** | **PG/VG/N** | **PG/VG/N/F-H** | **Sham** | **3R4F** | **PG/VG/N** | **PG/VG/N/F-L** | **PG/VG/N/F-M** | **PG/VG/N/F-H** |
| Mean corpuscular hemoglobin (pg) | 14.98 ± 0.081 (10) | 14.79 ± 0.089 (10) | 14.81 ± 0.074 (10) | 15.2 ± 0.101 (10) | 16 ± 0.107 (10) *** | 15.1 ± 0.058 (10) | 15.11 ± 0.098 (10) +++ | 15.05 ± 0.089 (10) +++ | 15 ± 0.054 (10) +++ |
| Mean corpuscular volume (fL) | 44.72 ± 0.233 (10) | 44.55 ± 0.221 (10) | 44.75 ± 0.308 (10) | 45.82 ± 0.333 (10) | 47.92 ± 0.34 (10) *** | 45.28 ± 0.222 (10) | 45.61 ± 0.275 (10) +++ | 45.75 ± 0.284 (10) +++ | 45.52 ± 0.204 (10) +++ |

Counts and measurements are presented as mean with standard error of the mean. The number of individual animal measurements is shown in parentheses. *, ** and *** represent statistically significant differences between the treatment and sham groups at *p* ≤ 0.05, *p* ≤ 0.01 and *p* ≤ 0.001, respectively. +++ represents statistically significant differences between the PG/VG/N/F and 3R4F groups at *p* ≤ 0.001. #, ## and ### represent statistically significant differences between the PG/VG/N/F and PG/VG/N groups at *p* ≤ 0.05, *p* ≤ 0.01 and *p* ≤ 0.001, respectively. PG, propylene glycol; VG, vegetable glycerol; N, nicotine; F, flavors; H, high; M, medium; L, low.

Table S11 Leukocyte counts in whole blood

|  | **Male** | | | **Female** | | | | | |
| --- | --- | --- | --- | --- | --- | --- | --- | --- | --- |
| **Parameter** | **Sham** | **PG/VG/N** | **PG/VG/N/F-H** | **Sham** | **3R4F** | **PG/VG/N** | **PG/VG/N/F-L** | **PG/VG/N/F-M** | **PG/VG/N/F-H** |
| Leukocyte counts (10⁹/L) | 5.009 ± 0.852 (10) | 3.365 ± 0.519 (10) | 4.274 ± 0.586 (10) | 4.129 ± 0.508 (10) | 3.247 ± 0.236 (10) | 3.08 ± 0.355 (10) | 2.905 ± 0.262 (10) | 3.552 ± 0.513 (10) | 3.351 ± 0.605 (10) |
| Basophil counts (10⁹/L) | 0.01 ± 0.004 (10) | 0.013 ± 0.004 (10) | 0.01 ± 0.004 (10) | 0.011 ± 0.005 (10) | 0.009 ± 0.004 (10) | 0.017 ± 0.007 (10) | 0.005 ± 0.002 (10) | 0.013 ± 0.004 (10) | 0.012 ± 0.007 (10) |
| Eosinophil counts (10⁹/L) | 0.017 ± 0.008 (10) | 0.005 ± 0.002 (10) | 0.001 ± 0.001 (8) * | 0.011 ± 0.005 (10) | 0.008 ± 0.004 (10) | 0.003 ± 0.002 (10) | 0.014 ± 0.004 (8) # | 0.015 ± 0.01 (6) | 0.007 ± 0.003 (9) |
| Monocyte counts (10⁹/L) | 0.032 ± 0.012 (10) | 0.013 ± 0.005 (10) | 0.043 ± 0.016 (8) | 0.051 ± 0.015 (10) | 0.033 ± 0.018 (10) | 0.018 ± 0.007 (10) | 0.021 ± 0.009 (8) | 0.128 ± 0.095 (6) | 0.034 ± 0.008 (9) |
| Platelet (10⁹/L) | 860.9 ± 95.423 (10) | 996.7 ± 40.278 (10) | 985 ± 35.251 (10) | 904 ± 75.609 (10) | 892.4 ± 67.019 (10) | 939.4 ± 42.633 (10) | 891.8 ± 32.083 (10) | 818.6 ± 82.413 (10) | 852.5 ± 95.589 (10) |

Absolute counts are presented as mean with standard error of the mean. The number of individual animal measurements is shown in parentheses. * represents statistically significant differences between the treatment and sham groups at *p* ≤ 0.05. + and ++ represent statistically significant differences between the PG/VG/N/F and 3R4F groups at *p* ≤ 0.05 and *p* ≤ 0.01, respectively. # represents statistically significant differences between the PG/VG/N/F and PG/VG/N groups at *p* ≤ 0.05. PG, propylene glycol; VG, vegetable glycerol; N, nicotine; F, flavors; H, high; M, medium; L, low.

Table S12 Serum clinical chemistry results

|  | **Male** | | | **Female** | | | | | |
| --- | --- | --- | --- | --- | --- | --- | --- | --- | --- |
| **Parameter** | **Sham** | **PG/VG/N** | **PG/VG/N/F-H** | **Sham** | **3R4F** | **PG/VG/N** | **PG/VG/N/F-L** | **PG/VG/N/F-M** | **PG/VG/N/F-H** |
| Glucose (mmol/L) | 9.465 ± 0.356 (11) | 9.61 ± 0.5 (11) | 9.199 ± 0.433 (11) | 8.457 ± 0.526 (11) | 7.385 ± 0.433 (11) | 7.964 ± 0.364 (10) | 7.841 ± 0.563 (10) | 8.169 ± 0.448 (11) | 7.545 ± 0.303 (11) |
| Total cholesterol (mmol/L) | 1.425 ± 0.033 (11) | 1.424 ± 0.044 (11) | 1.476 ± 0.044 (11) | 1.255 ± 0.042 (11) | 1.275 ± 0.034 (11) | 1.212 ± 0.051 (10) | 1.339 ± 0.037 (10) | 1.261 ± 0.023 (11) | 1.266 ± 0.038 (11) |
| Triglyceride (mmol/L) | 0.926 ± 0.118 (11) | 0.916 ± 0.096 (11) | 0.726 ± 0.089 (11) | 0.737 ± 0.089 (11) | 0.764 ± 0.088 (11) | 0.661 ± 0.08 (10) | 0.771 ± 0.081 (10) | 0.778 ± 0.083 (11) | 0.719 ± 0.061 (11) |
| Total bilirubin (µmol/L) | 7.009 ± 0.268 (11) | 6.836 ± 0.397 (11) | 7.591 ± 0.289 (11) | 6.882 ± 0.523 (11) | 7.855 ± 0.178 (11) | 7.11 ± 0.49 (10) | 7.53 ± 0.201 (10) | 7.364 ± 0.573 (11) | 6.582 ± 0.374 (11) ++$ |

Results are mean with standard error of the mean. The number of individual animal measurements is shown in parentheses. * and ** represent statistically significant differences between the treatment and sham groups at *p* ≤ 0.05 and *p* ≤ 0.01, respectively. +, ++, and +++ represent statistically significant differences between the PG/VG/N/F and 3R4F groups at *p* ≤ 0.05, *p* ≤ 0.01, and *p* ≤ 0.001, respectively. #, ## represent statistically significant differences between the PG/VG/N/F and PG/VG/N groups at *p* ≤ 0.05 and *p* ≤ 0.01, respectively. $ represents statistically significant differences between the high and low flavor groups at *p* ≤ 0.05. PG, propylene glycol; VG, vegetable glycerol; N, nicotine; F, flavors; H, high; M, medium; L, low.

Table S13 Analysis of BALF analytes

|  | **Male** | | | | **Female** | | | | | |
| --- | --- | --- | --- | --- | --- | --- | --- | --- | --- | --- |
| **Parameter** | **Sham** | **PG/VG/N** | **PG/VG/N/F-H** | **Sham** | | **3R4F** | **PG/VG/N** | **PG/VG/N/F-L** | **PG/VG/N/F-M** | **PG/VG/N/F-H** |
| G-CSF (ng/L) | 4.425 ± 1.87 (10) | 3.245 ± 0.435 (10) | 3.22 ± 0.614 (10) | 3.567 ± 0.575 (10) | | 303.338 ± 122.921 (10) *** | 2.466 ± 0.46 (10) | 3.212 ± 0.398 (10) + | 3.493 ± 0.402 (10) +++ | 3.398 ± 0.405 (10) + |
| GM-CSF (ng/L) | 10.625 ± 1.412 (10) | 12.071 ± 1.337 (10) | 11.461 ± 1.064 (10) | 10.112 ± 0.789 (10) | | 21.908 ± 3.507 (10) ** | 12.283 ± 1.583 (10) | 13.477 ± 2.076 (10) | 12.016 ± 1.439 (10) + | 11.464 ± 1.46 (10) + |
| IFN-γ (ng/L) | 0.976 ± 0.18 (10) | 1.061 ± 0.122 (10) | 0.812 ± 0.064 (10) | 1.148 ± 0.35 (10) | | 2.602 ± 0.8 (10) | 1.4 ± 0.267 (10) | 0.942 ± 0.118 (10) | 0.978 ± 0.129 (10) | 1.323 ± 0.299 (10) |
| IL-10 (ng/L) | 6.492 ± 1.763 (10) | 6.114 ± 1.25 (10) | 7.533 ± 2.45 (10) | 8.051 ± 2.274 (10) | | 9.669 ± 3.202 (10) | 7.355 ± 2.179 (10) | 6.115 ± 1.314 (10) | 6.679 ± 1.438 (10) | 5.355 ± 1.928 (10) |
| IL-12 (ng/L) | 5.029 ± 0.642 (10) | 6.859 ± 1.839 (10) | 5.024 ± 0.816 (10) | 4.514 ± 1.07 (10) | | 6.358 ± 1.38 (10) | 4.109 ± 0.106 (10) | 5.26 ± 0.955 (10) | 4.455 ± 0.532 (10) | 5.928 ± 1.263 (10) |
| IL-12β (ng/L) | 7.174 ± 2.232 (10) | 7.386 ± 2.733 (10) | 8.045 ± 3.13 (10) | 13.389 ± 4.539 (10) | | 6.937 ± 2.539 (10) | 11.487 ± 3.907 (10) | 8.686 ± 3.239 (10) | 9.35 ± 2.923 (10) | 8.698 ± 3.231 (10) |
| IL-13 (ng/L) | 5.559 ± 0.908 (10) | 4.166 ± 0.807 (10) | 5.573 ± 1.025 (10) | 5.368 ± 1.059 (10) | | 5.468 ± 0.902 (10) | 5.966 ± 1.049 (10) | 3.753 ± 0.534 (10) | 3.934 ± 0.888 (10) | 4.538 ± 0.776 (10) |
| IL-15 (ng/L) | 4.63 ± 0.193 (10) | 5.641 ± 1.332 (10) | 4.442 ± 0.291 (10) | 4.665 ± 0.21 (10) | | 6.005 ± 1.448 (10) | 3.638 ± 0.443 (10) * | 4.944 ± 0.718 (10) | 5.504 ± 0.727 (10) # | 4.497 ± 0.272 (10) |
| IL-17 (ng/L) | 0.827 ± 0.147 (10) | 0.49 ± 0.071 (10) | 0.522 ± 0.068 (10) | 0.638 ± 0.057 (10) | | 6.793 ± 1.867 (10) ** | 0.682 ± 0.087 (10) | 0.707 ± 0.092 (10) ++ | 0.538 ± 0.077 (10) ++ | 0.584 ± 0.099 (10) +++ |
| IL-1α (ng/L) | 37.929 ± 6.134 (10) | 45.387 ± 8.648 (10) | 35.386 ± 8.416 (10) | 58.01 ± 12.322 (10) | | 43.317 ± 7.921 (10) | 48.359 ± 9.419 (10) | 39.094 ± 7.198 (10) | 35.529 ± 3.304 (10) | 28.665 ± 6.305 (10) * |
| IL-1β (ng/L) | 1.817 ± 0.334 (10) | 2.609 ± 0.363 (10) | 2.574 ± 0.458 (10) | 2.474 ± 0.365 (10) | | 6.504 ± 1.22 (10) ** | 2.456 ± 0.457 (10) | 2.407 ± 0.401 (10) ++ | 2.639 ± 0.487 (10) ++ | 3.268 ± 0.437 (10) + |
| IL-2 (ng/L) | 3.706 ± 0.566 (10) | 3.833 ± 0.802 (10) | 3.475 ± 0.832 (10) | 5.381 ± 1.299 (10) | | 2.564 ± 0.447 (10) * | 5.097 ± 1.022 (10) | 3.524 ± 0.631 (10) | 3.492 ± 0.421 (10) | 2.616 ± 0.594 (10) *# |
| IL-4 (ng/L) | 0.303 ± 0.075 (10) | 0.245 ± 0.017 (10) | 0.287 ± 0.039 (10) | 0.35 ± 0.058 (10) | | 1.249 ± 0.441 (10) | 0.25 ± 0 (10) | 0.237 ± 0.011 (10) ++ | 0.394 ± 0.17 (10) + | 0.257 ± 0.007 (10) + |
| IL-5 (ng/L) | 5.31 ± 1.939 (10) | 4.236 ± 1.313 (10) | 4.675 ± 1.874 (10) | 5.323 ± 1.911 (10) | | 15.138 ± 3.287 (10) * | 5.443 ± 1.733 (10) | 4.681 ± 1.993 (10) ++ | 5.665 ± 1.682 (10) + | 4.986 ± 2.025 (10) + |
| IL-6 (ng/L) | 2.478 ± 0.73 (10) | 2.654 ± 0.465 (10) | 2.03 ± 0.29 (10) | 2.117 ± 0.305 (10) | | 38.574 ± 10.208 (10) *** | 2.916 ± 0.513 (10) | 2.79 ± 0.384 (10) ++ | 2.463 ± 0.307 (10) ++ | 2.093 ± 0.237 (10) ++ |
| IL-7 (ng/L) | 0.916 ± 0.102 (10) | 1.183 ± 0.197 (10) | 1.409 ± 0.358 (10) | 1.641 ± 0.323 (10) | | 1.651 ± 0.521 (10) | 1.325 ± 0.246 (10) | 1.149 ± 0.126 (10) | 1.504 ± 0.197 (10) | 1.32 ± 0.196 (10) |
| IL-9 (ng/L) | 130.328 ± 14.749 (10) | 157.703 ± 25.756 (10) | 134.214 ± 21.194 (10) | 181.033 ± 30.394 (10) | | 87.438 ± 10.931 (10) * | 149.559 ± 18.026 (10) | 146.161 ± 22.412 (10) + | 145.304 ± 19.781 (10) + | 97.088 ± 14.525 (10) **#! |
| IP-10 (ng/L) | 10.089 ± 3.667 (10) | 6.131 ± 0.693 (10) | 5.627 ± 0.746 (10) | 7.998 ± 1.233 (10) | | 453.324 ± 137.322 (10) * | 6.234 ± 1.452 (10) | 6.315 ± 1.072 (10) ++ | 8.567 ± 2.606 (10) +++ | 5.202 ± 0.593 (10) +++ |
| KC (ng/L) | 34.124 ± 4.373 (10) | 26.137 ± 3.497 (10) | 28.345 ± 2.792 (10) | 33.775 ± 3.312 (10) | | 470.547 ± 67.871 (10) *** | 32.212 ± 4.818 (10) | 23.696 ± 2.546 (10) *+++ | 25.978 ± 1.457 (10) +++ | 24.689 ± 3.279 (10) +++ |
| MCP-1 (ng/L) | 15.885 ± 2.823 (10) | 13.062 ± 2.482 (10) | 8.581 ± 1.51 (10) | 16.955 ± 2.861 (10) | | 1916.747 ± 284.923 (10) *** | 12.591 ± 2.625 (10) | 16.684 ± 2.783 (10) +++ | 18.305 ± 4.538 (10) +++ | 11.164 ± 2.361 (10) +++ |
| MIP-1α (ng/L) | 10.797 ± 1.571 (10) | 13.011 ± 2.534 (10) | 12.264 ± 2.199 (10) | 14.861 ± 4.3 (10) | | 357.469 ± 83.828 (10) *** | 17.525 ± 4.423 (10) | 11.514 ± 1.814 (10) +++ | 13.293 ± 2.086 (10) ++ | 12.324 ± 2.574 (10) +++ |
| MIP-1β (ng/L) | 9.488 ± 0.514 (10) | 9.499 ± 0.71 (10) | 9.729 ± 0.457 (10) | 10.425 ± 0.336 (10) | | 515.174 ± 108.23 (10) *** | 9.385 ± 0.762 (10) | 8.361 ± 0.958 (10) ++ | 10.11 ± 0.334 (10) +++ | 9.971 ± 0.447 (10) ++ |
| MIP-2 (ng/L) | 27.548 ± 5.996 (10) | 26.87 ± 4.145 (10) | 19.615 ± 3.501 (10) | 32.872 ± 6.732 (10) | | 57.684 ± 13.188 (10) | 34.709 ± 7.395 (10) | 25.862 ± 3.884 (10) + | 22.448 ± 3.027 (10) + | 18.037 ± 2.604 (10) + |
| RANTES (ng/L) | 2.21 ± 0.163 (10) | 1.727 ± 0.113 (10) * | 1.637 ± 0.223 (10) | 1.963 ± 0.355 (10) | | 8.226 ± 1.407 (10) ** | 1.544 ± 0.329 (10) | 1.619 ± 0.16 (10) ++ | 1.456 ± 0.141 (10) +++ | 1.447 ± 0.241 (10) +++ |
| TNF-α (ng/L) | 2.089 ± 0.233 (10) | 2.674 ± 0.431 (10) | 1.82 ± 0.219 (10) | 2.23 ± 0.279 (10) | | 17.942 ± 2.906 (10) *** | 2.537 ± 0.248 (10) | 2.526 ± 0.318 (10) +++ | 2.233 ± 0.181 (10) +++ | 1.872 ± 0.17 (10) +++# |

Results are mean with standard error of the mean. The number of individual animal measurements is shown in parentheses. One BALF sample belonging to the male sham group had an unusually high total protein content (0.74 g/L, as opposed to the group average of 0.07 g/L) but not high lactate dehydrogenase activity (180 IU/L) or total free lung cell counts (6.31 x 10^5^). This may be related to the use of PBS–BSA instead of PBS by mistake during BALF collection. However, this outlier has no impact on the study conclusion. *, **, and *** represent statistically significant differences between the treatment and sham group at *p* ≤ 0.05, *p* ≤ 0.01, and *p* ≤ 0.001, respectively. +, ++, and +++ represent statistically significant differences between the PG/VG/N/F and 3R4F groups at *p* ≤ 0.05, *p* ≤ 0.01, and *p* ≤ 0.001, respectively. # represents statistically significant differences between the PG/VG/N/F and PG/VG/N groups at *p* ≤ 0.05. ! represents statistically significant differences between the high and medium flavor groups at *p* ≤ 0.05. PG, propylene glycol; VG, vegetable glycerol; N, nicotine; F, flavors; H, high; M, medium; L, low.

Table S14 Estimation of delivered dose and human equivalent dose

|  |  | **Delivered dose** | | **Human equivalent dose** | |
| --- | --- | --- | --- | --- | --- |
|  | **Average aerosol conc (µg/L)** | **µg analyte/day** | **mg analyte/(day x kg bw)** | **mg analyte/(day x kg bw)** | **mg analyte/day** |
| Nicotine | 15.6 | 129.2 | 6.5 | 0.5 | 31.6 |
| 2-methoxy-4-methylphenol | 9.9 | 82 | 4.1 | 0.3 | 19.9 |
| Citronellol | 1.6 | 13.2 | 0.7 | 0.1 | 3.4 |
| Ethyl maltol | 9.5 | 78.7 | 3.9 | 0.3 | 19.0 |
| Eugenyl acetate | 6.4 | 53 | 2.6 | 0.2 | 12.6 |
| Methyl anthranilate | 1.6 | 13.2 | 0.7 | 0.1 | 3.4 |
| Triethyl citrate | 1.6 | 13.2 | 0.7 | 0.1 | 3.4 |

Aerosol concentrations are the average nicotine concentration in all nicotine-containing groups, while flavor concentrations are the averages from the high flavor groups. The estimated delivered and human equivalent doses are calculated on the basis of the study exposure regimen, a body surface area conversion factor of 12.3 ([CDER, 2005](#_ENREF_2); [Reagan-Shaw et al., 2008](#_ENREF_5)), and assumptions of a minute volume of 0.023 L/min, bodyweight (bw) of 20 g for mice and 60 kg for human adult, and 100% analyte uptake. Under condition of this experimental setup, the assumption of 100% analyte uptake was based on very efficient nicotine uptake in CS ([Office of the Surgeon General, 2014](#_ENREF_4)), and potentially similar uptake of nicotine between CS and or e-vapor aerosol exposed groups (see 24-h urine nicotine metabolite data in Figure 2).

Table S15 Aerosol TPM yield

| **Group** | **Gender** | **CAG consumption rate (g/min)** | **Total air dilution (L/min)** | **Nominal TPM concentration (µg/L)** | **Achieved TPM concentration (µg/L)** | **Absolute TPM yield (%)** |
| --- | --- | --- | --- | --- | --- | --- |
| PGVGN | Male | 0.38 | 284 | 1340.2 | 932.1 | 70 |
| PGVGN | Female | 0.38 | 280 | 1358.6 | 902.7 | 66 |
| PGVGNF-L | Female | 0.35 | 279 | 1253.6 | 860.8 | 69 |
| PGVGNF-M | Female | 0.35 | 281 | 1246.0 | 834.8 | 67 |
| PGVGNF-H | Male | 0.38 | 286 | 1330.4 | 757.6 | 57 |
| PGVGNF-H | Female | 0.38 | 281 | 1353.8 | 747.3 | 55 |

TPM, total particulate matter; PG, propylene glycol; VG, vegetable glycerol; N, nicotine; F, flavors; H, high; M, medium; L, low.

Table S16 TPM yield captured at PIXE impactor

| **Group** | **Total weight collected at PIXE (g)** | **TPM based on mass collected at PIXE (µg/L)** | **Nominal TPM concentration (µg/L)** | **TPM yield on PIXE (%)** |
| --- | --- | --- | --- | --- |
| PG/VG/N | 0.00068 | 340.0 | 1358.6 | 25.0 |
| PG/VG/N/F-H | 0.00052 | 260.0 | 1353.8 | 19.2 |

TPM, total particulate matter; PG, propylene glycol; VG, vegetable glycerol; N, nicotine; F, flavors; H, high; M, medium; L, low.

Table S17 Aerosol trapping and transfer rates for flavor compounds

|  | **Trapping efficiencies** | | | **Trapping+transfer efficiencies** | | |  |
| --- | --- | --- | --- | --- | --- | --- | --- |
| **Compound name** | **Mean ratio** | **low** | **high** | **Mean ratio** | **low** | **high** | **Transfer efficiencies** |
| Isobutyraldehyde | 13% | -2% | 29% | <LOD | NA | NA | NA |
| 1-Penten-3-one | <LOD | NA | NA | <LOD | NA | NA | NA |
| Acetal | <LOD | NA | NA | <LOD | NA | NA | NA |
| Isoamyl alcohol | 73% | 51% | 95% | 83% | 61% | 106% | 113% |
| Ethyl lactate | NA | NA | NA | NA | NA | NA | NA |
| Ethyl-2-methylbutyrate | 49% | 30% | 70% | 48% | 30% | 68% | 97% |
| Z-3-Hexen-1-ol | 85% | 69% | 103% | 85% | 68% | 101% | 100% |
| 2-Methylbutanoic acid | 86% | 68% | 103% | 53% | 37% | 67% | 61% |
| 2,5-Dimethyl-pyrazine | 83% | 62% | 106% | 87% | 66% | 109% | 105% |
| *alpha*-Pinene | 14% | 3% | 26% | 12% | 1% | 24% | 84% |
| 3-Ethyl-pyridine | 83% | 66% | 101% | 83% | 66% | 100% | 100% |
| *para*-Cymene | 84% | 72% | 98% | 59% | 48% | 71% | 70% |
| 2-Acetylthiazole | 85% | 68% | 102% | 79% | 62% | 95% | 93% |
| Benzyl alcohol | 98% | 93% | 104% | 63% | 56% | 69% | 63% |
| Furaneol | 48% | 34% | 62% | 44% | 33% | 56% | 92% |
| Linalool | 89% | 72% | 105% | 81% | 66% | 97% | 92% |
| 2-Acetylpyrrole | 97% | 86% | 107% | 63% | 54% | 74% | 65% |
| Isopulegol | 86% | 71% | 101% | 78% | 64% | 92% | 90% |
| 1,4-Dimethoxy-benzene | 82% | 65% | 98% | 76% | 60% | 92% | 92% |
| Ketoisophorone | 89% | 77% | 102% | 78% | 66% | 90% | 87% |
| 2-Methoxy-4-methylphenol | 97% | 89% | 105% | 68% | 61% | 75% | 70% |
| DL-Citronellol | 89% | 80% | 99% | 70% | 61% | 79% | 78% |
| Piperitone | 92% | 82% | 104% | 71% | 61% | 81% | 77% |
| 2-Methyl-4-phenyl-2-butanol | 96% | 89% | 104% | 65% | 57% | 73% | 67% |
| Methyl anthranilate | 100% | 92% | 108% | 67% | 59% | 74% | 67% |
| Acetanisole | 97% | 87% | 106% | 60% | 51% | 69% | 62% |
| Methyl cinnamate | 95% | 85% | 106% | 65% | 51% | 77% | 68% |
| *p*-Mentha-8-thiol-3-one | 73% | 55% | 91% | <LOQ | NA | NA | NA |
| *alpha*-Damascone | 91% | 81% | 103% | 69% | 60% | 78% | 76% |
| *delta*-Nonalactone | 97% | 89% | 105% | 61% | 53% | 69% | 63% |
| Eugenyl acetate | 98% | 91% | 104% | 60% | 53% | 67% | 62% |
| Dihydroactinidiolide | 96% | 83% | 109% | 67% | 56% | 78% | 69% |
| Triethylcitrate | 98% | 85% | 111% | 69% | 57% | 80% | 70% |
| Ambrox | 99% | 92% | 107% | 64% | 57% | 71% | 64% |
| (E,Z)-2,6-Nonadienal | 95% | 76% | 113% | 84% | 70% | 98% | 88% |
| Ethyl maltol | 96% | 81% | 112% | 55% | 44% | 67% | 58% |
| Ethyl vanillin | 96% | 88% | 104% | 63% | 56% | 70% | 66% |

The trapping and trapping+transfer efficiencies were expressed as mean ratios with 95% confidence intervals (low and high values). Results were obtained from 9 independent sample analysis. Transfer efficiencies were expressed as trapping/(trapping+transfer) efficiencies. NA, not applicable: No trapping/transfer efficiencies could be obtained for 4 compounds, namely isobutyraldehyde, p-menth-8-thiol-3-one, 1-penten-3-one and acetal, as they were not trapped efficiently on Extrelut® NT 3 cartridges (values below LOQ or LOD). Transfer and trapping efficiencies of ethyl lactate was not reported due to low response and coelution during GC-MS spectrometry. LOQ, lower limits of quantification; LOD, lower limits of detection.

**Supplementary figures**

**
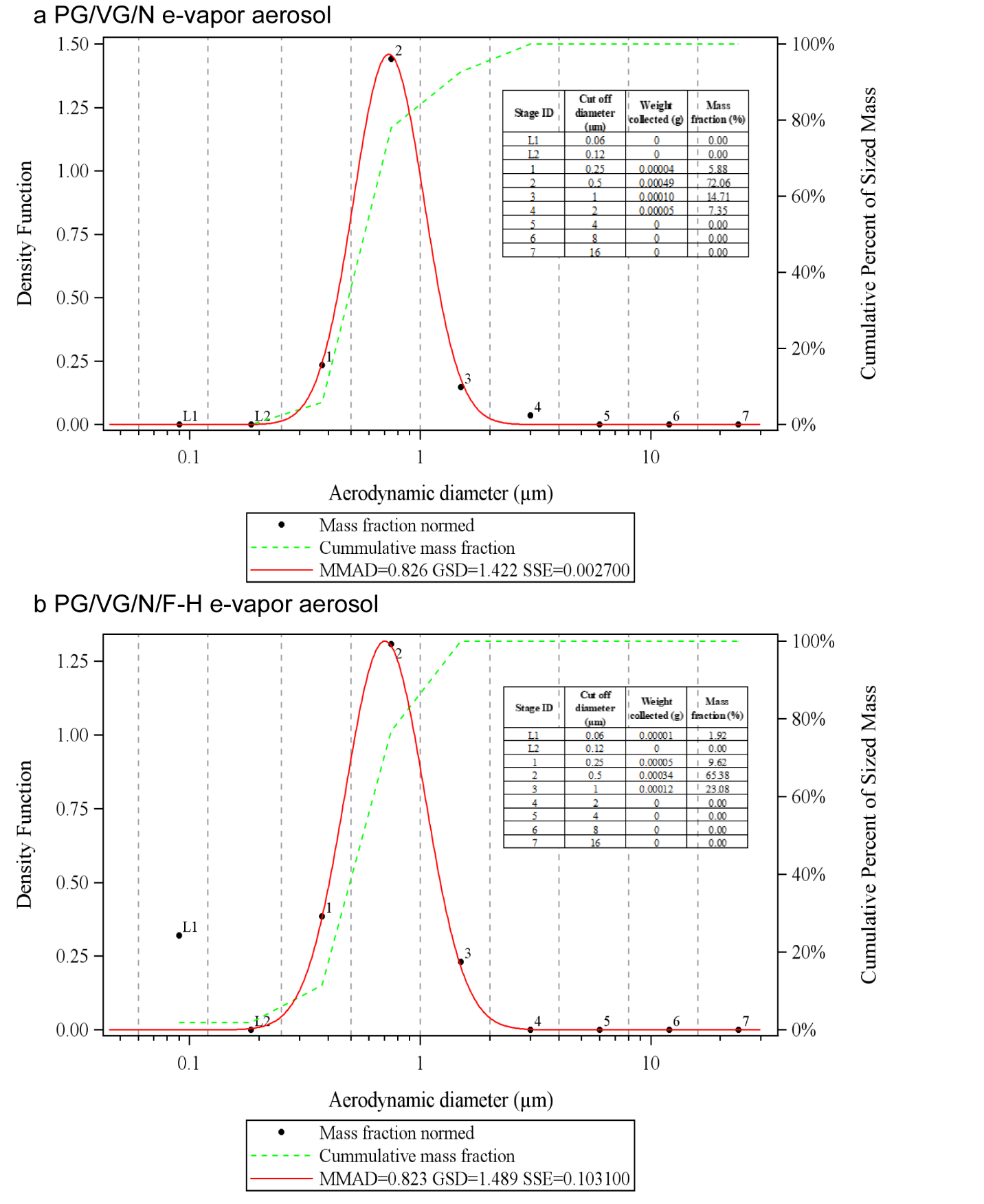
**

Figure S1 Plot of aerosol/particle size distribution

Plots of aerosol/particle size distribution are shown for (a) PG/VG/N and (b) PG/VG/N/F-H e-vapor aerosols. In the plots, the sizes and masses at each effective cut-off stage of the cascade impactor are indicated as L1, L2, 1, 2, 3, 4, 5, 6 and 7. Table inset, weight of collected mass and mass fraction at each stage are indicated. The red line represents the plot of aerosol/particle size distribution. The green dotted line shows the plot of the cumulative percentage of the collected mass. MMAD, mass median aerodynamic diameter; GSD, geometric standard deviation; SSE, sum of squares of the error; PG, propylene glycol; VG, vegetable glycerol; N, nicotine; F, flavors; H, high.


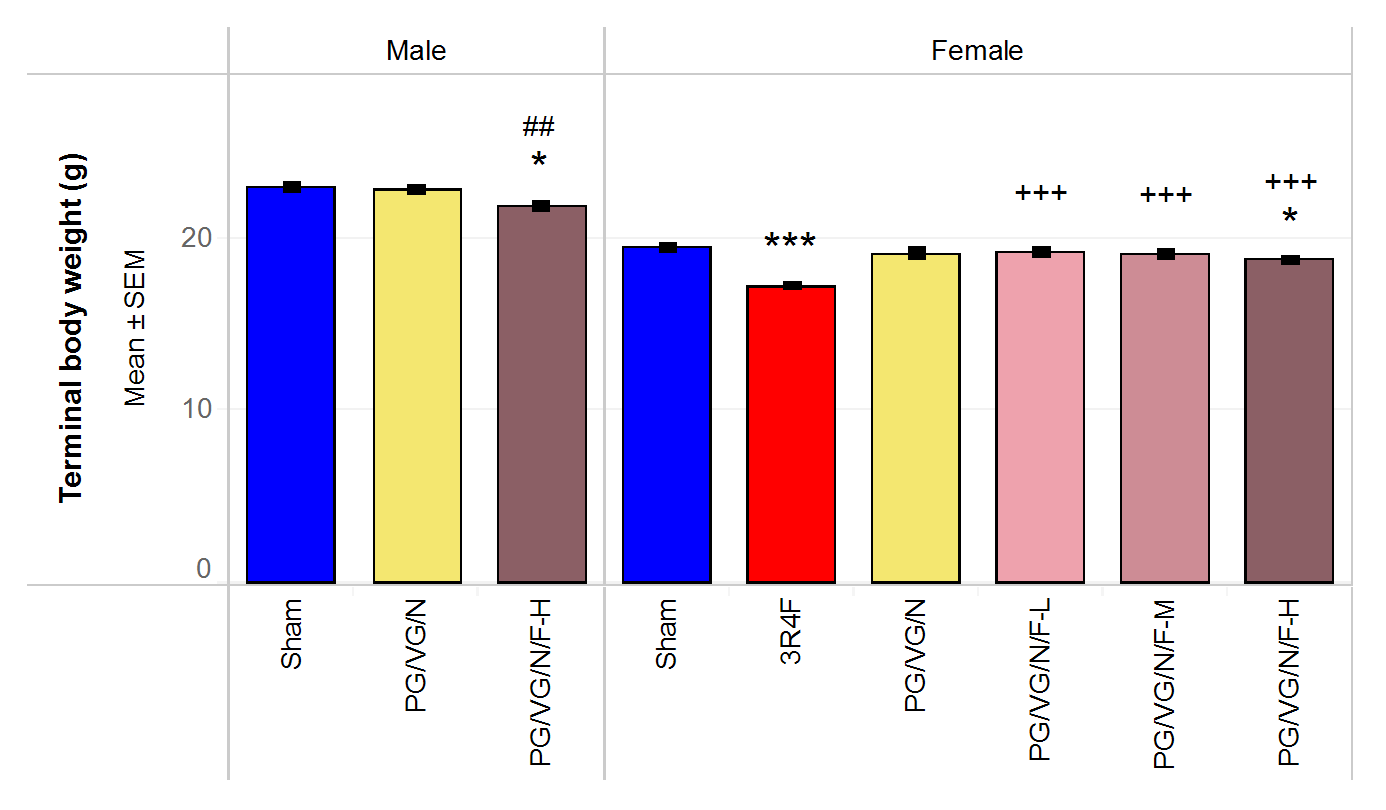


Figure S2 Terminal body weight

Terminal body weights are shown for male and female groups. Data are derived from 28 to 29 mice. * and *** represent statistically significant differences between the treatment and sham groups at *p* ≤ 0.05 and *p* ≤ 0.001, respectively. +++ represents statistically significant differences between the PG/VG/N/F and 3R4F groups at *p* ≤ 0.001, respectively. ## represents statistically significant differences between the PG/VG/N/F and PG/VG/N groups at *p* ≤ 0.01. PG, propylene glycol; VG, vegetable glycerol; N, nicotine; F, flavors; H, high; M, medium; L, low; SEM, standard error of mean.


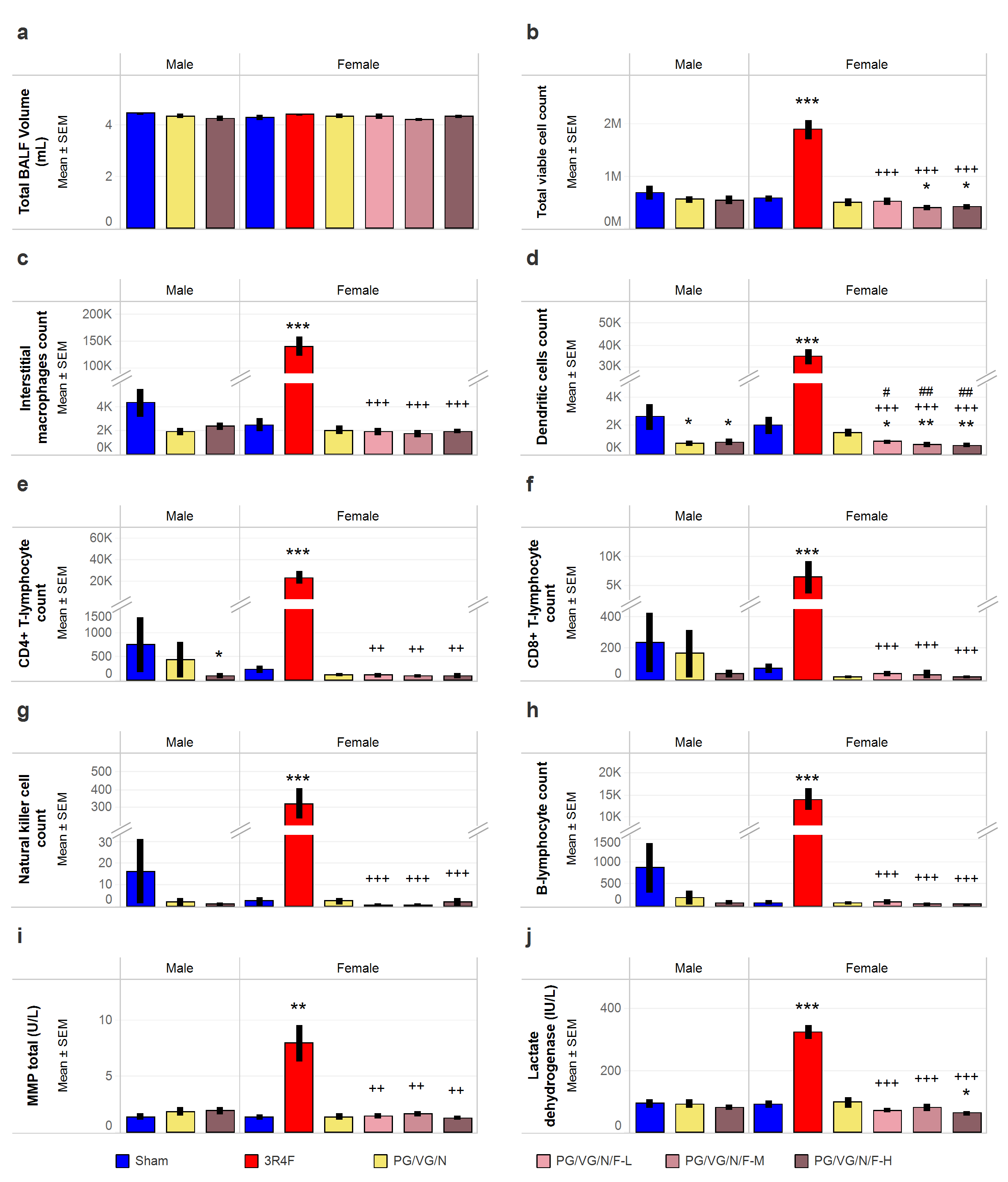


Figure S3 Results of BALF analysis

Data shown are (a) total recovered BALF volume, (b) total viable free lung cells, (c) interstitial macrophages, (d) dendritic cells, (e) CD4+ lymphocytes, (f) CD8+ lymphocytes, (g) NK cells, (h) B lymphocytes, (i) gelatinolytic (MMP) activity, and (j) LDH activity. Average cell counts and MMP/LDH activities were derived from 10 mice per group. *, **, and *** represent statistically significant differences between the treatment and sham groups at p ≤ 0.05, p ≤ 0.01, and p ≤ 0.001, respectively. + and ++ represent statistically significant differences between the PG/VG/N/F and 3R4F groups at p ≤ 0.05 and p ≤ 0.01, respectively. # and ## represent statistically significant differences between the PG/VG/N/F and PG/VG/N groups at p ≤ 0.05 and p ≤ 0.01, respectively. $ represents statistically significant differences between the high flavor and low flavor groups at p ≤ 0.05. For BALF inflammatory mediators, fold increases that are statistically significant (p ≤ 0.05) are shaded red, while fold reductions that are statistically significant (p ≤ 0.05) are shaded blue. PG, propylene glycol; VG, vegetable glycerol; N, nicotine; F, flavors; H, high; M, medium; L, low; BALF, bronchoalveolar lavage fluid; MMP, matrix metalloproteinase; LDH, lactate dehydrogenase. SEM, standard error of the mean.


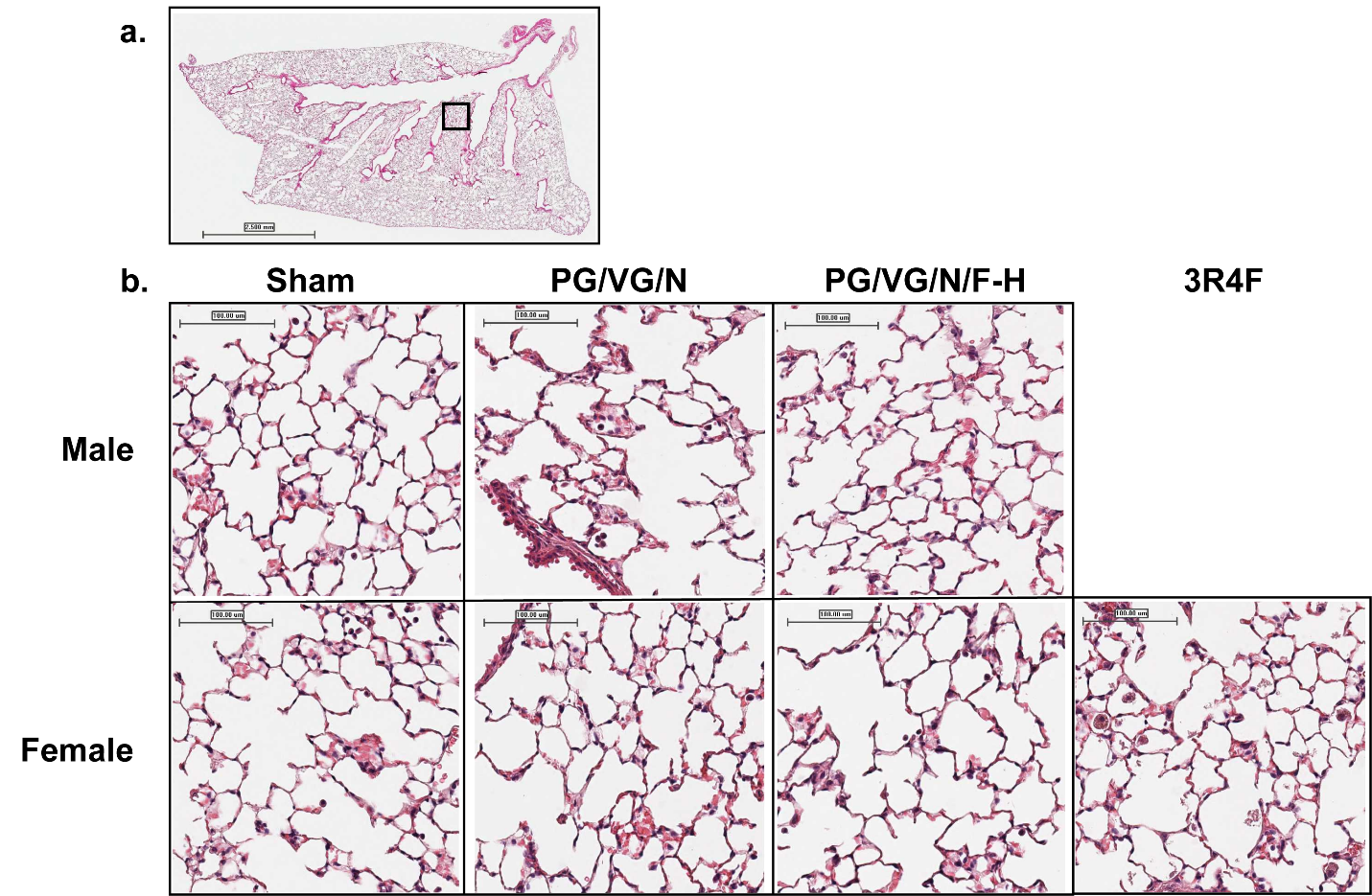


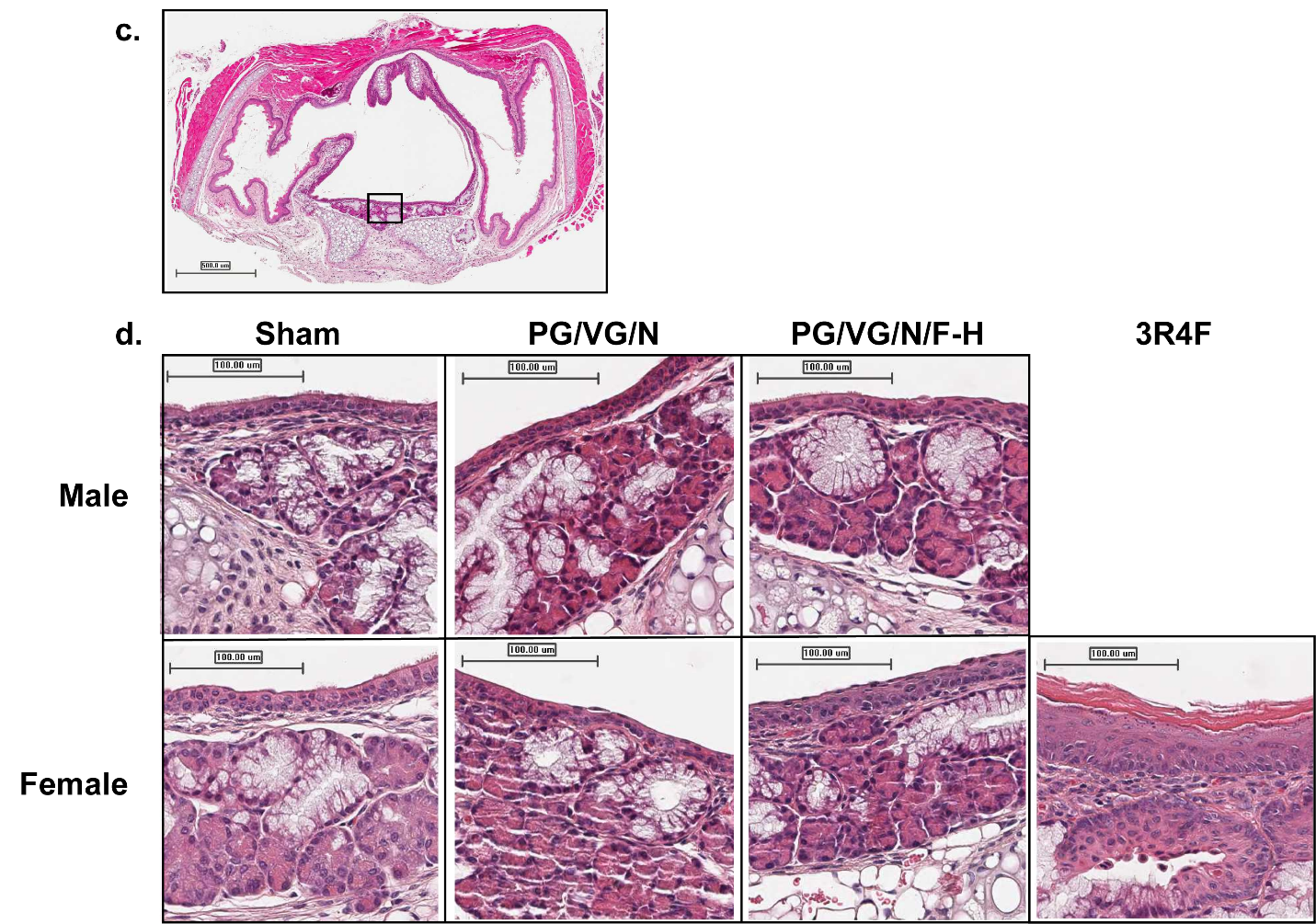


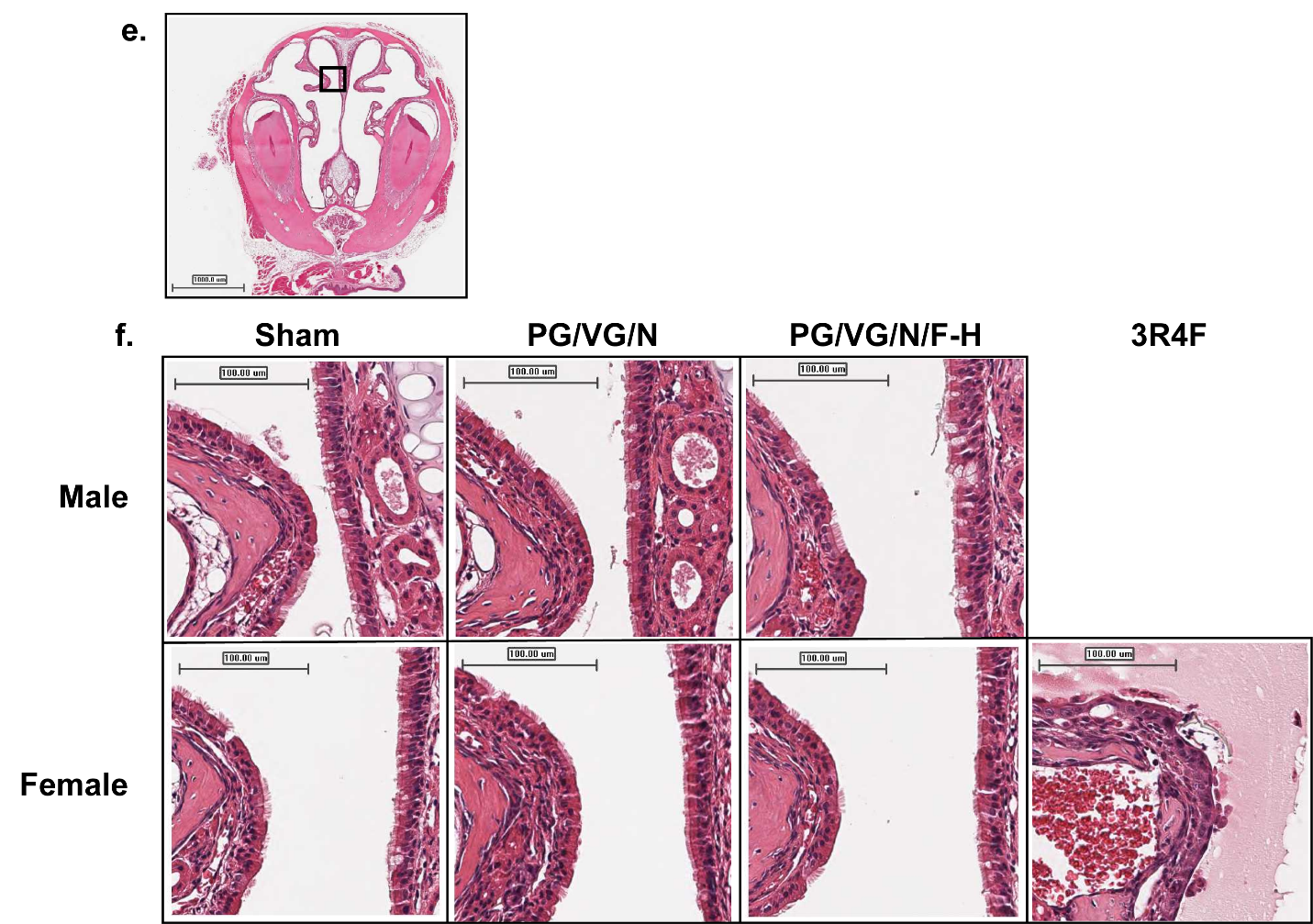


Figure S4 Representative histology images of the lung, larynx and nose sections

Hematoxylin–eosin stained sections of the left lung taken at (a) 1x and (b) 10x magnification, larynx (base of the epiglottis) taken at (c) 3.5x and (d) 20x magnification, and nose level 1 taken at (e) 1.5x and (f) 20x magnification. Inserted box in a, c, and e indicates the region of the organ where the microscopic images b, d, and f were taken. The inserted ruler is the size of the measuring ruler, indicated as (a) 2500 µm, (b, d, f) 100 µm, (c) 500 µm, and (e) 1000 µm.


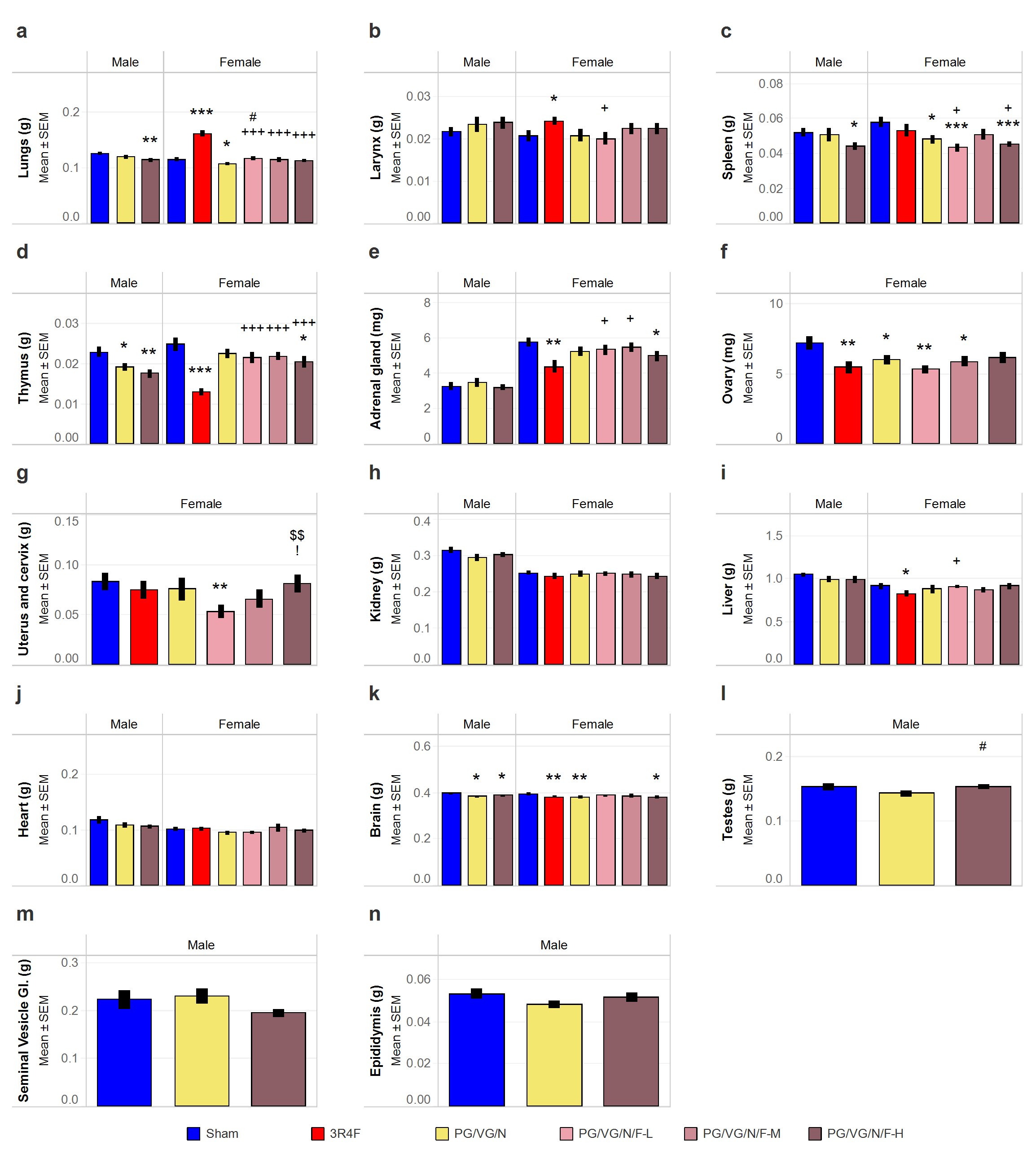


Figure S5 Absolute organ weights

Absolute organ weights were recorded for the (a) lungs, (b) larynx, (c) spleen, (d) thymus, (e) adrenal gland, (f) ovary, (g) uterus with cervix, (h) kidney, (i) liver, (j) heart, (k) brain, (l) testes, (m) seminal vesicle, and (n) epididymis. Data are absolute organ weights from 10 to 11 mice per group. *, **, and *** represent statistically significant differences between the treatment and sham groups at *p* ≤ 0.05, *p* ≤ 0.01, and *p* ≤ 0.001, respectively. + and +++ represent statistically significant differences between the PG/VG/N/F and 3R4F groups at *p* ≤ 0.05 and *p* ≤ 0.001, respectively. # represents statistically significant differences between the PG/VG/N/F and PG/VG/N groups at *p* ≤ 0.05. $$ represents statistically significant differences between the high flavor and low flavor groups at *p* ≤ 0.01. ! represents statistically significant differences between the high flavor and medium flavor groups at *p* ≤ 0.05. PG, propylene glycol; VG, vegetable glycerol; N, nicotine; F, flavors; L, low; M, medium; H, high; SEM, standard error of mean.


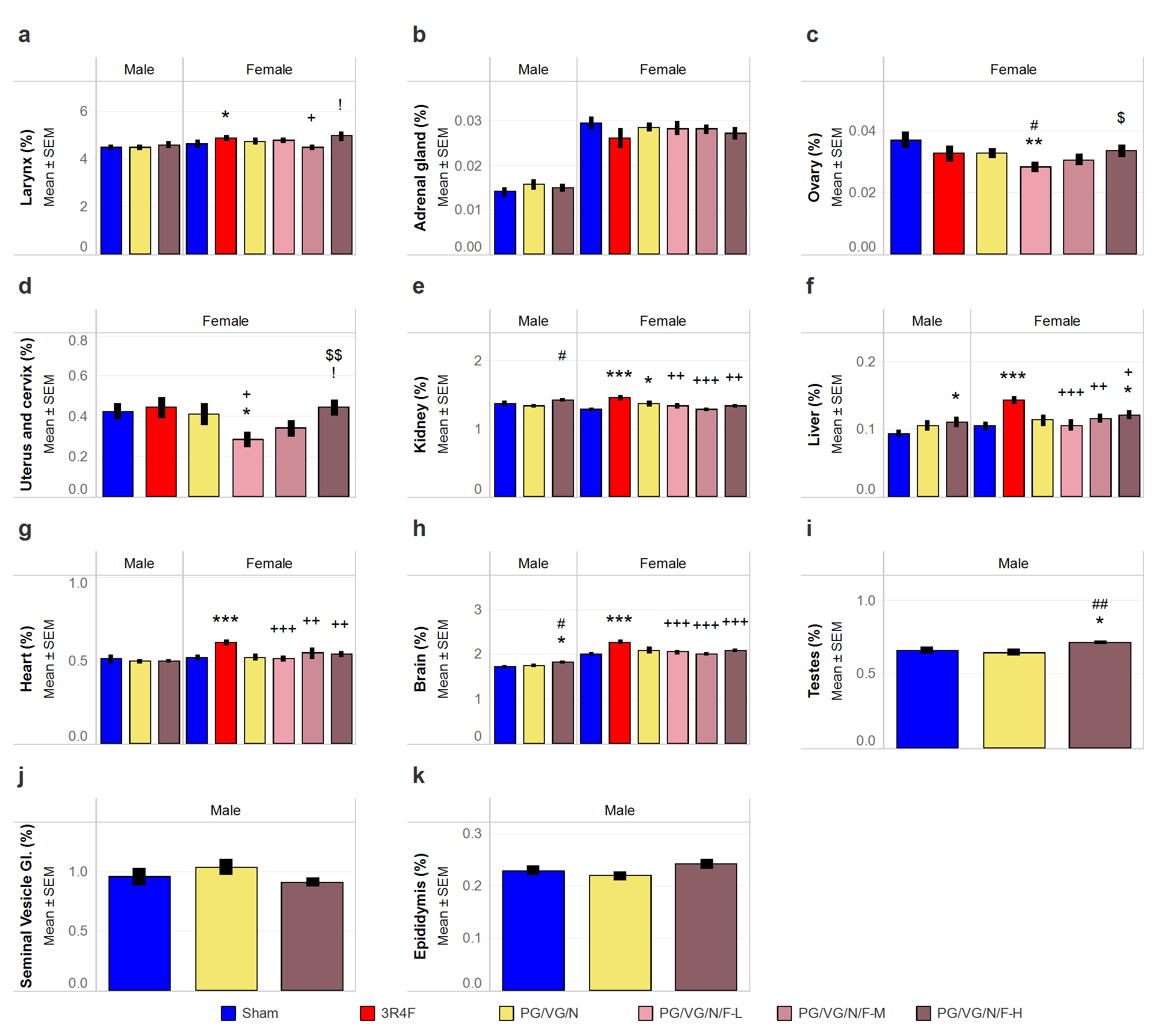


Figure S6 Organ weights relative to bodyweight.

Relative organ weights were recorded for the (a) larynx, (b) adrenal glands, (c) ovary, and (d) uterus with cervix, (e) kidney, (f) liver, (g) heart, (h) brain, (i) testes, (j) seminal vesicle, and (k) epididymis. Data are absolute organ weights from 10 to 11 mice per group. *, **, and *** represent statistically significant differences between the treatment and sham groups at *p* ≤ 0.05, *p* ≤ 0.01, and *p* ≤ 0.001, respectively. +, ++, and +++ represent statistically significant differences between the PG/VG/N/F and 3R4F groups at *p* ≤ 0.05, *p* ≤ 0.01, and *p* ≤ 0.001, respectively. # and ## represent statistically significant differences between the PG/VG/N/F and PG/VG/N groups at *p* ≤ 0.05 and *p* ≤ 0.01. $ and $$ represent statistically significant differences between the high flavor and low flavor groups at *p* ≤ 0.05 and *p* ≤ 0.01. ! represents statistically significant differences between the high flavor and medium flavor groups at *p* ≤ 0.05. PG, propylene glycol; VG, vegetable glycerol; N, nicotine; F, flavors; L, low; M, medium; H, high; SEM, standard error of mean.


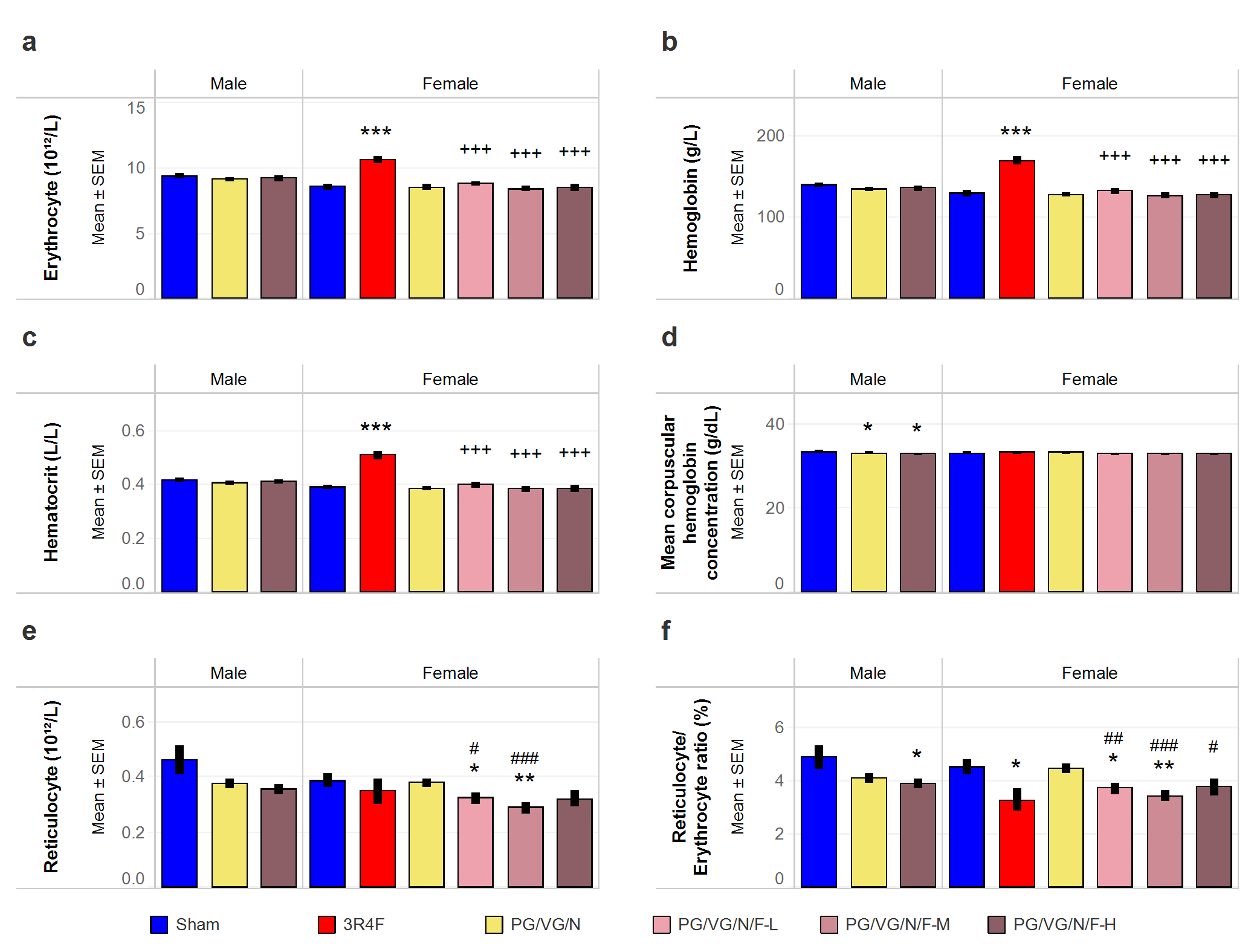


Figure S7 Red blood cell parameters.

Results of whole blood hematological analysis are shown for (a) erythrocyte counts, (b) hemoglobin concentration, (c) hematocrit, (d) mean corpuscular hemoglobin concentration, (e) reticulocyte, and (f) relative reticulocyte counts. Data shown are from 10 mice per group, from samples collected during terminal dissection. *, **, and *** represent statistically significant differences between the treatment and sham groups at *p* ≤ 0.05, *p* ≤ 0.01, and *p* ≤ 0.001, respectively. +++ represents statistically significant differences between the PG/VG/N/F and 3R4F groups at *p* ≤ 0.001. #, ##, and ### represent statistically significant differences between the PG/VG/N/F and PG/VG/N groups at *p* ≤ 0.05, *p* ≤ 0.01, and *p* ≤ 0.001, respectively. PG, propylene glycol; VG, vegetable glycerol; N, nicotine; F, flavors; L, low; M, medium; H, high; SEM, standard error of mean.

**References**

Allen, G. B., Leclair, T. R., von Reyn, J., Larrabee, Y. C., Cloutier, M. E., Irvin, C. G., & Bates, J. H. (2009). Acid aspiration-induced airways hyperresponsiveness in mice. *J Appl Physiol (1985), 107*(6), 1763-1770. doi:10.1152/japplphysiol.00572.2009

CDER. (2005). Guidance for industry: Estimating the maximum safe starting dose in initial clinical trials for theraperutics in adult healthy volunteers *Food and Drug Administration. Center for Drug Evaluation and Research.*, 1-27.

Eschenbacher, W. L., Gross, K. B., Muench, S. P., & Chan, T. L. (1991). Inhalation of an alkaline aerosol by subjects with mild asthma does not result in bronchoconstriction. *American Review of Respiratory Disease, 143*(2), 341-345. doi:10.1164/ajrccm/143.2.341

Office of the Surgeon General. (2014). The health consequences of smoking—50 years of progress: a report of the surgeon general.

Reagan-Shaw, S., Nihal, M., & Ahmad, N. (2008). Dose translation from animal to human studies revisited. *FASEB Journal, 22*(3), 659-661. doi:10.1096/fj.07-9574LSF

Sciuscio, D., Calvino-Martin, F., Kumar, A., Langston, T. B., Martin, E., Marescotti, D., . . . Lee, K. M. (Accepted). Toxicological Assessment of Flavor Ingredients in E-vapor Products. *Frontiers in Toxicology*.

Sciuscio, D., Ehman, K., Langston, T., Kumar, A., Lee, K., Marescotti, D., . . . Vanscheeuwijck, P. (2020). *A Structure-Based Grouping Approach for Predicting Biological Activity of Flavor Ingredients Contained in E-vapor Products. Annual Meeting Abstract Supplement. Abstract no. 3186.* Paper presented at the Society of Toxicology 59th Annual Meeting.

Smith, C. (2019). *Preclinical testing of flavors in e-vapor products, part 2: Preparation and stability characterization of representative flavor mixtures. Abstract 104.* Paper presented at the TSRC, Tob. Sci. Res. Conf.

USP41. (2018). Bacterial endotoxins test.

Wong, E. T., Luettich, K., Krishnan, S., Wong, S. K., Lim, W. T., Yeo, D., . . . Peitsch, M. C. (2020). Reduced Chronic Toxicity and Carcinogenicity in A/J Mice in Response to Life-Time Exposure to Aerosol from a Heated Tobacco Product Compared with Cigarette Smoke. *Toxicological Sciences, 178*(1), 44-70.
